# Nanotechnology’s frontier in combatting infectious and inflammatory diseases: prevention and treatment

**DOI:** 10.1038/s41392-024-01745-z

**Published:** 2024-02-21

**Authors:** Yujing Huang, Xiaohan Guo, Yi Wu, Xingyu Chen, Lixiang Feng, Na Xie, Guobo Shen

**Affiliations:** Department of Biotherapy, Cancer Center and State Key Laboratory of Biotherapy, West China Hospital, and West China School of Basic Medical Sciences and Forensic Medicine, Sichuan University, and Collaborative Innovation Center for Biotherapy, Chengdu, 610041 China

**Keywords:** Vaccines, Biomaterials

## Abstract

Inflammation-associated diseases encompass a range of infectious diseases and non-infectious inflammatory diseases, which continuously pose one of the most serious threats to human health, attributed to factors such as the emergence of new pathogens, increasing drug resistance, changes in living environments and lifestyles, and the aging population. Despite rapid advancements in mechanistic research and drug development for these diseases, current treatments often have limited efficacy and notable side effects, necessitating the development of more effective and targeted anti-inflammatory therapies. In recent years, the rapid development of nanotechnology has provided crucial technological support for the prevention, treatment, and detection of inflammation-associated diseases. Various types of nanoparticles (NPs) play significant roles, serving as vaccine vehicles to enhance immunogenicity and as drug carriers to improve targeting and bioavailability. NPs can also directly combat pathogens and inflammation. In addition, nanotechnology has facilitated the development of biosensors for pathogen detection and imaging techniques for inflammatory diseases. This review categorizes and characterizes different types of NPs, summarizes their applications in the prevention, treatment, and detection of infectious and inflammatory diseases. It also discusses the challenges associated with clinical translation in this field and explores the latest developments and prospects. In conclusion, nanotechnology opens up new possibilities for the comprehensive management of infectious and inflammatory diseases.

## Introduction

Inflammation is an adaptive biological response of the immune system to harmful stimuli, such as infections and tissue damage.^1,2^ Acute inflammation serves as the initial self-defense response of the body to pathogen infections or injuries, during which immune cells and inflammatory factors collaborate to efficiently clear pathogens, repair tissues, and restore homeostasis.^3,4^ If the inflammatory response is not promptly terminated, it may progress into chronic inflammation, aggravating tissue damage, and infectious diseases.^5^ Chronic inflammation appears to not arise directly from typical injuries or infections but more from dysfunctions in the immune system and disruptions in bodily homeostasis.^3,6^ In contrast to the beneficial and important role of moderate inflammation in host defense, harmful chronic inflammation results in a variety of chronic inflammatory diseases, including autoimmune diseases, allergic conditions, atherosclerosis(AS), and even an increased risk of cancer.^1,7^ The majority of autoimmune therapies or wide-ranging immune suppressors are supportive to slow the progression of the illness and symptoms.^8^ However, conventional drugs for inflammation diseases, like inflammatory bowel disease (IBD), are ineffective therapeutically and have serious side effects.^9^ Also, there are still no efficient or secure drugs available for clinical treatment of some inflammatory diseases, like stroke that is the leading cause of mortality and disability globally.^10^ Therefore, further elucidating the pathogenesis of chronic inflammation and developing more effective targeted drugs is an urgent priority for the treatment of inflammatory diseases.

In recent years, nanotechnology has emerged as a promising field with significant potential in combating infectious and inflammatory diseases. NPs with unique properties and capabilities have been explored for applications in vaccine development, antiviral drug delivery and pathogen detection. Currently, nanostructured viral vaccines based on virus-like particles (VLPs) have been widely deployed worldwide for viruses like severe acute respiratory syndrome coronavirus 2 (SARS-CoV-2), human papillomavirus (HPV), hepatitis B virus (HBV), and influenza. Notably, for the highly contagious SARS-CoV-2 virus that has caused a global pandemic, various COVID-19 vaccines have been developed using both traditional inactivated viruses and nanotechnology-based approaches, such as the BioNTech/Pfizer and Moderna messenger RNA (mRNA) vaccines, Novavax’s VLP protein vaccine.^11^ Some nanomaterials, such as silver nanoparticles (AgNPs), selenium nanoparticles (SeNPs), and metal NPs solutions (ND50, NK99, and TPNT1), can be prepared as environmental sanitizers or as preventive or therapeutic inhalants due to their directive antibacterial or antiviral effects in vitro.^12^ Moreover, NPs can be utilized for the delivery of drugs, enhancing their efficacy and reducing adverse reactions. It was shown that ethyl cellulose nanoparticles (EC-NPs) for amphotericin delivery had good stability, high bioavailability, and low cytotoxicity, providing a potential delivery vehicle for oral drugs for the treatment of fungi and parasite infections.^13^ Nanotechnology-based detection platforms have been developed to identify pathogens, offering rapid and sensitive diagnostics. A polyethyleneimine-assisted copper in situ growth strategy demonstrated excellent sensitivity, precision and repeatability for the detection of infectious diseases, such as *E. coli* and SARS-CoV-2 infections.^14^ These applications of nanomaterials present new opportunities to improve prevention strategies and enhance the effectiveness of therapies for infectious diseases.

In addition, nanotechnology is an effective approach to achieve therapeutic goals for inflammatory diseases, owing to its high drug loading capacity, efficient targeting, controllable sustained release, and ability to cross physiological barriers. When interferon (IFN)-β therapy was combined with NPs, like IFN-carried chitosan/sulfobutylether-cyclodextrin NPs, it was successful in intranasal administration of IFN-β into the central nervous system (CNS), boosting clinical improvement and controlling neurological inflammation in encephalomyelitis (EAE).^15^ Moreover, nanomaterials can also serve as molecular probes to provide support for imaging diagnosis of inflammatory diseases. Prussian blue NPs have been successfully utilized in magnetic resonance imaging (MRI) imaging to accurately concentrate and identify rheumatoid arthritis (RA).^16^ Therefore, nanotechnology provides the potential for treat-to-target principles, serving as the cornerstone of inflammatory disease treatment.

While nanotechnology holds tremendous potential in the fight against inflammation-associated diseases, some challenges and issues must be addressed as it progresses toward clinical applications. Further research is needed to improve the safety, stability, scalability, and efficiency of nanotechnology-based prevention and treatment approaches. Here, we provide an overview of the latest research advancements and applications of nanotechnology in infectious and inflammatory diseases, encompassing areas such as vaccine development, therapeutic drug delivery, and disease detection. Besides, we discuss the current challenges and limitations in its applications, hoping that the insights will offer valuable recommendations for the development of innovative strategies for the comprehensive prevention and treatment of infectious and inflammatory diseases.

## Advanced nanotechnologies

**Fig. 1:**
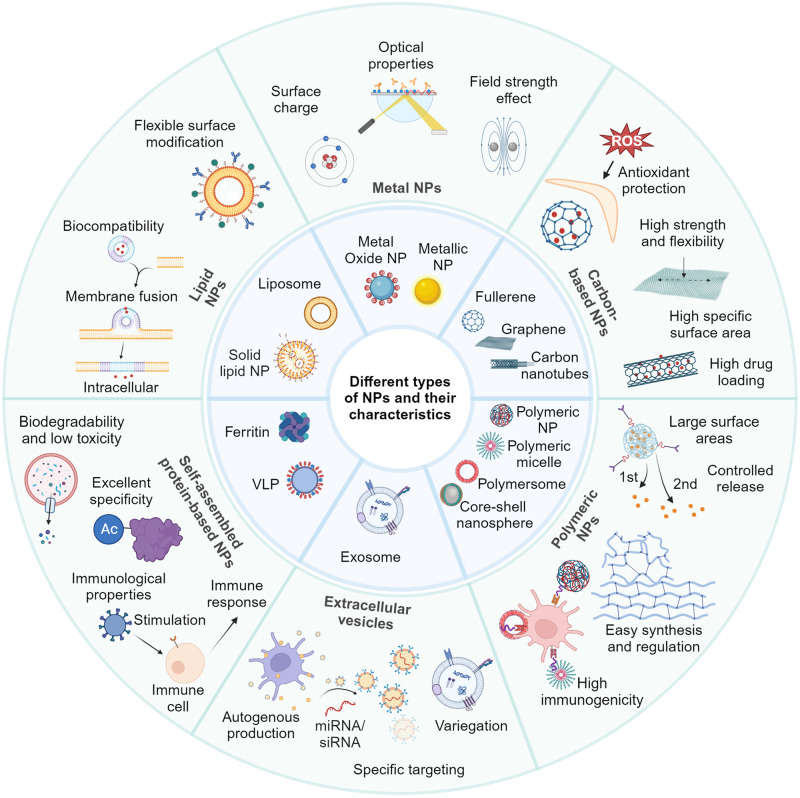
Six common nanomaterials and their characteristics. Lipid NPs, composed of lipids such as phospholipids, exhibit good biocompatibility and flexible surface modification capabilities. Metal NPs, including metals (such as gold, silver, copper) and their metal oxides, possess excellent optical, electronic, and magnetic properties, enabling applications in biological imaging, PTT, and sensing.^790^ Carbon-based nanomaterials, including CNTs, graphene, and fullerenes, not only have a large surface area and high drug loading capacity but also exhibit high strength and chemical stability, allowing resistance to oxidative environments.^791^ Polymer NPs, composed of polymer materials, display diverse structures and properties.^792^ Self-assembled NPs, including ferritin family proteins and VLPs, possess good biodegradability in the case of the former and can mimic viral stimuli to initiate immune responses in the case of the latter.^793^ Exosomes, a type of small vesicles secreted by cells, carry abundant proteins, nucleic acids, and signaling molecules, playing vital roles in information transfer and regulation.^794^ These and exosomes have broad applications in the biomedical and nanotechnology fields, including drug delivery, molecular imaging, biosensing, tissue engineering, and disease diagnosis

The various characteristics of NPs enable diverse applications in biomedicine. Nanomaterials can serve as adjuvants and vaccine delivery vectors to enhance vaccine-induced specific immune responses and antigen immunogenicity, and are widely used for infectious disease prevention, tumor immunotherapy, etc. Meanwhile, nanomaterials, like lipid nanoparticles (LNPs), polymeric NPs, and exosomes, can act as delivery systems for targeted drug distribution, controlled release, and effective treatment.^17^ By fine-tuning their surface functional groups, nanomaterials like magnetic NPs and quantum dots (QDs) can be used in biomedical imaging, providing high-sensitivity detection of specific targets and real-time monitoring of disease progression.^18,19^ In addition, nanomaterials with antibacterial and antiviral properties are integrated into protective equipment like masks, gloves, and disinfectants, serving as wound dressings to prevent infections. Here, we delineated the characteristics of each NP variant (Table 1), particularly focusing on their application in the prevention, treatment, and detection/diagnosis of infectious and inflammatory diseases (Fig. 1).

**Table 1:** Advantages and disadvantages of existing NPs in drug delivery and vaccines

Types of NPs	Composition	Advantages	Disadvantages	Examples of applications	Ref
Lipid NPs	Liposomes, commonly used lipids, include lecithin, triglycerides, triglycerides of palm stearate, and fatty acids	High biocompatibility; strong drug loading capacity; flexible surface modification	Limited stability under certain environmental conditions; limited control of drug release rate; costly preparation, potential toxicity at high dosages	Targeting lung therapy COVID-19	^744^
				Making mRNA vaccines against Zika virus infection	^745^
				Vitamin lipid nanoparticles can be used to treat septicaemia caused by drug-resistant bacteria	^746^
				Glycyrrhetinic acid-lipid framework nanocarriers improve drug loading efficiency of anti-hepatocellular carcinoma drugs	^747^
Metal NPs	Metal and metal oxide NPs, including silver, gold, CuO, SiO_2_, TiO_2_ and various other metal oxides	Unique shape, size, structure, and local-field enhancement action	Potential toxicity; limited stability with aggregation and morphological changes; environmental pollution concerns; limited degradation in vivo	Intravaginal zinc oxide tetrapod NPs against genital herpes	^668^
				AgNPs on H1N1 inhibit influenza A virus	^748^
				Delivery of antiviral siRNA with AuNPs inhibits dengue virus infection	^749^
				Cuprous oxide NPs against Hepatitis C Virus	^750^
Carbon-based NPs	Carbon nanotubes	Large specific surface area and hollow structure; increasing application capability by surface modification; good chemical and physical stability	Pulmonary toxicity; complex preparation steps; varying diameters, lengths, structures	Multiwalled carbon nanotubes for the detection of zooplankton in water	^751^
	Graphene	Excellent mechanical properties; high strength and flexibility; high specific surface area	Limitation in stability; aggregation in aqueous solution affects stabilization and release; potential biotoxicity issues	Sulfonated MNPs functionalized destroy herpes simplex virus type 1.	^752^
	Fullerenes	Antioxidant properties; stable structure; surface modification to obtain multiple properties	Relatively low load capacity; low solubility in water	Fullerene derivatives inhibit HIV by complexing with HIV protease.	^753^
				C60-β-cyclodextrin conjugate improves nuclear transport of doxorubicin	^754^
Polymeric NPs	Natural hydrophilic polymers and synthetic hydrophobic polymers	Good drug loading capacity and controlled release capabilities; easy synthesis and regulation	Long-term toxicity from body accumulation; potentially toxic degradation products； complex preparation and functionalization	Porous PLA and PLGA NPs for pulmonary delivery of HBV vaccine	^105,755^
				(PEG-b-PLA) NPs improve protein affinity for delivered drugs	^756^
Protein NPs	VLP	High structural stability and resistance to degradation; immunocompatibility; biomimetic properties	Inefficient protein delivery in vivo; insufficient immunogenicity, requiring adjuvants and multiple injections for vaccination; complex preparation process	Novel virus-like particle vaccine encoding the circumsporozoite protein of plasmodium falciparum is Immunogenic.	^757^
				Engineered VLPs for efficient delivery of therapeutic proteins.	^758^
	Proteins	Good biocompatibility and biodegradability; multifunctionality through surface modification.	Complex preparation and functionalization; high production costs; limited drug loading capacity	Dual-sensitive antibacterial peptide nanoparticles prevent dental caries.	^759^
				Development of spike RBD ferritin proteins vaccine against SARS-CoV-2 infection in ferrets	^760^
Exosomes	Classification according to source	Excellent biocompatibility; targetability	Difficulty in standardized production; poor experimental reproducibility; characterization difficulties; heterogeneity; Harsh storage conditions	Recombinant SARS-CoV-2 receptor-binding structural domain-modified exosomes as inhalable COVID-19 vaccines.	^143^

*VLP* virus-like particles, *SARS-CoV-2* severe acute respiratory syndrome coronavirus 2, *HIV* human immuno-deficiency virus, *siRNA* small interfering RNA, *AgNPs* silver nanoparticles, *AuNPs* gold nanoparticles, *MNPs* magnetic nanoparticles, *HBV* hepatitis B virus, *PLGA* Poly(lactide-co-glycolic) acid, *PEG* polyethylene glycol, *PLA* Poly(lactic acid), *RBD* receptor-binding domain

### Metal NPs

Anti-infection and anti-inflammatory medication delivery systems have been extensively investigated using metals such as silver and gold, as well as metal oxide NPs such as cupric oxide (CuO), SiO_2_, and TiO_2_. Metal NPs can be classified into pure metal NPs, alloy NPs, and core-shell NPs based on their composition and structure. Pure metal NPs are composed of a single metal element such as gold, silver, copper, etc. These NPs have a single crystalline structure, and their physical, chemical, and optical properties are mainly determined by the metal component. Alloy NPs are synthesized from two or more metal elements, forming an alloy structure with specific compositions, such as magnetic nanoparticles (MNPs), which possess superparamagnetic properties, enabling magnetic-targeted gene delivery under the influence of a magnetic field. Core-shell NPs consist of a metal core surrounded by a functional material shell. This core-shell structure allows for the control of surface properties, stability, and optical activity of the NPs, providing additional functionalities.

Metal NPs possess unique shapes and sizes. Their nanoscale dimensions give rise to size effects, resulting in distinct physical, chemical, and optical properties compared to macroscopic materials.^20–22^ Compared to NPs made of other materials, metal NPs exhibit unique physical and chemical properties such as light, electrical, and magnetic properties, which can be utilized in virus and bacteria detection and diagnosis.^23^ Scientists have explored the use of metal NPs in photodynamic therapy, where they harness the reactive oxygen species (ROS) generated by metal NPs under specific wavelengths of light to kill viruses.^24^ Furthermore, researchers utilize the characteristics of metal NPs to induce changes in optical signals through surface plasmon resonance (SPR), enabling the development of highly sensitive optical sensors for virus detection and diagnosis.^25^ Definitely, metal NPs can be used not only for pathogen detection but also for bioimaging and tumor immunotherapy.^26,27^

Metal NPs have been more intensively studied in the anti-infection and anti-inflammation field, especially gold nanoparticles (AuNPs) and AgNPs. AuNPs serve as vehicles for drugs and gene delivery, having excellent biocompatibility, which can be taken up by various types of cells, such as lymphocytes, macrophages, and brain micro endothelial cells.^28^ Moreover, AuNPs can not only generate non-enzymatic ROS to combat infections but also inhibit enzymes essential for the survival of pathogenic microorganisms.^29^ AgNPs are rapidly soluble and have a low potential for drug resistance due to their small size and large surface area.^30–32^ AgNPs have been demonstrated to have anti-HIV-1 activity and to prevent the interaction between CD4 and gp120, which prevents HIV-1 from invading host cells.^33–35^ However, AgNPs are cytotoxic and genotoxic due to their interactions with electron transport chain enzymes and DNA in human cells, resulting in disrupted ATP synthesis, ROS generation, and DNA damage.^36^ But when AgNPs are used to produce antimicrobial coatings on the surfaces of medical devices such as wound dressings, catheters, and implants, they not only exhibit significant antibacterial effects against common pathogenic bacteria, but also do not show cytotoxicity when used in vitro.^37^

In conclusion, research on the application of metal NPs in anti-infection treatments is continuously advancing. Among them, AuNPs and AgNPs are the most commonly studied and applied types. However, further research and clinical trials are necessary to ensure the safety and effectiveness of applying metal NPs in antiviral treatments.

### Carbon-based NPs

Another type of nanomaterial is carbon-based, which includes fullerenes, carbon nanotubes (CNTs), and graphene. These various types of carbon-based NPs have multiple potential roles and applications in the field of anti-infective and anti-inflammatory research.

Carbon-based NPs exhibit superior anti-infective effects and are commonly used as anti-infection materials. Graphene oxide, which is oxidized based on graphene, exhibits antiviral activity at non-cytotoxic concentrations,^38^ and the viral inhibition effect was also more pronounced after Ag modification.^39^ Both in vivo and in vitro viral replication can be stopped by fullerenes and their derivatives, and their amino acid derivatives have also been demonstrated to stop viral replication.^40^ Banerjee et al. reported that protoporphyrin IX-conjugated multi-walled carbon nanotubes (PPIX-MWNT) induce RNA cleavage and protein oxidation of influenza virus (IV) under visible light, resulting in virus inactivation. Furthermore, this antiviral effect is non-specific and can be used to treat all viral infections.^41^ Also, nanofilms of MWCNTs combined with gelatin and chitosan have also been shown to possess antimicrobial activity.^42^ Ramos et al. reported for the first time the anti-Leishmania activity of fullerenes, which even reduced the liver parasite burden in the Balb/c mouse model.^43^ Certainly, carbon-based NPs have shown promising therapeutic effects not only for infectious diseases but also for inflammatory conditions such as diabetes. Khalid et al. reported that bacterial cellulose-functionalized multi-walled CNTs inhibit bacteria in diabetic wounds while promoting wound healing.^44^

Based on their excellent optical and electromagnetic properties, carbon-based NPs are ideal choices for biosensors and detection platforms. By functionalizing their surfaces, carbon-based NPs can be combined with specific biomolecules to achieve high sensitivity and selectivity in detection. For example, CNTs have been used to develop continuous sensing systems for dopamine (DA) release and ascorbic acid monitoring, and with further improvements, they can even simultaneously detect baseline levels of glucose and lactate in the rat’s brain.^45^ There are extensive studies for more in-depth research and comprehensive summaries on the sensing and imaging applications of carbon-based NPs.^46–50^

Due to their high surface area-to-volume ratio and tunable chemical properties, carbon-based NPs can enhance the solubility and stability of drugs, achieve targeted delivery, and increase therapeutic efficacy while reducing side effects. Therefore, carbon-based NPs are commonly used as drug delivery systems, where drugs can be loaded onto their surfaces or internal compartments and released in a targeted manner within the body. The drug delivery applications of carbon-based NPs have been summarized in several articles.^46,51–53^ It is recommended to refer to their articles for a more in-depth understanding of the topic.

### Lipid NPs

LNPs are a common type of nanomaterial used for drug delivery and biomedical applications. The commonly used LNPs can be broadly classified into liposomes formed by phospholipids with amphiphilic properties and solid lipid nanoparticles (SLNs) typically prepared by nanoscale emulsification techniques. Common auxiliary ingredients, including surface functionalization agents, stabilizers, polyethylene glycol (PEG)-based polymers, cholesterol, are used to regulate the stability, targeting ability, and other characteristics of lipid NPs.

LNPs are composed of lipids that are biodegradable, biocompatible, inert, low toxic, and low immunogenic.^33,54–56^ They are also easily accessible and less expensive.^33,57^ The LNPs offer pharmaceuticals with smaller size, superior surface area, increased drug-carrying capacity, superior interfacial interactions, and even significantly enhanced delivery efficiency.^58–61^ In the case of hydrophobic drugs, liposomes increase their solubility and reduce their toxicity to non-specific organs.^62–64^ In addition, LNPs can achieve sustained, gradual, or stimulus-responsive drug release through various preparation methods and material selections.^65^ LNPs are highly flexible in surface modification, allowing for chemical modifications or functionalization of their outer layer using surface modifiers to impart specific properties or functions to the NPs.^66,67^ Therefore, medications based on LNPs have superior pharmacokinetic properties, higher bioavailability, lower toxicity, fewer adverse effects, and more accumulation at the target site in vivo.^68–71^

Considerations for lipid NPs include limited stability affected by storage, restricted drug loading, and challenges in controlling drug release rates due to multiple factors.^72–75^ LNPs may be designed for stimuli-triggered release, but accuracy in physiological conditions remains a challenge.^76–79^ Research and optimization are ongoing to address these limitations and enhance lipid NP performance.

Currently, there are many studies of LNPs in the anti-infection and anti-inflammation field. Among them, liposomes are more widely used than SLNs, but research in this area is still evolving and exploring. Liposomes have been used as nanocarriers for the targeted delivery of antiviral drugs and vaccines because of their high retention time but high loading capacity.^80,81^ Also, by virtue of their good biocompatibility, liposomes are compatible with tissues and cells in living organisms, reducing the likelihood of toxicity and immune reactions.^82–84^ Also, some studies have found that liposomes can neutralize inflammation or regulate and mitigate the cytokine storm against infections and their resulting inflammatory responses.^85^ With the action of liposomes, anti-inflammatory drugs were transported to macrophages, inhibiting signaling pathways involved in inflammation and thereby calming the cytokine storm.^86^ However, some studies have reported that drug-loaded liposomes can induce inflammatory responses during infection. Based on these findings, it is suggested that liposomes can enhance the effectiveness of drug therapy against infections. In addition, phospholipids may redistribute the cell surface charge, reducing the interaction between viral particles and cell surface proteoglycans, which inhibits viral entry.^87^ These are sufficient to demonstrate the superiority of lipid NPs as a platform for carrying anti-infection and anti-inflammatory drugs.

LNPs have also received extensive research in the field of mRNA vaccine delivery. Ionizable LNPs have demonstrated significant advantages in delivering mRNA vaccines, including the ability to efficiently deliver mRNA to antigen-presenting cells (APCs).^88,89^ The LNPs can also transfect neutrophils, macrophages, and dendritic cells (DCs), demonstrating that they may help transfer mRNA to a range of immune cells.^90–92^ In addition, there have been significant advancements in the research of delivering mRNA to the lungs via LNPs.^60^ An inhaled delivery lipid vector can overcome the specific cell type, mucus barrier and mucus cilia clearance system of the lung to achieve specific aggregation.^93^ Such nanotechnological platforms offer the advantages of a cell-free system, rapid production, high versatility, and a good safety profile over conventional vaccines.

In summary, lipid NPs are an important nanomaterial with a wide range of applications in drug delivery and biomedical fields. With the advancement of science, the design and optimization of lipid NPs will further enhance their performance and expand their application scope.

### Polymeric NPs

Polymeric NPs are colloidal systems that range in size from 10 to 1000 nm and have received widespread attention due to their high immunogenicity, stability, and biocompatibility.^94^ Similar to metal NPs, polymeric NPs also have a large specific surface area, which gives them good drug loading capacity.^95^ Polymeric NPs can effectively encapsulate and present antigens/drugs.^96^ Employing a ROS-sensitive polymer, Wu et al. describe the creation of polymer NPs that are intended to penetrate the brain during ischemic stroke (IS) by thrombin-stimulated diameter decrease and AMD3100-regulated precise administration.^97^ Antigen adsorption avoids exposure to harmful chemical solvents or extreme pH values during the formulation of polymeric NPs. The encapsulation also protects antigens/drugs from exposure to metabolic enzymes and harsh gastrointestinal (GI) environment in the oral route of administration.^98^ Through phagocytosis or endocytosis, polymeric NPs can increase the effectiveness of antigen uptake by APCs.^99,100^ Furthermore, polymeric NPs can enhance the efficacy of drugs by controlling the release rate and achieving targeted delivery.^101^

The creation of nanovaccines can benefit from the use of both organic polymeric NPs (like chitosan and dextran) and synthetic polymeric nanomaterials (like poly(lactic acid) (PLA) and poly(lactide-co-glycolic acid) (PLGA)), on account of polymer NPs can serve as vaccine adjuvants to enhance antigen delivery and boost immune stimulation.^102^ A polymeric Toll-like receptor (TLR) 7 agonist NP adjuvant, developed by Sun et al., improves lymph node localization and induces long-lasting immune cell stimulation and widespread immune system reactions.^103^ This method improves the antibody reactivity to a SARS-CoV-2 subunit vaccination against various newly-emerging virus strains. Natural-sourced polymeric NPs are very affordable, water-soluble, and biocompatible. Chitosan (CS) or chitosan NPs can be used as adjuvants to boost the effectiveness of inactivated Rift Valley fever virus (RVFV) vaccinations. These adjuvants cause a cell-mediated immune response that is superior to that of inactivated RVFV antigens without adjuvants.^99,104^ Compared to natural polymers, synthetic polymer NPs typically have higher reproducibility and more controllable molecular weight composition and degradation rates. The most studied synthetic NPs include poly(glycolic acid) (PGA), PLA, and PLGA. It has been demonstrated that PLA and PLGA NPs improve humoral immunity following oral and pulmonary hepatitis B immunization.^105^

However, it is crucial to ensure the biodegradability of polymeric NPs to avoid their accumulation in the body. In addition, all degradation products that may be released by polymeric NPs throughout their lifecycle must be carefully considered to prevent any toxic effects on the host.

### Protein-based NPs

Proteins and peptides are one of the main focuses of nanomedicine research and are mainly classified into animal proteins, plant proteins, and protein cages.^106–108^ Animal proteins including albumin, gelatin, collagen, milk, and silk proteins are good drug matrices. Plant proteins such as zeinolysin, wheat alginolysin, and lectins are commonly used as drug delivery carriers. Protein cages are structures derived from viruses or VLPs, which are essentially viral protein capsids without nucleic acids.^109^ Different viruses can produce viral cages of different shapes, uniform sizes, and good stability. Appropriate modification or modification of viral cages can achieve protein cages with multiple functions.^110^ In addition, ferritin/synuclein protein cages and small heat shock proteins can also be classified as protein cages.^111,112^

Protein NPs have several excellent features such as biocompatibility, low production cost, high cell binding capacity and targeting.^113^ As natural products, protein NPs have good biocompatibility, less toxicity, easy to be ingested by the body while degrading rapidly and fewer drug residues.^114^ Natural proteins are abundant and can be extracted directly, and the production methods of recombinant proteins are suitable for large-scale applications.^107^ In addition, proteins possess a variety of functional groups that can increase the amount and type of drug loading.^115^ The specific binding sites of protein NPs facilitate improved drug targeting.^115^ Different types of protein NPs each have characteristics that give them special functionality. Gelatin exhibits a rational ionic distribution with a balanced ratio of cations:anions:hydrophobic groups at 1:1:1, which makes it suitable for a wide range of pharmaceutical formulations.^116,117^ The reactive groups (arginine-lysine-glycine sequence) on gelatin are favorable for targeted treatment of infectious diseases such as acquired immune deficiency syndrome and malaria.^118^ Collagen NPs with their small size, large surface area, high absorption capacity and stable dispersion in aqueous solutions can be used as carriers for slow-release drugs, which are important in the antibacterial field.^119^ The protective effect of milk proteins is favorable for transporting some sensitive drugs and enhancing their stability.^120^ Plant proteins are mostly hydrophobic and are suitable for drug delivery of hydrophobic proteins.^121^ Lectins are resistant to hydrolytic degradation of proteins and have specific identification of intestinal glycosylation components and binding sites, which are beneficial for improved absorption of antiviral drugs.^122,123^ VLPs are a promising vaccine delivery system due to their non-infectious nature, great immunogenicity, and high biological activity.^124–127^ VLPs can also capture molecules such as proteins and nucleic acids, thereby acting as a vehicle to deliver these molecules to target cells and stimulate adaptive immunity.^128–131^

Nanoproteins have been used as important diagnostic and therapeutic agents for infectious diseases and inflammatory conditions. On the one hand, they can be used to make various biosensors to diagnose diseases, such as antibodies for detecting various viral diseases and glucose oxidase (GOx) for making glucose nanobiosensors.^132^ On the other hand, many proteins and peptides have been used in delivery of vaccines and drugs.

### A special type of NPs-Exosomes

Exosomes, as a type of extracellular vesicle, are small vesicles secreted by cells and possess important biological functions. Based on their origins, exosomes can be classified into various types, such as tumor cell-derived vesicles, immune cell-derived vesicles, and stromal cell-derived vesicles. These vesicles play crucial roles in intercellular communication,^133^ modulation of antiviral immune responses,^134^ and participation in tissue repair.^135^ The small size, modifiability, compositional diversity, and heterogeneity of exosomes make them a new class of effective nanodrugs.

The size of exosomes is usually less than 200 nm, exosomes not only contain proteins involved in many basic cellular processes, such as cell adhesion, membrane fusion, metabolism, and signal transduction, but are also capable of delivering nucleic acids, including microRNAs (miRNAs), mRNAs, DNA, and other non-coding RNAs. The diverse compositions are the basis for their high biocompatibility and wide range of applications.^136^ In addition, exosomes can be modified by genetic or cellular engineering to introduce proteins or nucleic acids, which can increase the targeting and multifunctionality of exosome-based drugs.^137,138^

Currently, exosomes are mostly used as drug carriers. As nanocarriers, exosomes possess numerous advantages. Firstly, exosomes are autologous materials, exhibiting excellent biocompatibility and stability.^139,140^ Compared with other nanomaterials, they evoke lower immune system rejection responses. Secondly, exosomes can transport various drug molecules and enhance the bioavailability and therapeutic efficacy of drugs through specific targeting and transmembrane transport.^141^ In addition, exosomes exhibit greater advantages in mRNA formulations over liposomes. They not only demonstrate superior expression and safety,^142^ but also show enhanced lung retention time and distribution.^143^ The study of exosomes would be more accurate if limitations such as the complexity of the production, purification process, and difficulties in standardization could be overcome.

With intensive research on unmodified or engineered exosomes, researchers have now constructed a variety of exosome-based biotherapeutics that can be used to treat infectious diseases and inflammatory conditions. Exosomes act as delivery vehicles for existing drug molecules, nucleic acids, and proteins. Natural or modified exosomes can also be used as immunomodulators or ROS activators for the treatment of cancer or immune-related inflammation.^144^ In addition, exosomes’ unique nucleic acids and proteins allow them to be used as biomarkers involved in the diagnosis and prognosis of infectious diseases and inflammatory conditions.^145,146^

### Other types of NPs

In addition to the aforementioned nanomaterials, combined NPs, biomimetic NPs, and QDs are also commonly used nanocarriers for drug delivery and diagnostics in infectious and non-infectious diseases. Various combined applications of NPs can supplement their shortcomings, produce synergistic effects, and make nanomaterials more developmental.^147–149^ Moreover, biomimetic nanotechnology has emerged and been used in the prevention and treatment of diseases, such as nanoenzymes and nanotoxins. Nanoenzymes are nanomaterials with enzymatic properties, characterized by high catalytic activity, stability, low cost, and scalability.^150^ It can be designed as a targeted delivery vehicle or simulate the catalytic generation of ROS, such as oxidases and peroxidases, which can simultaneously disrupt various essential biomolecules crucial for bacterial cell viability.^151–155^ Similarly, nanoenzymes can be encapsulated with antioxidants to combat oxidative stress and treat inflammatory diseases.^156–162^ Nanotoxins are NPs with membrane structures wrapping around bacterial toxins designed to reduce toxicity and increase biocompatibility.^163,164^ At present, nanotoxins have been developed as vaccines or drugs for the treatment of many diseases, such as cancer and bacterial infections.^163,164^ QDs are nanomaterials with unique optical properties that can be applied in bioimaging and diagnostics.^165^ QDs can be engineered into specific targeted probes for detecting the presence of pathogens, the expression of biomarkers,^166,167^ diagnostic imaging of neurodegenerative diseases,^168,169^ cardiovascular diseases,^170,171^ and more.

These different types of NP drug carriers have wide applications in the development of vaccines, delivery of anti-infective and anti-inflammatory drugs, and detection of pathogens and inflammation. They can improve the bioavailability, stability, and targeted delivery ability of drugs, contributing to improved anti-infective and anti-inflammatory efficacy and a balance between treatment safety and effectiveness. In addition, the unique optical and electrical properties of NPs enable their use in detecting viruses and pathogens, localizing, treating inflammation, and monitoring drug delivery in vivo. This not only improves the sensitivity and specificity of pathogen detection but also allows for diagnosis and treatment of diseases in a safe and non-invasive manner, without being limited by time or location. It should be noted that each NP drug carrier has its specific advantages and application scope, depending on the properties of the drug, delivery requirements, and treatment targets. Further research and evaluation are needed for the selection and design of specific diseases and drugs to ensure their safety and efficacy.

## Nanotechnology’s application in infectious diseases

Infections are frequently caused by viruses, bacteria, fungi, parasites, and other microbes, which constitute a serious threat to human health. This section focused on how nanotechnology is being used to treat various infectious diseases, including the development of vaccination platforms, nanocarrier delivery systems, pharmaceuticals with direct anti-infective effects, and infectious disease diagnostic methods.^172^ The first part of Table 2 summarizes clinical studies of nanotechnology for infectious diseases.

**Table 2:** Clinical studies of NPs for infectious and inflammatory diseases

Role	Conditions	Interventions	Type	Primary outcome measures	Phases	Sponsor	NCT Number	Ref
*Infectious diseases*								
Vaccine	Coronavirus; COVID-19	PepGNP-COVID19	Peptide vaccine	SAEs; AESI	Phase1; Phase2	Emergex Vaccines Holding Ltd.	NCT05633446	
	COVID-19	mRNA-1273	LNP mRNA vaccine	AEs; SAEs; MAAEs; AESIs; GMT; GMFR	Phase1; Phase2	NIAID	NCT04889209	
	COVID-19	mRNA-1273	LNP mRNA vaccine	MAAEs; NOCMCs); SAEs	Phase1	NIAID	NCT04283461	^761^
	COVID-19	mRNA-1273.351	LNP mRNA vaccine	MAAEs; NOCMCs; AESIs; SAEs; AEs	Phase1	NIAID	NCT04785144	
	COVID-19	mRNA-1273	LNP mRNA vaccine	Incidence; mean peak nasal viral load	Phase3	NIAID	NCT04811664	^762,763^
	COVID-19; HIV	mRNA-1273	LNP mRNA vaccine	NAAT; ARDS	Phase2; Phase3	COVID-19 Prevention Network	NCT05168813	
	COVID-19	HDT-301	LNP repRNA vaccine	AEs; AESI; SAE; NOCMCs	Phase1	HDT Bio	NCT05132907	
	COVID-19	HDT-301	LNP repRNA vaccine	AEs; SAEs; AESI	Phase1	SENAI CIMATEC	NCT04844268	
	COVID-19	BNT162b2	LNP mRNA vaccine	GMTs; GMR; AEs; SAEs	Phase3	BioNTech SE	NCT04816669	
	COVID 19	BNT162b2	LNP mRNA vaccine	GMT; PRNT	Phase4	The University of Hong Kong	NCT05057182	^764^
	COVID-19	PTX-COVID-19-B; Vaxzevria®	LNP mRNA vaccine	Immunogenicity response	Phase3	Everest Medicines (Singapore) Pte., Ltd.	NCT05534035	^765^
	COVID-19	PTX-COVID-19-B	LNP mRNA vaccine	NT50; PBNA; AEs; SAEs; MAAEs; AESI; PIMMC	Phase3	Everest Medicines (Singapore) Pte., Ltd.	NCT05534048	
	COVID-19	RNA MCTI CIMATEC HDT Vaccine	LNP; repRNA Vaccine	Ieutralizing antibody titers	Phase2	Azidus Brasil	NCT05542693	^766,767^
	COVID-19	QTP104	LNP repRNA vaccine	AEs; SAEs; AESI	Phase1	Quratis Inc.	NCT05876364	
	COVID-19	AS03; BNT162b2; CoV2 preS dTM [B.1.351]; mRNA-1273	LNP mRNA vaccine	GMFR; MSD; GMT	Phase1; Phase2	NIAID	NCT05289037	^768^
	COVID-19	DS-5670a DAICHIRONA	LNP mRNA vaccine	AEs; GMT; GMFR	Phase1; Phase2	Daiichi Sankyo Co. Ltd.	NCT04821674	
	COVID-19	ChulaCov19 vaccine	LNP mRNA vaccine	AEs; SAEs; GMT	Phase1; Phase2	Chulalongkorn University	NCT04566276	
	COVID-19	Bivalent Moderna; Novavax	mRNA Vaccine; self-assembled protein NPs	IgG antibodies; solicited reactions	Phase3	Murdoch Childrens Research Institute	NCT05658523	
	COVID-19	SARS-CoV-2 mRNA Vaccine	mRNA Vaccine	Primary efficacy endpoint	Phase3	Walvax Biotechnology Co., Ltd.	NCT04847102	
	COVID-19	CoronaVac; Comirnaty	Inactivated vaccine; mRNA vaccine	GMT; PRNT	Phase4	The University of Hong Kong	NCT05057169	
	COVID-19	SPFN_1B-06-PL; ALFQ	Ferritin-nanoparticle; LNP	Post-vaccination reactions	Phase1	U.S. Army Medical Research and Development Command	NCT04784767	^769,770^
	COVID-19	COVID-19 rS	Self-assembled protein NPs	AEs; SAEs; MAAEs; AESIs; GMT; GMFR	Phase1; Phase2	Novavax	NCT04368988	
	COVID-19	ChAdV68-S; SAM-LNP-S	Self-assembled protein NPs	AESIs; PIMMCs; MAAEs; NOCMCs	Phase1	NIAID	NCT04776317	^771^
	COVID-19	GBP510&AS03	Self-assembled protein NPs	AEs; SAEs; MAAEs; AESIs; GMFR	Phase1; Phase2	SK Bioscience Co., Ltd.	NCT04750343	^772^
	COVID-19	GBP510	Self-assembled protein NPs	AEs; SAEs; MAAEs; AESIs; GMT; GMFR	Phase1; Phase2	SK Bioscience Co., Ltd.	NCT04742738	^220^
	COVID-19	ICC Vaccine	Self-assembled protein NPs	systemic AEs; MAAEs; AESIs; PIMMCs; SAEs	Phase1; Phase2	Novavax	NCT04961541	^773^
	COVID-19	GBP510	Self-assembled protein NPs	GMT; GMFR	Phase2	Korea University Guro Hospital	NCT05175950	
	COVID-19	SARS-CoV-2 subunit protein recombinant vaccine	Self-assembled protein NPs	GMT	Phase2	PT Bio Farma	NCT05525208	
	COVID-19	CIC Vaccine	Self-assembled protein NPs	AEs; MAAEs; AESIs; SAEs	Phase2	Novavax	NCT05519839	^774^
	COVID-19	NVX-CoV2373	Self-assembled protein NPs	AEs; SCR; GMT	Phase2	Novavax	NCT05112848	
	COVID-19	SARS-CoV-2 rS/Matrix-M1 Adjuvant	Self-assembled protein NPs	(+) PCR-confirmed; AEs; MAAEs; MedDRA; GMTs; GMFRs	Phase2	Novavax	NCT04533399	
	COVID-19	NVX-CoV2373; NVX-CoV2601 Bivalent BA.4/5	Self-assembled protein NPs	GMTR; SRRs; NI	Phase2; Phase3	Novavax	NCT05925127	
	COVID-19	COVID-19 vaccines	Self-assembled protein NPs	IgG antibodies	Phase3	Murdoch Childrens Research Institute	NCT05387317	
	COVID-19	NVX-CoV2373; BBIBP-CorV vaccine	Self-assembled protein NPs	Utilizing ratio of IgG GMTs; MAAEs; AESIs; SAEs	Phase3	Cogna Technology Solutions LLC	NCT05249816	
	COVID-19	COVID-19 Protein Subunit Recombinant Vaccine	Self-assembled protein NPs	GMT; seroconversion rate	Phase3	PT Bio Farma	NCT05433285	
	COVID-19	Tozinameran; Elasomeran; Bivalent Pfizer; Bivalent Moderna	Self-assembled protein NPs	IgG antibodies	Phase3	Murdoch Childrens Research Institute	NCT05543356	
	COVID-19	SARS-CoV-2 rS/Matrix M1-Adjuvant	Self-assembled protein NPs	Participants with symptomatic mild; moderate; or COVID-19	Phase3	Novavax	NCT04583995	^775^
	COVID-19	GBP510 adjuvanted with AS03	Self-assembled protein NPs	GMTs	Phase3	SK Bioscience Co., Ltd.	NCT05007951	^776^
	COVID-19	GBP510 adjuvanted with AS03	Self-assembled protein NPs	GMFR	Phase3	SK Bioscience Co., Ltd.	NCT05501522	
	COVID-19	NVX-CoV2515; NVX-Cov2373; NVX-CoV2540	Self-assembled protein NPs	MN50; GMTs; SRRs; NAb	Phase3	Novavax	NCT05372588	
	COVID-19	SARS-CoV-2 rS/Matrix-M1 Adjuvant (NVX-CoV2373)	Self-assembled protein NPs	Symptomatic; (+) PCR; MAAEs	Phase3	Novavax	NCT04611802	^207^
	COVID-19; HIV	Ad26.COV2. S Vaccine; SARS-CoV-2 rS; BNT162b2	Self-assembled protein NPs	humoral immune responses	Phase2	The Aurum Institute NPC	NCT05515042	
	Influenza	H3 mRNA / LNP	LNP mRNA vaccine	AEs; CRF; SAEs; AESIs; HAI-Ab; GMTs	Phase1	Sanofi Pasteur; a Sanofi Company	NCT05829356	
	Influenza	DCVC H1 HA mRNA vaccine	LNP mRNA vaccine	AESIs; ILI; MAAEs; NOCMCs; SAEs; AEs	Phase1	NIAID	NCT05945485	
	Influenza	VRC H1ssF 3928	LNP mRNA vaccine	AESIs; NOCMCs; SAEs; MAAEs; AEs	Phase1	NIAID	NCT05755620	
	Influenza	UFluA	Hemagglutinin stabilized stem nanoparticle vaccine	AEs; SAEs; AESIs; MAAEs	Phase1	Emergent BioSolutions	NCT05155319	^777^
	Influenza	Tri-NIV with NanoFlu	Self-assembled protein NPs	AEs; MAAEs; SAEs; SNMCs; MAE; SAE; HAI; GMR	Phase1; Phase2	Novavax	NCT03293498	
	Influenza	Quad-NIV	Self-assembled protein NPs	AEs; MAEs; SAEs; SNMCs; GMT	Phase2	Novavax	NCT03658629	
	Influenza	Quad-NIV with NanoFlu	Self-assembled protein NPs	GMFR; SCR; AEs; MAAEs; SAE; SNMCs	Phase3	Novavax	NCT04120194	^212^
	RSV	RSV mRNA LNP CL-0059&0137	LNP RSV mRNA Vaccine	AEs; MAAEs; SAEs; AESIs; GMTs	Sanofi Pasteur; a Sanofi Company	Phase1; Phase2	NCT05639894	
	RSV; older adults	RSV-F Vaccine	Protein NPs	AEs; GMR; SCR; SRR	Novavax	Phase1	NCT01709019	
	RSV; Healthy volunteers	RSV-F Vaccine	Protein NPs	AEs; MAEs; SAEs; SNMCs; GMEU; GMR; SRR	Novavax	Phase1	NCT02296463	
	RSV	RSV-F Vaccine	Protein NPs	GMT; GMR; SCR; AEs	Novavax	Phase2	NCT01704365	
	RSV	RSV-F Vaccine	Protein NPs	GMEU; GMR; SRR; SCR2 and SCR4	Novavax	Phase2	NCT02593071	
	RSV	RSV-F vaccine	Protein NPs	AEs; MAEs; SAEs; medically-attended LRTI	Novavax	Phase2	NCT02247726	
	RSV	RSV-F vaccine with adjuvant	Protein NPs	RSV LRTI or tachypnea 90 days	Novavax	Phase3	NCT02624947	
	RSV	RSV-F Vaccine	Protein NPs	Serum IgG antibody titers; GMEU; GMR; GMFR; SRR	Phase2	Novavax	NCT01960686	^214^
	EBV; Mononucleosis	EBV gp350-Ferritin Vaccine	Ferritin vaccine	Local and systemic reactogenicity; SAEs; AEs	Phase1	NIAID	NCT04645147	^778–780^
	EBV; Mononucleosis; Herpesvirus	EBV gp350-Ferritin Vaccine	Ferritin vaccine	mean EBV neutralizing antibody	Phase1; Phase2	NIAID	NCT05683834	^778–780^
	EBOV GP Vaccine	Ebola	Protein NPs	AEs; SAEs; MAEs; SNMCs; GMT; GMR; SCR; SRR	Phase1	Novavax	NCT02370589	^781^
Drug delivery								
	COVID-19	Methotrexate-LDE	Lipid NPs	Lung injuries	Phase1; Phase2	Azidus Brasil	NCT04352465	
	Coronavirus; COVID-19; Inflammation	Methotrexate-LDE	Lipid NPs	Duration of hospital stay	Phase1; Phase2	University of Sao Paulo General Hospital	NCT04610567	
	COVID-19; Corona Virus	GS-5734	Inhaled lipid NPs	AEs	Phase1	NeuroActiva, Inc.	NCT04480333	
	COVID-19	VESTA respirator	Chitosan NPs	Incidence of laboratory-confirmed COVID-19	NA	University of Brasilia	NCT04490200	
	COVID-19	Intranasal ivermectin spray	Aqueous nanosuspensions	Progression of COVID-19 clinical picture	Phase2; Phase3	South Valley University	NCT04716569	^263^
	Covid19	MSC-exosomes	Exosomes	AEs	Phase1; Phase2	AVEM HealthCare	NCT04798716	
	COVID-19	MSC-exosomes	Exosomes	Cytokine profile; inflammatory biomarkers	NA	University of Ulm	NCT05191381	
	COVID-19	Hemopurifier	Exosomes	AEs	NA	Aethlon Medical Inc.	NCT04595903	
	Long COVID-19 Syndrome	UCMSC-derived exosomes	Exosomes	Cough Evaluation Test	Early-Phase1	Huazhong University of Science and Technology	NCT05808400	
	COVID-19	Stem cell Exosomes	Exosomes	Symptom remission time; serum inflammatory markers	Early-Phase1	First Affiliated Hospital of Wenzhou Medical University	NCT05787288	^782,783^
	Severe COVID-19	MSCs-derived exosomes	Exosomes	AEs; SAEs; TTIC	Phase1	Ruijin Hospital	NCT04276987	^250^
	Corona Virus; Pneumonia	CSTC-Exo	Exosomes	AEs; SAEs; TTCR	Phase1	TC Erciyes University	NCT04389385	
	COVID-19；ARDS	EV-Pure&WJ-Pure	Exosomes	AEs	Phase1	Vitti Labs; LLC	NCT05387278	
	COVID-19	EXO-CD24	Exosomes	AEs	Phase1	Tel-Aviv Sourasky Medical Center	NCT04747574	
	Corona Virus; COVID-19; SARS; ARDS	Zofin	Exosomes	AEs	Phase1; Phase2	Organicell Regenerative Medicine	NCT04384445	
	COVID-19	Zofin	Exosomes	SAEs	Phase1; Phase2	Organicell Regenerative Medicine	NCT05228899	
	COVID-19	EXO 1 &EXO 2 inhalation	Exosomes	AEs	Phase1; Phase2	State-Financed Health Facility；Samara Regional Medical Center Dinasty	NCT04491240	
	COVID-19	CAP-1002	Exosomes	Incidence of All-Cause Mortality	Phase2	Capricor Inc.	NCT04623671	
	COVID-19	CovenD24	Exosomes	SAEs; respiratory rate and SpO_2_ saturation	Phase2	Athens Medical Society	NCT04902183	
	COVID-19	EXO-CD24	Exosomes	Safety efficacy respiratory failure rate; death rate; PRO	Phase2	Eli Sprecher; MD	NCT04969172	
	COVID-19; ARDS	ExoFlo	Exosomes	60-day Mortality Rate	Phase2	Direct Biologics; LLC	NCT04493242	^784^
	COVID-19	EXO 1&EXO 2	Exosomes	AEs	Phase2	Olga Tyumina	NCT04602442	
	COVID-19	MSC-Exosome	Exosomes	Time to clinical improvement	Phase2; Phase3	Dermama Bioteknologi Laboratorium	NCT05216562	
	Antibiotic Resistant Infection	CIP-CS-PLGA-NPs	Chitosan coated PLGA NPs	Controlled release	Early-Phase1	British University In Egypt	NCT05442736	
	Cryptococcal Infections	Encochleated Amphotericin B	Lipid-crystal NPs	Tolerability of drug over 14 days	Phase1; Phase2	Matinas BioPharma Nanotechnologies, Inc.	NCT03196921	
	Bacterial Infections Oral	Chitosan	Coated PLGA NPs	Bacterial count	NA	British University In Egypt	NCT05475444	
	Carious Lesion	Titania nanoparticle reinforced bonding agent	Titania nanoparticle	Post-restorative sensitivity	NA	Pakistan Institute of Medical Sciences	NCT05744648	
	Glutathione-cyclodextrin Complex Absorption	GSH-CD	GSH-CD	GSH	Phase1	Western University of Health Sciences	NCT05926245	^423^
	Sepsis	Circulating Exosomes	Exosomes	mortality; All-cause mortality,28 days	NA	University of Kansas Medical Center	NCT04979767	
	Sepsis; Critical Illness	MSC-EXO	Exosomes	The death rate of children	NA	Children’s Hospital of Fudan University	NCT04850469	
	Drug-resistant	haMPC-Exos	Exosomes	Clinical cure rate,8 days	Phase1; Phase2	Ruijin Hospital	NCT04544215	
	Tinea	Oxiconazole nitrate SLNs loaded gel	SLNs	Clinical improvement; AEs	Phase1	Minia University	NCT03823040	
Drug								
	COVID-19	Mouthwash and nose rinse with the AgNPs	AgNPs	Incidence of SARS-CoV-2 infection	NA	Cluster de Bioeconomia de Baja California, A.C	NCT04894409	
	HIV	TLC-ART	DCNP	Cmax; Tmax	Phase1	University of Washington	NCT05850728	
	HIV	DermaVir; HAART	HIV-like particles	HIV-specific memory T cells	NA	Genetic Immunity	NCT00918840	
	HIV	DermaVir;	HIV-like particles	Primary safety endpoint	Phase2	Genetic Immunity	NCT00711230	
	HIV	DermaVir; HAART	HIV-like particles	Grade 3 Adverse Event	Phase1	Genetic Immunity	NCT00712530	
	HIV	RPV	Nanosuspensions of pure drug	Cmax; AUC (last)	Phase1	Janssen Infectious Diseases BVBA	NCT02547870	^785,786^
	HIV	RPV	Nanosuspensions of pure drug	AEs	Phase1	Janssen Research & Development, LLC	NCT01656018	^265^
	HIV	V3G CH848 Pr-NP1; 3M-052-AF	Ferritin NPs; LNP	AEs; SAEs; MAAEs; AESIs	Phase1	NIAID	NCT05903339	
	Candida Infection	Titanium dioxide NPs	Titanium dioxide NPs	Bacterial colony-forming units	NA	Cairo University	NCT03666195	
	Nosocomial Infections	AgNPS; copper NPs	Mental NPs	The inhibition zone; antibiofilm Activity	NA	Sohag University	NCT04775238	
	Caries Class Ii	Nano Care Gold	Gold & AgNPS	Marginal adaptation	Phase1	Cairo University	NCT03669224	
	Fungal Foot Infection	Whitfield; zinc oxide NPs	Zinc oxide NPs solution	KOH test	Phase4	Mahidol University	NCT05901961	
	Foot Infection Fungal	AgNPS	AgNPS	The antimicrobial activity	Phase1	Ahmed A. H. Abdellatif	NCT03752424	
	Cutaneous Leishmaniasis	Sm29 Protein	AuNPS	Cure, 90 days	Phase1; Phase2	Hospital Universitário Professor Edgard Santos	NCT06000514	
Dietary supplement	Recurrent Urinary Tract Infection	Magnalife	Nanotechnology Structured water	Urinalysis by GUE	NA	University of Sulaimani	NCT04306731	
Sensors	Tuberculosis	Nanodisk-MS assay	Silicon NPs	Correlation, Sensitivity, specificity, positive predictive value, and negative predictive value	NA	Chinese University of Hong Kong	NCT03271567	^430^
Biomarker	Sepsis With MOD	NTA double markers	Fluorescent NPs	Ubiquitination-autophagy-apoptosis biomarkers	NA	Taipei Tzu Chi Hospital, Buddhist Tzu Chi Medical Foundation	NCT03222986	
	Sepsis complicated with ARDS	Diagnostic test	Exosomes	Differential miRNAs	NA	Tianjin Nankai Hospital	NCT05476029	
	Sepsis	Antibiotics	Exosomes	Amount of dendritic cell-derived exosomes	NA	Jinling Hospital, China	NCT02957279	
*Inflammatory diseases*								
Drug delivery	Rheumatoid Arthritis	Nanoparticulated rebamipide	LNEs	Change in the Clinical improvement of oral ulcers	Phase3	Cairo University	NCT04649697	
	Atherosclerosis; coronary artery disease; inflammation	Methotrexate-LDE	LDL Like NPs	LAPV coronary	Phase2; Phase3	University of Sao Paulo General Hospital	NCT04616872	
	Coronary artery aisease; atherosclerosis; inflammation	LDE-Paclitaxel	LDL Like NPs	LAPV coronary	Phase2; Phase3	University of Sao Paulo General Hospital	NCT04148833	
	Atherosclerosis	Iron-bearing NPs	Iron-bearing NPs	TAV; QCA; IVUS	NA	Ural State Medical University	NCT01270139	
	Coronary artery disease; atherosclerosis	Iron-bearing NPs	Iron-bearing NPs	TAV; IVUS	Phase1	Ural State Medical University	NCT01436123	
	Painful diabetic neuropathy	0.75% capsaicin nanoparticle cream	SLNs	Pain relief	Phase2; Phase3	Mahidol University	NCT01125215	
	PreDiabetes	Zein nanocapsules	Zein NPs	Change in Fructosamine	NA	Clinica Universidad de Navarra, Universidad de Navarra	NCT05560412	
	Chronic diabetic foot ulcer	Stem cell product	Chitosan NPs	Complete healing, full epithelization of chronic diabetic foot ulcer, 6 months	Phase1	Assiut University	NCT03259217	
	Plaque Psoriasis	SOR007 Ointment	LNEs	Change in the thickness of the ELB, 12 days	Phase1	DFB Soria, LLC	NCT03004339	
	Dementia; alzheimer Disease	APH-1105	LNEs	ADAS-Cog	Phase2	Aphios	NCT03806478	^787^
	Tubular breast cancer; inflammatory breast cancer	Nab-Paclitaxel (Abraxane®)	Protein-NPs	Pathological complete Response (pCR=ypT0 ypN0) rates	Phase3	German Breast Group	NCT01583426	
	Inflammatory breast cancer	Carboplatin; paclitaxel albumin-stabilized nanoparticle formulation	Protein-NPs	Pathological complete response (pCR=ypT0 ypN0) rates	Phase2	City of Hope Medical Center	NCT01525966	^788^
	Breast cancer	capecitabine; paclitaxel albumin-stabilized nanoparticle formulation; neoadjuvant therapy	Protein-NPs	Pathological complete response rate	Phase2; Phase3	Medstar Health Research Institute	NCT00397761	
	Breast cancer; HER2-negative breast cancer	Carboplatin; paclitaxel albumin-stabilized nanoparticle formulation	Protein-NPs	Progression free survival	Phase2	University of California, Irvine	NCT00618657	
	Irritable bowel disease	Ginger exosomes	Exosomes	Change in inflammation on Colonoscopy	NA	University of Louisville	NCT04879810	
	Ankylosing Spondylitis	Nanocurcumin	Nanomicelles spherical water	BASDI	Phase2	Tabriz University of Medical Sciences	NCT03140657	
Drug	Chronic rhinosinusitis	Colloidal AgNPS	AgNPS	SNOT-22	Phase1	Washington University School of Medicine	NCT03243201	
	Knee arthritis; rheumatoid arthritis	Gold factor	AuNPs	KOOS	NA	4Life Research, LLC	NCT05347602	
	Type 1 Diabetes	C19-A3 GNP	Peptide-AuNPS	General safety and induction of hypersensitivity	Phase1	Cardiff University	NCT02837094	
	Inflammatory disease	Inhaled AgNPS	AgNPS	Ex vivo inflammatory response	NA	NIEHS	NCT02408874	
	Crohn’s Disease	Placenal MSC derived exosomes	Exosomes	Safety of injected exosomes	Phase1; Phase2	Tehran University of Medical Sciences	NCT05499156	
	Multiple Sclerosis	Gold nanocrystals	AuNPs	CNS metabolic changes	Phase2	Clene Nanomedicine	NCT03993171	
Dietary supplement	Type2 diabetes	Magnalife	Nanotechnology structured water	HbA1c	NA	ALI KAMAL M. SAMI	NCT04082351	
Sensors								
	Multiple sclerosis	NA-NOSE artificial olfactory system	Carbon nanotubes and AuNPS	Identification of volatile compounds in exhaled breath	complete	Carmel Medical Center	NCT01465087	
	Multiple sclerosis	NA-NOSE artificial olfactory system	Carbon nanotubes and AuNPS	Successful discrimination	NA	Carmel Medical Center	NCT01206023	
	Metabolic syndrome; diabetes	Breath analysis and blood analysis	nano-chemical sensors	Development of diabetes or complication	Phase1	Rambam Health Care Campus	NCT01268813	
	Neurodegenerative diseases (AD, PD)	™NA-NOSE	Carbon nanotubes and AuNPS	NA	NA	Rambam Health Care Campus	NCT01291550	^594^
Imaging marker of MRI								
	Multiple sclerosis	USPIO nanoparticle	USPIO nanoparticle	Signal change on T1-weighted and 3D UTE MRI brain	Early-Phase1	University of Utah	NCT05357833	
	Multiple sclerosis	Ferumoxytol	Iron oxide NPs	Brain signal intensity	Phase1	NINDS	NCT02511028	
	Myocardial infarction; inflammation	Cardiac magnetic resonance imaging	Iron-bearing NPs	Cardiac MRI signal intensity	NA	University of Edinburgh	NCT01127113	
	Diabetes mellitus, type 1	Ferumoxtran-10	Iron oxide NPs	Changes in the pancreas associated with autoimmune diabetes	NA	Joslin Diabetes Center	NCT00585936	
Biomarker	Pleomorphic adenoma of salivary glands	CD24-Gold Nanocomposite	AuNPS	Non-conjugated CD24	NA	Amina Fouad Farag	NCT04907422	^789^
	Hemodynamic instability; autophagy	Hemodynamic parameters	Exosomes	Change of hemodynamic parameters	NA	Taipei Tzu Chi Hospital, Buddhist Tzu Chi Medical Foundation	NCT03267160	

Data available as of 15 September 2023. Data obtained from https://clinicaltrials.gov/. The first column highlights nanoparticles’ roles in diagnosing and treating diseases, such as their applications as vaccines, delivery vehicles, drugs, sensors, and diagnostic markers while the fourth column lists the various types of NPs

*COVID-19* Corona Virus Disease 2019, *CSTC-Exo* COVID-19 Specific T Cell derived exosomes, *AEs* adverse reaction, *SAE* severe adverse reaction, *TTIC* time to clinical improvement, *LNP* lipid nanoparticle, *NA* not applicable, *TTCR* time to clinical recovery, *PRO* patient-reported outcome measure score, *rep RNA* representations of RNA sequences, *SF-36* the 36 item Short Form Health Survey, *GMT* geometric mean titer, *GMTR* geometric mean titer ratio, *GMFR* geometric mean fold rise, *SRRs* seroresponse rates, *NI* non-inferiorto, *NIAID* National Institute of Allergy and Infectious Diseases, *PRNT* plaque reduction neutralization test, *AESIs* adverse events of special interest, *PIMMCs* potentially immune-mediated medical conditions, *MAAEs* medically attended adverse events, *NOCMCs* new onset chronic medical conditions, *GMEU* geometric mean EU, *RBD* receptor-binding domain, *GMR* geometric mean ratio, *SRR* seroresponse rate, *RSV* Respiratory Syncytial Virus, *qNIV* quadrivalent hemagglutinin (HA) nanoparticle influenza vaccine, *CIC vaccine* in-clinic mix of various doses of qNIV, SARS-CoV-2 rS, and 50 μg Matrix-M1 Adjuvant, *ICC Vaccine* qNIV and SARS-CoV-2 rS nanoparticle combination vaccine with Matrix-M1 adjuvant, *SCR2 and SCR4* proportion of subjects with two 2- and 4-fold seroconversion rates, *MedDRA* Medical Dictionary for Regulatory Activities, *NINDS* National Institute of Neurological Disorders and Stroke, *ALFQ* army liposomal formulation QS21, *AgNPs* silver nanoparticles, *pNT50* geometric mean neutralizing antibody titers against D614G pseudovirus strain, *MN50* inhibitory concentration of 50%, *LRTI* medically-attended RSV lower respiratory tract infection, *MSD* Multiplex Meso Scale Discovery; *ILI* influenza like illnesses, *EBV* Epstein-Barr Virus, *DLCD* diffusing capacity of the lungs for carbon monoxide, *CRF* case report form, *HAI-Ab* hemagglutination inhibition HAI antibody Ab, *PCR* Polymerase Chain Reaction, *NT50* the 50% neutralizing antibody titers, *PBNA* pseudovirusbased neutralization assay, *IgG* Immunoglobulin G, SARS CoV-2 spike RBD protein-specific binding antibody bAb IgG and SARS CoV-2-specific neutralizing antibody nAb, *Cmax* peak TLC-101 drug substance concentrations, *AUC* area under the plasma concentration, *MSCs* mesenchymal stem cells, *UCMSCs* umbilical cord mesenchymal stem cells, *PLGA* poly-lactic-co-glycolic acid, *SNMC* significant new medical condition, *Tmax* time to maximum TLC-101 concentration, *NAAT* nucleic acid amplification testing, *MOD* multiple organ dysfunction, *NTA* nanoparticle tracking analysis, *ARDS* Acute Respiratory Distress Syndrome, *HIV* Human Immunodeficiency Virus, *RPV* Rilpivirine, *MTX-LDE* lipid NPs carried methotrexate, *DCNP* drug combination nanoparticle, *LNEs* lipid nanoemulsions, *ELB* change in the thickness of the echolucent band, *SNOT-22* Sino-Nasal Outcome Test, *KOOS* knee injury and osteoarthritis outcome score; *LAPV* low attenuation plaque volume coronary; *LDL* low-density lipoprotein; *SLNs* solid lipid nanoparticles, *TAV* total atheroma volume, *QCA* quantitative coronary angiography, *CD24* cluster of differentiation 24, *IVUS* intravascular ultrasound, *NA-NOSE* nanoparticle nose ADAS-Cog, the Alzheimer’s Disease assessment scale-cognitive subscale test, *ELB* echolucent band, *BASDI* assessments of ankylosing spondylitis signs and symptoms, *USPIO* ultrasmall superparamagnetic iron oxide, *GSH-CD* glutathione–cyclodextrin nanoparticle complex

### NPs in viral infection

Since the beginning of the 21st century, there have been several global pandemics caused by viral infections, including Severe Acute Respiratory Syndrome Coronavirus (SARS-CoV) in 2003,^173^ H1N1 influenza in 2009,^174^ Middle East Respiratory Syndrome Coronavirus (MERS-CoV) in 2012,^175^ Ebola virus in West Africa from 2013 to 2016,^176^ Zika virus in 2015,^177^ and the SARS-CoV-2 pandemic in 2020.^178^ These outbreaks have resulted in significant morbidity and mortality, particularly the COVID-19 pandemic, which has had profound and devastating effects on individuals and societies worldwide.^179^ In the past few decades, numerous effective vaccines have been developed to control the spread of viruses such as smallpox, polio, measles, rabies, rubella, and tetanus globally or in specific regions.^180^ Traditional vaccines often produce low titers of neutralizing antibodies and may struggle to combat mutant pathogens. Previous treatments for viral infections have often been ineffective and associated with significant adverse reactions.^181^ Detection methods for pathogens have also been time-consuming, labor-intensive, and lacking in sensitivity and accuracy.^182^ The development of nanotechnology improves traditional methods for the prevention, detection, and treatment of infectious diseases.^183^

#### The application of NPs-based vaccine for virus

**Fig. 2:**
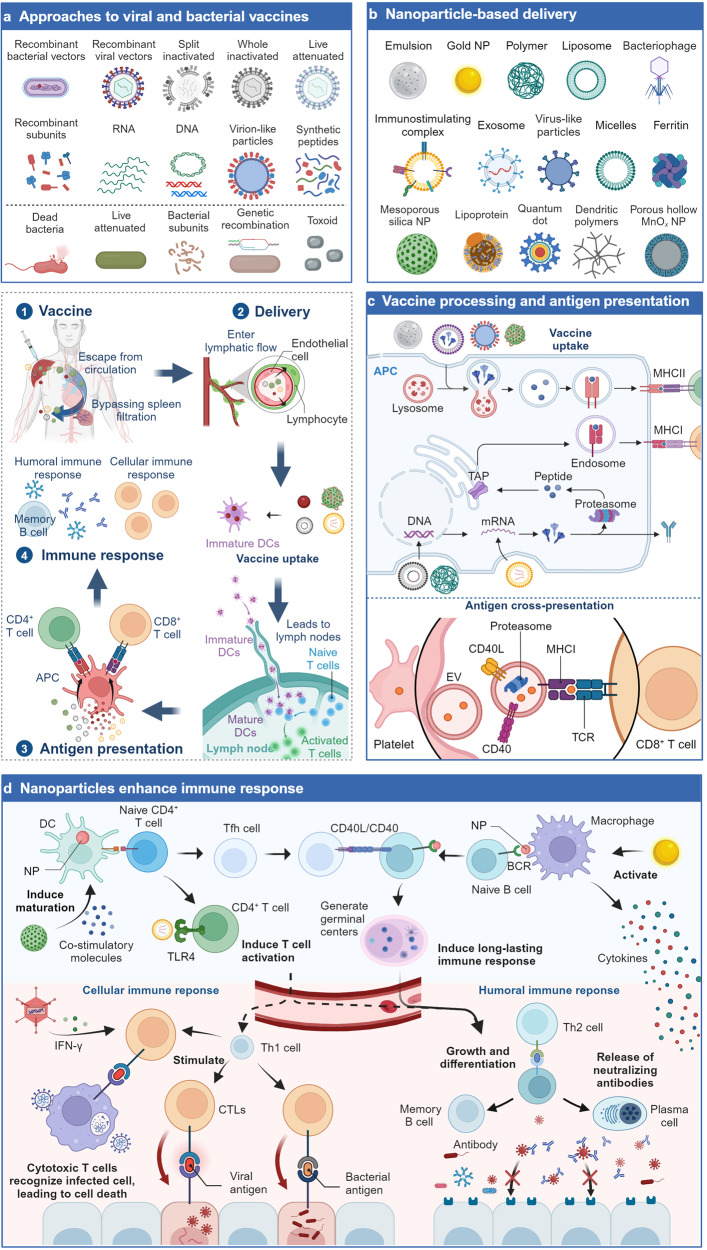
The application of NPs-based vaccines for pathogen prevention. After vaccination, due to the small size of the NP carrier, the nano-vaccines are more likely to escape from the bloodstream, bypass splenic filtration, enter the lymphatic flow, and then be absorbed by immature DCs, together with DCs, enter the lymph nodes, and initiate a series of immune reactions. **a** Types and methods for the production of viral and antibacterial vaccines. **b** Various nanomaterials used for antigen delivery. **c** Mechanisms of NP entry into cells and antigen presentation. **d** Mechanisms by which NPs enhance immune responses. NPs have diverse stimulating effects on the immune system, including inducing the production of co-stimulatory molecules to induce DCs maturation;^795,796^ promoting strong T cell activation;^276^ facilitating germinal center formation to induce long-lasting effective immune responses;^797^ and stimulating macrophages to produce cytokines to enhance immune responses.^798^ ①–④. The immune process of NP-based vaccines. TAP: transporter associated with antigen processing; Tfh: T follicular helper; Th2: T helper-2 cell; CTLs: cytotoxic T lymphocytes

Nanotechnology has been applied to the development of vaccines as adjuvants and delivery vehicles to overcome the shortcomings of traditional vaccines, such as long development time, low immunogenicity, and antibody dependence. There have been many comprehensive reviews published on the application of nanomaterials as adjuvants,^184^ so we didn’t repeat here again. This section primarily focused on the application of NPs as vaccine delivery systems, with an emphasis on the most promising delivery platforms, including LNPs, self-assembled protein-based NPs, and exosomes.^185^ These nanotechnological strategies make immunizations more effective by improving vaccine stability, providing precise antigen presentation, and enhancing immune stimulation (Fig. 2).^185–188^

##### LNP-mediated vaccines

The efficacy of nucleic acid-based vaccines depends mainly on the delivery of RNA or DNA molecules that express target-encoded antigens to trigger specific and strong immune responses in target immune cells.^189^ DNA vaccines have significant potential for the management of infectious diseases since they are easy, stable, and affordable to mass produce.^189–191^ mRNA vaccines have high antigen expression and quick clearance rates by directly expressing antigens in the cytoplasm without crossing the nuclear membrane.^192,193^ Nanotechnology-based delivery of plasmid DNA (pDNA) or mRNA molecules allows for the creation of precisely targeted nucleic acid vaccines. LNPs, as the delivery system for mRNA vaccines, can overcome the problem of naked mRNA transfection by stabilizing and successfully delivering it into cells.^194^ In phase I clinical trials, an mRNA vaccine that encodes the SARS-CoV-2 spike-in protein was reported to lower disease incidence, and viral replication was not seen in the lungs of rhesus monkeys exposed to large doses of the virus. The approved COVID-19 mRNA vaccines from Moderna and Pfizer/BioNTech are nanovaccines made from a cationic polymer/lipid complexed with negatively charged nucleic acids, which helps protect the mRNA from immune recognition and degradation.^195,196^ More importantly, the nano formulation may be effective for all current mutant strains including the Omicron variant.^197^ Recently, LNPs have been used to deliver the mRNA encoding SARS-CoV-2 S protein with incorporated ESCRT-I recruitment motif, enabling ESCRT-mediated secretion of viral spike protein VLPs from the cells. These VLPs displayed native, membrane-bound spike trimers on their surface, resulting in higher levels of neutralizing antibody titers 10 to 100-fold compared to soluble spike or commercial mRNA vaccines, and eliciting cellular immunity not achieved with mRNA vaccines alone. Notably, this VLP platform can be adapted to other viral antigens or mRNA cargoes, providing a promising direction for new vaccine development.^198^ Over the past few years, a number of mRNA vaccines against COVID-19 have been clinically studied and approved for use, the most representative lineage being mRNA-1273 and BNT162b2. Several clinical studies have demonstrated that mRNA-1273 provides strong protection (94.1%) in various age groups for more than 6 months (NCT04889209).^199^ However, despite the significant successful application of LNP-mediated mRNA in the COVID-19 vaccine, one limitation is that their stability requires freezing storage.^200^ For example, the vaccines developed by Moderna and BioNTech/Pfizer need to be stored at temperatures of −15 to −25 °C and −60 to −90 °C, respectively.^200^

In addition to COVID-19 vaccines, LNPs have potential in delivering mRNA of other viruses, enabling the development and application of vaccines for various viral diseases such as influenza, respiratory syncytial virus (RSV) and EBV. For instance, there have been reports on the design of an mRNA vaccine encapsulated in LNPs that expresses a variant of the RSV F protein. This vaccine successfully encoded multiple forms of RSV F protein in animal models and exhibited immunogenicity, providing protection against RSV infection.^201^ An LNP-mediated HIV-1 mRNA vaccine (gag mRNA/LNP) effectively enhanced the humoral and cellular responses previously induced by the DNA vaccine as a heterologous prime-boost regimen targeting monkeys.^202^ Furthermore, Peng et al. reported an effective LNP-mRNA vaccine targeting multiple pathogenic coronaviruses.^203^ These researches highlight the potential of LNPs in the development and application of vaccines for viral diseases. Three influenza mRNA vaccines are already in Phase I clinical studies, H3 mRNA/LNP, DCVC H1 hemagglutinin (HA) mRNA vaccine and VRC H1ssF 3928 (NCT05829356, NCT05945485, NCT05755620), and an RSV vaccine, RSV mRNA LNP CL-0059&0137 is in Phase II clinical trials (NCT05639894).

##### Self-assembled protein-based NP vaccines

Common self-assembling protein NPs, including VLPs, ferritin, and viral capsid proteins, have broad prospects in vaccine research and applications, demonstrating advantages in enhancing immune stimulation, antigen presentation, and physical stability.^204,205^ A recombinant SARS-CoV-2 spike protein vaccine developed by Novavax (NVX-CoV2373) produces full-length spike proteins that spontaneously form native trimeric conformations due to beneficial point mutations. This authorized vaccine exhibited robust immunogenicity and protection in baboon and mouse models, as well as demonstrated safety and efficacy in clinical trials (NCT04368988, NCT04611802).^206,207^ In another vaccine, an engineered protein combining the receptor-binding domain (RBD) domain of the SARS-CoV-2 spike protein with an HR motif self-assembles into a trimeric structure to mimic its natural conformation. In mouse and rhesus macaque models, this vaccine induced potent neutralizing antibody responses against both wildtype and variant SARS-CoV-2 strains, which led to its emergent approval in China.^208^ The VLPs that are made by the self-assembly of viral structural proteins have also been successfully applied in vaccines for various viruses, including HBV vaccines,^209^ HPV vaccines,^210^ and IV vaccines.^211^ A phase III clinical study of Quad-NIV with NanoFlu demonstrated that the qNIV vaccine was no less protective than the quadrivalent inactivated influenza vaccine (IIV4) in the elderly (NCT04120194).^212^ An anthrax vaccine used the coat protein of tobacco mosaic virus to deliver protective antigenic peptides of Bacillus anthracis.^213^ In addition, Novavax’s RSV vaccine has been shown to be well tolerated in clinical studies, with no adverse effects and a 52% reduction in infection rates in subjects overall (*p* = 0.009 overall) (NCT01960686).^214^

In addition to VLPs, other self-assembling proteins such as ferritin can also present antigens and stimulate immune responses. The spherical protein complex of ferritin forms a stable central cavity, which can be used to encapsulate target antigens and display them on the surface of ferritin. A SARS-CoV-2 vaccine made by conjugating the RBD of the viral spike protein to ferritin showed a higher affinity for the ACE2 receptor and neutralizing antibody CB6.^215^ Similarly, the safety and immunogenicity of a ferritin-based H2 influenza vaccine have been reported in a phase I trial, showing a safe, well tolerated and immunogenic potent in healthy adults.^216^

In addition, there are also proteins and peptides that have been designed as nanocarriers for viral antigens. For instance, a dengue virus E glycoprotein vaccine has been designed based on a polymeric IgG scaffold.^217^ Moreover, with the tremendous development of computational science, scientists can design ideal NPs based on experimental needs. A nanocarrier can display two different antigens by synthesizing two orthogonal reactive split proteins through the formation of heteropeptide bonds.^218^ A designed self-assembling protein NP I53-50 platform can display trimeric SARS-CoV-2 spike proteins on their surface, which elicited potent neutralizing antibody responses.^219,220^ Currently, NP vaccines based on self-assembling proteins against Lassa virus, HIV, HCV, and East Coast fever (ECF) virus have all shown good ability to induce neutralizing antibodies.^221–224^ These studies suggest that through antigen presentation by self-assembling protein vaccines, it is possible to mimic the structure and epitopes of pathogens, thereby activating the immune system to generate an immune response specifically targeting the desired antigens.

##### Exosomes-based vaccine

Exosomes, as cell-secreted products, have stronger capabilities in delivering vaccines without any side effects.^225^ There have been numerous studies utilizing exosomes to load with RNA or proteins for the COVID-19 vaccines. It has been discovered that the delivery capacity of exosomes is superior to LNPs, both in nucleic acids encoding antigens and protein immunogens.^142^ Another advantage of exosomes is their excellent affinity for target tissues. Exosomes derived from lung spheroid cells have excellent lung affinity compared to liposomes, enhancing the retention of the RBD in the mucosal lining of the respiratory tract and lung parenchyma.^143^ An inhalable COVID-19 vaccine that loaded recombinant SARS-CoV-2 RBD in lung-derived exosomes has a longer residence time in the respiratory tract and lung tissues after inhalation through nebulization.^226^ Furthermore, exosome-based vaccines have stronger immunogenicity due to their natural or immune-enhancing effects or their immune-modulating cargo, such as cytokines, nucleic acids, and lipids. Compared to Pfizer and Moderna’s mRNA vaccines and Oxford-AstraZeneca’s adenovirus vaccine, exosome-based vaccines demonstrate stronger immunogenicity, better stability, and easier storage.^226^ These benefits are attributed to the endogenous and natural homologous targeting ability of exosomes, demonstrating the superiority of exosomes in the field of viral vaccines.

Exosomes have been widely used in developing the multi-valent vaccine for SARS-CoV-2. It was found that exosomes loaded with two functional mRNAs induced long-term cellular and humoral immune responses against the spike protein and the nucleocapsid protein even after repeated injections. In mice experiments, this vaccine induced systemic humoral immune responses, including RBD-specific IgG antibodies and mucosal IgA responses in the lungs of mice. In addition, the vaccine activated CD4^+^ and CD8^+^ T cells with a Th1 cell cytokine expression profile, inducing a Th1-biased immune response and clearance of simulated SARS-CoV-2. Exosomes derived from milk have been used in an oral mRNA vaccine encoding the SARS-CoV-2 RBD.^227^ This vaccine successfully secreted RBD peptide in 293 cells and stimulated the production of neutralizing antibodies targeting RBD in mice. Furthermore, multi-valent COVID-19 vaccines containing spike proteins and nucleocapsid proteins of different SARS-CoV-2 strains have also developed based on exosomes, aiming to enhance the protective effects of the vaccines through combination strategies.^228^ In independent animal models, this vaccine induced potent and persistent neutralizing antibody responses at low doses and elicited strong T cell immune responses without the need for adjuvants.

Definitely, exosomes can also be used to develop other virus vaccines, such as HIV, HBV, HCV, IV, and rabies viruses. A targeted T-cell vaccine for HIV has developed with exosomes (Gag-Texo), which induced Gag-specific therapeutic immunity in a chronic adenovirus infection model.^229^ Exosomes derived from human monocyte cell lines hold promise as adjuvants for recombinant HBV vaccines. These exosomes could induce Th1 immune responses against HBsAg, leading to increased levels of IFN-γ in mice and promoting cellular immunity.^230^ Similarly, exosomes derived from umbilical cord mesenchymal stem cells (uMSC-Exo) can carry miRNAs to inhibit hepatitis C virus replication.^231^ Notably, outer membrane vesicles (OMV) derived from Gram-negative bacterium Burkholderia thailandensis were employed to express and package vaccine antigens derived from influenza A virus (IAV), inducing antigen-specific immune and antibody responses in mucosal tissues and systemically.^232^ Moreover, exosomes enhance the resistance of MRC-5 cells to rabies virus infection by delivering miRNA-423-5p between cells. Exosome-delivered miRNA-423-5p counteracts the inhibitory effect of cytokine signaling inhibitor 3 on type I IFN signaling, resulting in feedback inhibition of RABV replication.^233^ Overall, exosome-based viral vaccines are a promising new strategy to provide innovative immune defense against viral infections.

#### NPs in antiviral therapy

**Fig. 3:**
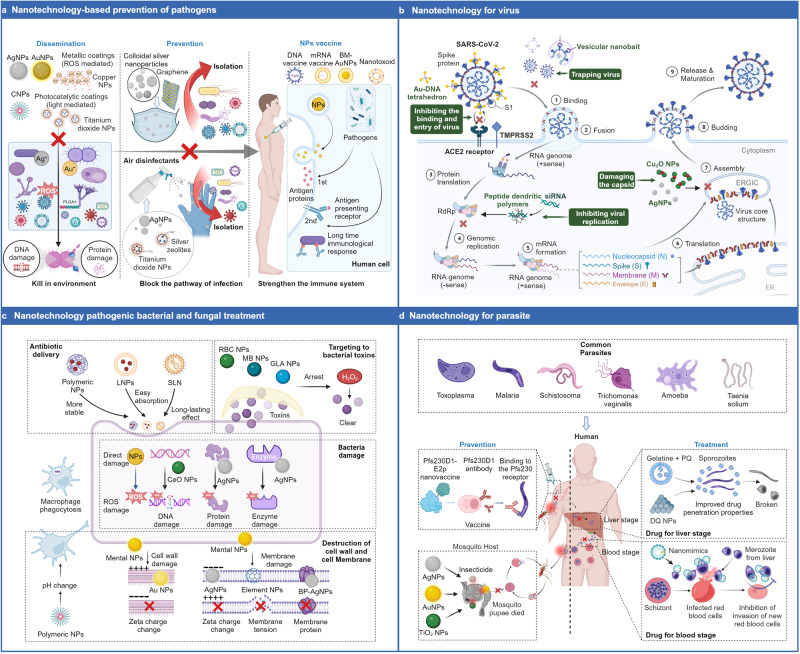
Applications of nanotechnology in the treatment of bacteria, fungal, viruses, and parasites. **a** Nanotechnology in the prevention of pathogenic bacterial infections, which include killing bacteria in the environment, blocking transmission routes and vaccination: AgNPs can release silver ions in the environment, generating ROS that damage the DNA and proteins of bacteria;^358^ AgNPs can also be applied to masks and disinfectants to prevent the invasion of pathogens and block infection pathways;^799^ Nanovaccines can enhance human immunity to improve resistance to infection. **b** Nanotechnology strategies against viruses. Taking the example of the coronavirus, nanotechnology can intervene in various processes of virus proliferation, such as capturing viruses outside nanocages, binding to spike proteins with nanocomplexes, inhibiting the binding and entry of SARS-CoV-2 into host cells. Similarly, targeting the RdRp complex can silence SARS-CoV-2 and inhibit its genome replication. AgNPs and other nanomaterials can also damage the viral envelope and disrupt the viral capsid. **c** Nanotechnology in antibacterial therapy. The antibacterial mechanism includes four aspects: the delivery of antibiotics,^390^ targeting bacterial toxins, damaging bacterial cell walls and membranes,^402^ and destroying bacterial DNA, proteins, and enzymes.^417^ **d** Nanotechnology applications against parasites. A variety of NPs targeting the parasite’s growth cycle have been used in the prevention and treatment of malaria: killing Plasmodium larvae in the environment and blocking the transmission pathway; generating antibodies to Plasmodium by nano-vaccination;^438^ and destroying Plasmodium in the liver and blood stages.^437^ RdRp: RNA-dependent RNA polymerase

Nanotechnology with precise control over the properties and structures of nanomaterials holds tremendous potential in the field of antiviral applications. The application of nanotechnology in the antiviral field, including the efficient delivery of antiviral drugs, the blocking of viral infections, and the activation of immune responses, offers new strategies and approaches, bringing renewed hope for infectious disease treatment and prevention (Fig. 3b).

##### Delivery of antiviral drugs

NPs can serve as delivery systems to effectively transport antiviral drugs to targeted cells or tissues, improving their targeting, stability, and bioavailability. Firstly, the antiviral drugs are encapsulated or adsorbed into the interior or surface of NPs, which protects the drugs from degradation factors in the external environment and enhances their stability. Due to their nanoscale size and unique surface properties, NPs can avoid excessive clearance by the immune system and exhibit prolonged circulation capability, increasing drug bioavailability and therapeutic efficacy.^234–236^ After reaching the target cells or tissues, NPs interact with the cell surface and promote their internalization through binding to specific receptors or the action of active targeting ligands.^186,237–241^ Once internalized, the NPs will release the encapsulated antiviral drugs by NP dissolution, receptor-mediated delivery, or stimuli-responsive release in response to the internal environment.^242–247^ In addition, the delivery system of NPs can achieve combination delivery of multiple antiviral drugs, combining different types of antiviral drugs together to enhance efficacy or combat drug resistance.^248^

Exosomes can effectively deliver a wide range of drug molecules such as nucleic acids, proteins, small molecules, and gene therapeutic agents, achieving high targeting and better therapeutic efficacy of antiviral drugs.

Protein drugs, such as membrane proteins, antigens, antibodies, etc., face challenges in maintaining their activity and extending their half-life when used for disease treatment. Due to the similar phospholipid bilayer structure to cell membranes, utilizing exosomes as delivery vehicles for membrane proteins can maintain their stability and achieve effective delivery. Exosomes enriched with an immune checkpoint modulator called CD24 (EXO-CD24) deliver the protein CD24 into the body through vesicular exosomes to regulate cytokine storms and combat COVID-19. In vitro and in vivo experiments have confirmed the safety and efficacy of EXO-CD24, with no drug-related adverse events reported even in a phase Ib/IIa clinical study.^249^ Furthermore, EXO-CD24 effectively reduces inflammatory markers and cytokines/chemokines in COVID-19 patients, establishing EXO-CD24 as a potential therapeutic strategy for inhibiting excessive lung inflammation in COVID-19 patients. Notably, several exosome-based drugs for the treatment of COVID-19 have entered clinical studies (NCT04798716, NCT04595903, NCT05787288). Amongst them, haMSC-Exos was well tolerated in the treatment of COVID-19 severe disease, with a significant remission of lung lesions after 7 days (NCT04276987).^250^ Zou et al. designed a method using exosomes to deliver IFN-induced transmembrane protein 3 (IFITM3) to fetuses for treating ZIKV infection.^251^ In their study, exosomes effectively transported IFITM3 across the placental barrier into late-stage fetal cells or lysosomes. The results showed that exosomes containing IFITM3 inhibited ZIKV in the fetus, significantly reducing viral viremia in the major organs of the fetus.

Nucleic acid drugs, especially miRNAs and small interfering RNA (siRNAs), are considered to have great potential for antiviral treatment. However, these types of drugs suffer from poor biological stability, easy degradation, and the potential to trigger immune reactions in the body, which limits their application. Using exosomes as delivery vehicles for nucleic acid drugs can overcome the aforementioned problems and facilitate the clinical application of these drugs. Exosomes delivered mRNA encoding ZFP-362, a zinc finger protein targeting the HIV-1 promoter and the active domain of DNA methyltransferase 3A, induce long-term stable HIV-1 epigenetic suppression, suppressing the HIV levels in the bone marrow, spleen, and brain of mice.^252^ A clinical study showed that LNP BMS-986263 could deliver siRNAs that degrade HSP47 mRNA, effectively treating pulmonary fibrosis caused by HCV infection (NCT03420768).^253^ Teng et al. reported that exosomes (exosomes^Nsp12Nsp13^) released by lung epithelial cells exposed to the replicase Nsp12 and helicase Nsp13 of SARS-CoV-2 can activate nuclear factor κB (NF-κB), subsequently inducing a series of inflammatory cytokines, including tumor necrosis factor (TNF)-α, interleukin (IL)-6, and IL-1β, and leading to apoptosis of lung epithelial cells. However, they found that ginger exosome-like nanoparticles (GELNs) carrying miRNA could inhibit the activation of NF-κB and apoptosis of lung epithelial cells mediated by exosomes^Nsp12Nsp13^, thereby treating exosomes^Nsp12Nsp13^-mediated pulmonary inflammation. Furthermore, GELNs can also inhibit cellular pathological effects induced by SARS-CoV-2, revealing the potential of GELNs as a therapeutic agent for treating SARS-CoV-2.^254^ Zhang et al. designed an antiviral therapeutic system capable of crossing the placental barrier and blood brain barrier (BBB), which involves extracellular vesicles encapsulating ZIKV-specific siRNA.^255^ They found that this system protected pregnant AG6 mice from vertical transmission of ZIKV infection and could cross the placental barrier and BBB to inhibit ZIKV infection in the fetal brain. These examples demonstrate that exosomes are promising for a wide range of applications in the delivery of antiviral drugs.

##### Blocking viral infections

NPs can interfere with the binding between viruses and host cell receptors by introducing specific ligands or antibodies through surface functionalization or modification, thereby blocking the process of viral entry into host cells.

NPs can utilize their surface binding sites to interact with target molecules to inactivate viruses. Metal NPs can interact with viral surface proteins through Kazimir interactions, van der Waals forces and disulfide bonds.^256,257^ For example, AgNPs and AuNPs can cleave the disulfide bonds on the sulfhydryl groups of viral surface proteins, thereby preventing viral entry into cells.^258,259^ Cagno et al. created antiviral NPs with long, adaptable linkers that mimicked heparan sulfate proteoglycans, enabling efficient viral connection with a binding which they simulatively intended to be powerful and multivalent to the virus ligands repeating components, producing forces (190 pN) that ultimately result in permanent viral deformation.^260,261^ The IV is rendered inactive by the development of a gold-disulfide link between porous gold NPs (PoGNPs) and HA, which prevents membrane fusion and viral internalization.^258^ NPs may also bind with viral DNA or RNA to exert intracellular antiviral effects. AgNPs have been shown to inhibit by complexing S and O groups of thiols and phosphates on nucleic acids and amino acids, or by directly binding DNA or RNA to reduce the rate of viral reverse transcription.^262^ Glutathione (GSH)-Capped Ag_2_S Nanoclusters inhibit the synthesis of viral negative-strand RNA. Ivermectin can inhibit the replication of viral nucleic acids, and a clinical study has shown that topical application of ivermectin mucosal adhesion nano-suspension nasal spray is safe and effective in the treatment of patients with mild COVID-19. Respiratory manifestations (loss of olfactory sensation, coughing, and dyspnoea) can be rapidly restored in patients following treatment (NCT04716569).^263^ In addition, the NPs also induce the production of IFN-stimulated genes (ISGs) and pro-inflammatory cytokine, thereby potentially preventing the infection of porcine epidemic diarrhea virus.^264^ Nanovectors can block the interaction between the viral ligand and receptor on the host cell, thereby inhibiting the entry of viruses. This involves mechanisms such as receptor mimicry, spatial blockade, and chemical competition. Receptor mimicry is based on designing the nano carrier to have a structure or surface properties similar to the viral ligand, allowing the nano carrier to bind to the viral ligand and mimic its interaction with the cell receptor. By competitively binding to the viral ligand, the nano carrier can inhibit the normal interaction. AgNPs have been shown to block the gp120-CD4 interaction of HIV and even control infection by complexing S and O groups of thiols and phosphates on nucleic acids and amino acids, or by directly binding DNA or RNA to reduce the rate of viral reverse transcription.^262^ As same as nucleic acid inhibitors, long-acting ribavirin NPs demonstrate long-lasting (>4 months) inhibition of HIV replication in the rectum in a Phase 1 clinical study (*p* < 0.0001) (NCT01656018).^265^ Similarly, positively charged ZnO NPs block the interaction of SARS-CoV-2 with host cell receptors to disrupt virus-host cell binding.^266,267^ The presence of the nano carrier can introduce additional space, allowing it to compete with the viral ligand for binding sites on the cell receptor. As a result, the viral ligand is unable to effectively bind to the cell receptor. Mercaptoethane sulfonate-covered silver NPs (Ag-MES) inhibit herpes simplex virus-1 (HSV-1) infection by blocking viral attachment and entry into cells.^268^ AuNP spheres of 7.86 ± 3.3 nm size could interfere with the attachment of virus to Vero cells, inhibiting HSV-1 infection.^269^ Nanocarriers can competitively bind to viral ligands through mechanisms such as chemical interactions, charge interactions, hydrophilicity/hydrophobicity interactions, and other means. Fe_2_O_3_ and Fe_3_O_4_ NPs have been reported to alter the conformation of glycoproteins (E1 and E2) of and the stinging protein RBD of SARS-CoV-2.^266^ Boronic acid-modified lipid nanocapsules (BA-LNCs) are thought to prevent HCV from entering cells by forming a cyclic diester between the glycan on the HCV envelope protein and the BA part of the LNC.^270^ Highly positive-charged chitosan NPs interact electrostatically with negatively-charged viral surfaces.^271^ For example, the leading agent for human influenza in 2009 was the human influenza A/Puerto Rico/8//1934 (H1N1) virus (PR8), a subclass of IAV with a negative charge that is readily linked with positively charged polymers like chitosan or trimethylchitosan.^272,273^ In addition, targeted editing of the viral genome using the clustered regularly interspaced short palindromic repeats (CRISPR)-Cas system can also achieve the purpose of inhibiting viral replication and infection. One study used the CRISPR-Cas13 system to design and screen CRISPR RNAs (crRNA) that could target conserved viral regions and named PAC-MAN. Cas13d PAC-MAN showed excellent antiviral activity and effectively reduced H1N1 IAV load in respiratory epithelial cells.^274^ Subsequent work found that PAC-MAN inhibited many SARS-CoV-2 variants and multiple human coronavirus strains, reducing viral titers by >99%, as PAC-MAN can inhibit coronaviruses through cytosolic co-localization of crRNA with Cas13d and target viral RNA.^236^

##### Activating immune responses

NPs can initiate specific immune responses to facilitate the recognition and clearance of viruses by immune cells. In addition to the enhanced antigen presentation and immune stimulation mentioned earlier, NPs can also activate pattern recognition receptors (PRRs) and induce IFN production.

NPs trigger an immune response via activating PRRs. On one hand, the surface structure and composition of NPs can directly interact with PRRs, activating the signaling pathways of PRRs. For example, Montague et al. reported that surface-charged NPs can interact through charge interactions, binding to and activating platelet (PLT) glycoprotein receptors, enabling them to function as PRRs for both endogenous and exogenous charged ligands.^275^ On the other hand, certain NPs can be taken up by immune cells and enter the intracellular space. Within the cells, molecules released by the NPs can interact with PRRs, activating the corresponding signaling pathways, which leads to the activation of immune cells and initiation of an immune response. Zhang et al. reported that lipid-based NPs carrying mRNA vaccines can efficiently deliver mRNA to APCs while simultaneously activating TLR4 and inducing robust T cell activation.^276^ Notably, some NPs can activate PRRs without the need for phagocytosis. For example, Yazdi et al. reported that TiO2 NPs can trigger NLRP3 inflammasome activation signal without particle phagocytosis, leading to the release of IL-1α and IL-1β within cells, causing pulmonary inflammation.^277^ These examples indicate that the mechanisms of NP-induced PRR activation are complex, and different types of NPs may activate PRRs through different mechanisms. Therefore, in the design and application of NPs, it is important to consider the interaction mechanisms between NPs and PRRs in order to achieve the desired immune effects.

The NPs can also exhibit antiviral effects by inducing the expression of IFN and ISGs. Carbon dots (CDs) have been shown to significantly induce endogenous IFN and ISG production, thereby inhibiting viral replication.^278,279^ The antiviral activity of CDs was improved by surface chemical modification.^280^ In order to encapsulate 2’,3’-cyclic guanosine monophosphate-adenosine monophosphate (GAMP), an agonist of the IFN gene stimulant STING, Wang et al. created pulmonary surfactant (PS)-biomimetic liposomes and the PS-GAMP significantly enhanced influenza vaccine-induced humoral and CD8^+^ T cell immune reactions in mice.^281^ Similarly, Gly-CDs prepared by glycyrrhizic acid and CD together can also regulate the mRNA expression level of ISGs. In addition, Gly-CDs inhibit porcine reproductive and respiratory syndrome virus (PRRSV) replication by inducing the expression of host-restricted factors, such as DDX53 and NOS3, that are directly related to PRRSV proliferation.^282^

In addition, NPs modulate immune responses by preventing excessive formation of ROS. As the natural immunity is blocked by the viral infection-induced intracellular ROS, inhibitors that reduce ROS levels can probably stop the proliferation of viruses. Gly-CDs control the levels of intracellular ROS to prevent PRRSV replication.^282^ In this regard, oseltamivir-modified AgNPs (Ag@OTV) were more thoroughly investigated for their ability to inhibit virus activity. Ag@OTV significantly inhibited the levels of phosphorylated p53 and total p53 in MDCK cells, which suggests that Ag@OTV inhibits H1N1 IV-induced apoptosis in MDCK cells by activating the ROS-mediated activation of AKT and p53 phosphorylation.^283^

#### Nanotechnology in virus detection

**Fig. 4:**
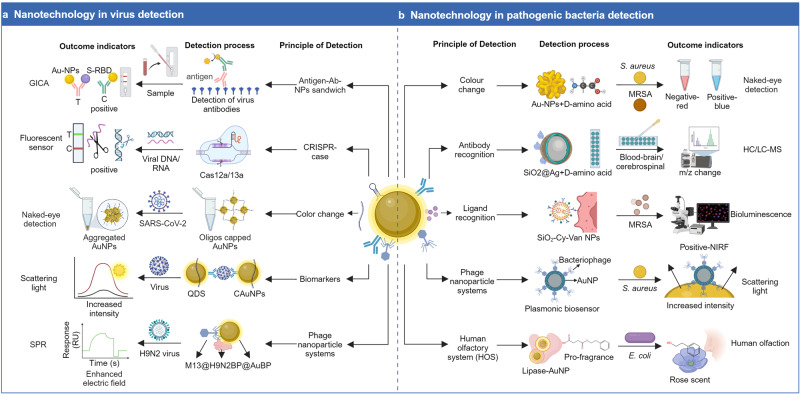
Nanotechnology in virus and pathogenic bacteria detection. This picture represents the detection principle, process and outcome indicators of nanotechnology. **a** Nanotechnology for virus detection: AuNPs based on the Antigen-Ab-NPs sandwich principle can be used to detect antibodies after vaccination with COVID-19,^296^ colloidal gold immunochromatographic assay; nanoprobes based on the CRISPR-CASE principle can be used to detect viral genes;^800,801^ capped by oligos AuNPs aggregation phenomenon and color change to detect SARS-CoV-2;^320^ AuNPs as biomarkers to track viral infections;^313^ NPs based on phage technology enhance SPR effect in virus detection.^802^ **b** Nanotechnology in the detection of pathogenic bacteria: AuNPs for the detection of bacteria by color change;^429^ SiO2@Ag^+^ detects infections in postoperative cerebrospinal fluid;^314^ SiO2-Cy-Van NPs detect MRSA by recognizing bacterial ligand MRSA;^431^ phage-coupled nanoparticles enhance the detection signal for *S. aureus*;^434^ Lipase-AuNP detects Escherichia coli by generating specific aroma.^435^ MRSA: methicillin-resistant *Staphylococcus aureus*

The prevention of the epidemic and the treatment of diseases induced by viruses depend on the early diagnosis of infected microorganisms (Fig. 4a). Compared to traditional methods, nanotechnology has potential advantages in the detection and diagnosis of viruses. The size advantage of nanomaterials shortens the analysis time and improves the detection sensitivity, providing critical support for early diagnosis.^284^ Nanotechnology can also reduce sample consumption during the detection process, and integrate different detection modes such as optical, electrochemical, magnetic, etc., to provide complementary information and increase the reliability and accuracy of the detection results.^285–288^ Furthermore, the miniaturization characteristic of nanotechnology allows virus detection devices to be smaller, lighter, and even portable.^289^ This enables virus detection to be conducted outside the laboratory, in scenarios such as field settings, remote areas, or places with limited access to medical resources.^290^ In this regard, some nanomaterials, including AuNPs, ZnO/Pt-Pd, magnetic and QDs and graphene have been developed in recent years for virus detection and tracking.^287,289,291–293^ In recent years, there has been extensive research on applying nanotechnology to improve traditional virus detection methods, nanosensors and virus labeling. These studies have confirmed the tremendous potential of nanotechnology in the field of virus detection.

Nanotechnology can be used to improve traditional virus detection methods. Previously, semiconductor QDs were used to address the disadvantages of conventional fluorescent dyes in ELISA such as easy photobleaching, low quantum efficiency and wider fluorescence spectra. This is an advantage of QD as a marker in ELISA in place of traditional enzyme markers, and in recent years it has been found to also provide more sensitivity, stability, and multiplexing capabilities, contributing to the sensitivity and reliability of immunoassays. Using enzymatic in situ generation and immobilization of CdS QDs onto microspheres for the detection of the cancer biomarker superoxide dismutase 2, the immunoassay using enzymatically generated CdS NPs by electrochemical and fluorescence detection would have better detection limits of at least 3 orders of magnitude compared to previous studies.^294^ Similarly, Kurdekar et al. reported that a paper-based immunoassay platform based on carbon QDs demonstrated a higher detection range and shorter detection time for HIV antigen monitoring compared to traditional ELISA detection methods.^295^ A study developed a colloidal gold immunochromatographic assay (CGICA) based on the dual-antigen sandwich principle of AuNPs can detect both IgM and IgG antibodies against SARS-CoV-2 in human blood in a short period of time, which have high sensitivity and specificity.^296^ In comparison to ELISA and the rapid lateral flow immunoassay (LFIA), the lateral flow QIAreach anti-SARS-CoV-2 Total rapid NP fluorescence immunoassay not only shows comparable performance to ELISA but also has a shorter and simpler manual handling than LFIA.^297^

The CRISPR-Cas technique represents a groundbreaking gene editing technology and has emerged as a widely utilized nucleic acid assay with numerous applications.^298^ In contrast to traditional RT-qPCR and immunoassays, CRISPR/Cas-based methods offer significant advantages in viral detection, including scalability, rapidity, visual detection, high sensitivity, and specificity.^299–302^ Cas12a and Cas13a proteins can selectively target DNA and RNA, allowing them to play a crucial role in viral detection.^303–307^ For instance, the DNA Endonuclease-Targeted CRISPR Trans Reporter (DETECTR), based on Cas12, can detect SARS-CoV-2 in a significantly shorter time.^308^ Especially, the specific high-sensitivity enzymatic reporter unlocking technology (SHERLOCK) based on Cas13 demonstrated 100% specificity and 96% sensitivity when analyzing 154 clinical samples from COVID-19 patients, with sensitivity comparable to RT-PCR.^309^ The naked-eye detection of viruses can be carried out by combining nanotechnology with genetic detection techniques.^310^ The complementary cDNA sequences of viral proteins are loaded on gold nano-islands to make antisense oligonucleotide (ASO) capped plasma NPs. With the N-gene targeting NPs, SARS-CoV-2 can be selectively detected by colorimetric means without the need for complex instrumentation. These ASO-capped plasma NPs make naked-eye detection of SARS-CoV-2 possible by N-gene targeting-mediated selection.^311^

Nanotechnology can also be used to develop electrochemical sensors. These sensors utilize the unique properties of nanomaterials in electrochemical reactions to monitor the changes in current, potential, or impedance caused by viruses. AuNPs were employed in a dielectric electrophoresis array electrode in a study that generated a novel, low-cost electrochemical immunosensor for detecting MERS-CoV.^312,313^ Au^3+^-based ethylenediaminetetraacetic acid disodium salt (EDTA-2Na) chelators showed an increase in detection sensitivity compared to gold standard assays for HBsAg and alpha-fetoprotein by three more than three orders of magnitude.^314^

NPs can be used as viral markers to enable the rapid qualitative or quantitative detection of viruses by leveraging visual or fluorescence signal changes. For example, QDs are basic nanomaterials that are highly bound to fluorescent samples and can be applied for continuous fluorescence imaging and process-specific detection of cells.^313^ Self-assembled star-ridge plasmonic AuNPs published in 2017 were shown to help achieve the lower limit of detection values for detecting avian influenza A H5N1 virus concentrations at pmol levels, significantly improving detection sensitivity.^315^ In this study, self-assembled chiral QDs and AuNPs nanostructures for the detection of infectious bronchitis virus (IBV) in chicken blood samples were found to be highly effective for IBV detection.^315^ Metal NP probes may offer a crucial contribution to the identification of viruses via dark field microscopy.^316^ In addition, NPs as biomarkers can also be used to track the viral life cycle. During viral replication, streptavidin-coupled QDs labeled with self-biotinylated baculovirus nucleocapsid proteins were specific and practical, while did not affect viral infectivity.^317^ Duval et al. used single-particle imaging to monitor the uncoating of particular IAV virions by embedding QD-conjugated virus ribonucleoprotein combinations inside pathogenic IAV virions. Their research reveals vRNP trafficking and uncoating mechanisms, which could help researchers create novel methods to prevent IAV infection.^318^

Biomaterial-based nanodetection technologies have rapidly advanced. Phage-based nanoprobes have been developed and are being utilized for disease detection.^319^ Quantification of miRNAs through T7 phage enables effortless and precise detection of miRNAs even without the aid of laboratory equipment.^320^ Phage-based gold NPs may enhance the detection sensitivity significantly. Taking M13@H9N2BP@AuBP as an example, it exhibits an SPR with an electric field 40 times higher than that of traditional AuNPs. This results in a high detection sensitivity for H9N2, reaching as low as 6.3 copies/mL (approximately 1.04 × 10^−5^ fM).^287^

These applications demonstrate that nanotechnology has advantages in the field of virus detection, including high sensitivity, high specificity, rapid detection, and reliability. With the continuous development and innovation of nanotechnology, we can expect to see more virus detection methods based on nanotechnology, providing strong support for disease prevention and control.

### NPs in pathogenic bacterial and fungal infections

Pathogenic bacterial infections, alongside viral infections, have become a significant contributor to the burden of disease. The prevalence of single or multidrug-resistant bacteria poses a significant threat to existing antimicrobial agents.^321,322^ Recently, nanotechnology has shown promising applications in bacterial and fungal infectious diseases. In this section, we described the application of NPs in the prevention, treatment, and detection of bacterial and fungal infections. Emerging nanovaccine technologies are contributing to the rapid development of more effective and long-lasting antimicrobial vaccines. Nanomaterials can perform multiple bactericidal pathways, including the delivery of conventional antibiotics, interaction with cell walls or cell membranes, disruption of the internal structure of the bacteria, and removal of toxins produced by the bacteria. Nanotechnology also holds new promise for the detection of bacteria.

#### Nanotechnology in the prevention of pathogenic bacterial infections

NPs, with their obvious advantages as vaccine adjuvants and delivery vehicles, are also playing an increasingly important role in the prevention of bacterial infections (Fig. 3a). The implementation of nanotechnology in antimicrobial vaccines may enhance the biocompatibility, immunogenicity and antigen presentation ability. In addition to pathogen-specific methods such as vaccines, directly targeting the source of infection in the environment and cutting off the transmission routes are also effective ways to prevent bacterial infections, such as novel NP fungicides and disinfectants.

##### Nanotechnology used for antimicrobial vaccines

The emerging nanovaccines that are currently being used for the prevention of pathogenic infections fall into the following broad categories: DNA vaccine,^323,324^ mRNA vaccine,^325^ protein vaccine,^326^ OMV,^327,328^ and nanotoxoids.^329^

As a third-generation vaccine, DNA vaccines have long been used to prevent foreign invaders by triggering humoral and cellular immune responses against foreign genes.^330^ The shortcomings of low immunogenicity of DNA vaccines are being overcome by the advent of nanotechnology.^331^ Various nanomaterials have been developed for DNA vaccine delivery, including polymeric NPs,^332^ liposome NPs,^333^ virus-like particles,^334^ and self-assembling proteins.^335^ PLGA has been widely used in the design and development of DNA vaccines against tuberculosis because of its immunological properties and good biocompatibility.^336^ Compared to naked plasmid-DNA vaccines, delivery based on liposomal nanomaterials effectively prevents extracellular nucleic acid endonucleases from degrading DNA through interactions with the cell membrane, thus improving the efficiency of plasmid-DNA transfection presentation.^337^ In addition, modified liposomes with appropriately targeted ligands stimulate and activate immune signal pathways via PRRs, leading to maturation of APCs and antigen processing and presentation.^333^

mRNA vaccine technology has rapidly advanced during the COVID-19 pandemic, with well-demonstrated safety and efficacy.^338^ In addition to its application in oncology and viral infections, mRNA vaccines could also prove beneficial in combating bacterial and fungal infections. Nevertheless, when compared to viruses, bacteria are known to express thousands of proteins, which presents a challenge in choosing the appropriate protein antigen to target. Despite the high potential of mRNA vaccines in treating (intracellular) bacteria, only a limited number of studies have explored this promising avenue.^339,340^ Mayer et al. identified 42 Listeria monocytogenes immune peptides from 68 different bacterial antigens. Subsequently, they incorporated these highly expressed antigens as vaccine candidates in mRNA vaccine formulations that significantly reduced bacterial load in the liver and spleen.^341^ Moreover, drug tolerance is a major problem in bacterial and fungal infections, and the rise of mRNA vaccine technology is expected to attenuate antimicrobial resistance (AMR).^342^ Recently, Maruggi et al. investigated the immunogenicity and efficacy of a self-amplified mRNA (sa-mRNA) vaccine against group A (GAS) and group B (GBS) streptococci.^340^ The sa-mRNA vaccine elicited antibody responses and provided protective effects, along with the induction of antigen-specific IgG2a (Th1) responses. This study demonstrated the potential of mRNA vaccines against AMR pathogens.

OMVs are ~20–250 nm nanostructures produced by vesiculation of the bacterial cell envelope.^343^ The vesicles contain a variety of membrane proteins that enhance PRR binding to PAMP on APCs, thereby activating the immune system.^344^ The small nanoscale size of OMVs facilitates their penetration into lymph nodes and boosts the uptake by APCs, thus enhancing immune activation. OMVs have progressed to the clinical phase as an antimicrobial vaccine, including those derived from Streptococcus meningitidis and Staphylococcus aureus.^345^ Besides, OMVs can also be genetically engineered and modified.^346^ For instance, enhancing the genetic makeup of *E. coli* to produce OMVs that contain modified polysaccharides offers a potential strategy to enhance the effectiveness of vaccines.^347^ OMVs can also be combined with NP delivery systems to enhance vaccine efficacy. The release of OMVs by Shigella fowleri bound in poly(anhydride) NPs enhances bioadhesive interactions and triggers innate immunity mediated by TLR2 and TLR4.^348^ Furthermore, the OMVs can be utilized as a delivery system, and if encapsulated with other NPs, the efficacy can be improved. OMV-encapsulated AuNPs can be employed to precisely regulate the size distribution of the ultimate formulation.^349^ This method not only improves the stability of AuNPs but also takes advantage of the multivalent antigenic properties of OMVs.

Germ infections are frequently accompanied by the discharge of harmful toxins, which can result in severe consequences like cellular damage, haemolysis, the destruction of the immune system and even sepsis. The toxoid vaccine strategy can efficiently avert the poisonous outcomes of germ infections and decrease host stress.^350,351^ Nanotoxins based on NP designs have been used to enhance the effectiveness of vaccines.^352^ Nanotoxins increase protective immunity and antibody titres in vaccinated mice with lower cytotoxicity compared to conventional inactivated toxin vaccines. Recent studies have shown that erythrocyte-based nanotoxins, are capable of neutralising a wide range of bacterial toxins, including alpha toxin,^353^ bee toxin, listeriolysin O and streptococcal haemolysin O.^354^ These nanotoxins effectively prevent toxins from binding to cell membranes. In addition, cell membranes are used to encapsulate various types of bacterial toxins, resulting in the development of nanovaccines with a broader range of targets. The cell membrane structure of these NPs allows them to be compatible with different cell membranes, enhancing their versatility.^355,356^ For example, NPs encapsulated with mouse macrophage membranes (MM) have demonstrated the ability to capture and neutralize endotoxin activity such as lipopolysaccharide (LPS).^357^ The adjustability of the cell membrane coating and the versatility of the various toxins enable the creation of a nanotoxin-based platform that can serve as a vaccine against numerous pathogenic bacteria, encompassing both Gram-negative and Gram-positive strains.

In conclusion, existing research has shown that vaccines based on various nanotechnology approaches can achieve effective prevention of pathogenic bacterial infections. This significantly reduces the risk of widespread bacterial infections and also alleviates some of the burden in treating drug-resistant bacteria.

##### Nanotechnology used for pathogens eradication and transmission prevention

In addition to vaccination for enhancing host immunity, using antimicrobial materials to directly inhibit or kill pathogenic microorganisms in the environment and disrupt their transmission is a straightforward and highly efficient method for source control. Currently, most of these materials are metallic NPs, including AgNPs,^358,359^ AuNPs,^360^ Carbon-modified AuNPs,^361^ copper NPs,^362^ and cerium oxide NPs.^363^ Besides, polymeric NPs and carbon-based NPs are also employed in the prevention of pathogenic bacteria.^364,365^ These NPs are often found in the form of disinfectants or other protective gear. For example, disinfectants containing AgNP can kill bacteria and fungi in a broad-spectrum manner.^366,367^ Tissues coated with selenium NPs effectively inhibit the growth of a wide range of Gram-positive and Gram-negative bacteria.^368^ Textiles functionalized with CuO NPs can be made into masks, gloves, wipes, etc. with excellent antibacterial effects.^369^

Pathogenic bacteria in the environment are typically found suspended in the air, present in water, or adhering to solid surfaces. Therefore, the main strategies for preventing bacterial infections include bactericidal actions, anti-biofilm measures and antiadhesive effects.^370,371^ Studies have shown that the small particle size of AgNPs can easily enter the interior of bacteria and destroy cell walls and membranes. Simultaneously, AgNPs can generate ROS and free radicals, destroying cell contents and leading to cell death, thereby achieving broad-spectrum antibacterial effects.^372^ In terms of the prevention of adhesion and biofilm formation by Candida species, a number of nanomaterials have been developed.^373^

In addition, nanotechnology-based antimicrobial materials can be applied in various potential infection scenarios.^374^ The most common situations involve infections originating from food and the oral cavity. Utilizing nanomaterials to eliminate pathogenic microorganisms within food products and preventing disease entry through the mouth is a highly effective method for preventing microbial infections.^375^ For example, coating palladium NPs and platinum NPs on food packaging bags can effectively kill common food-borne pathogens *Salmonella enterica infantis*, *Escherichia coli*, *Listeria monocytogenes*, and *Staphylococcus aureus*, etc.^376^ Implant-associated infections are also a major problem in current surgical procedures, and a variety of nanomaterials have been used for surface modification of implants, such as FeNPs, AgNPs.^377,378^ NPs are also interesting materials for promoting wound healing, and AgNP polymers embedded in wound dressings inhibit microbial growth and promote wound healing.^379^ In addition, L-arginine-containing mesoporous silica NPs were used in dental adhesives to effectively prevent dental caries.^380^

In conclusion, nanotechnology-based materials have been able to effectively prevent bacterial and fungal infections in multiple forms and pathways.

#### NPs in antibacterial therapy

Once the bacterial or fungal infection occurs, the human system will be affected by a series of inflammatory reactions, leading to various degrees of tissue damage and even systemic infections with serious consequences. Nanotechnology-based drugs have shown promising results in the treatment of such infectious diseases.^381–384^ Here, we summarized the application of NPs in the treatment of pathogenic bacterial infections and their mechanisms (Fig. 3c), including the delivery of drugs to overcome the drawbacks of conventional antibiotics, the direct targeting and killing of bacteria at the site of infection, or the removal of bacterial toxins to reduce the extent of damage.

##### Nanomaterials as antimicrobial delivery vehicles

The main advantage of NPs over traditional delivery systems is their size. The size of NPs is very small and controllable, suitable for carrying antimicrobial agents and against bacteria. NPs-based antimicrobial delivery effectively improves the bioavailability of antibiotics, reduces toxicity and side effects, overcomes drug tolerance, and enhances resistance to intracellular infection. The poor solubility, first-pass effect and inadequate targeting of traditional antibiotics contribute to their low bioavailability, which can be overcome by nanotechnology drugs.

NP delivery systems can improve the performance of prodrugs in the following ways: reducing the particle diameter to facilitate absorption,^385^ increasing the surface charge of particles to enhance drug binding to cells,^386^ and altering the oil-water partition coefficient to increase drug water-solubility or lipophilicity.^387^ Self-assembled chitosan NPs developed by Wu et al. can improve the dispersibility of licoricidin, effectively overcoming the poor water solubility, instability, and low bioavailability of natural flavonoids.^388^ In addition, the preparation of antibiotics as nanocrystals is also a means of overcoming poor bioavailability: dapsone nanocrystal suspensions produced by Nataly et al. are smaller, more soluble and more stable compared to the original drug.^389^ SLNs loaded with antimicrobials were found to improve the effectiveness of fluconazole (FLZ) in treating phytolacca vulgaris. Clinical studies demonstrated that FLZ-SLNs exhibited high encapsulation rate, good stability, prolonged release time, and higher cure rate compared to Candistan 1% cream.^390^ Nano-curcumin possesses potent antifungal properties analogous to mycobacteria. However, its therapeutic effects have slower onset compared to mycobacteria. The enhanced bioavailability of curcumin in nano form makes it a promising alternative therapy for thrush, with the potential to avoid diseases associated with mycophenolate.^391^

Antibiotics have prevalent adverse effects including GI irritation, damage to human commensal microorganisms, liver, and kidney toxicity, etc. Formulating multiple drugs within a nanocarrier system can enhance efficacy by increasing local concentrations while decreasing systemic concentrations, leading to fewer side effects. Gounani et al. loaded polymyxin B and vancomycin (VAN) onto NPs to achieve simultaneous local delivery of antibiotics against both Gram-positive and Gram-negative bacteria, which effectively reduced the required drug dosages.^392^ Fernández et al. assessed a nystatin nanoemulsion optimal for topical use, circumventing risks like systemic absorption and toxicity.^393^ Furthermore, NP modification can reduce drug toxicity. Ludmila et al. developed a stable chitosan-conjugated AgNPs form exhibiting non-toxicity to normal fibroblasts even at high concentrations.^394^

The application of NPs represents a promising approach to overcome microbial drug resistance. Simultaneously encapsulating multiple antimicrobial agents within a single NP can effectively eradicate bacteria and reduce the development of resistance.^381^ In addition, the intrinsic and extrinsic properties of NPs, such as their functional groups, concentration, and size, significantly influence the development of antibiotic resistance.^395^ Recent studies have made advances in the surface modification of nanocarriers for antimicrobial delivery.^396^ For instance, folic acid (FA)-modified LNPs improved the efficacy of VAN therapy.^397^

NP delivery systems are promising tools for the treatment of intracellular bacterial infections and for the prevention of disease recurrence caused by these bacteria. The pathogenic bacteria can escape host cell killing through various pathways.^398,399^ The drug-loaded NPs are recognized as foreign substances by the MPS and phagocytosed, allowing the drug to be released from the NPs to eliminate bacteria hidden inside macrophages.^400^ Zou et al. constructed pH-responsive NPs using cinnamaldehyde (CA)-dextran conjugates as carriers and loaded with glabridin (GLA). These NPs can target macrophages infected by MRSA. They not only reduce the risk of macrophage damage and intracellular bacterial spread but also rapidly kill intracellular MRSA with a very low probability of drug resistance.^401^

##### Destruction of cell wall and cell membrane

The cell wall and cell membrane of bacteria play a crucial role in their pathogenicity and the development of drug resistance. Therefore, exploring antimicrobial treatment approaches targeting these components holds significant promise.^402^ NPs can effectively impact the cell walls and cell membranes of pathogens, leading to pathogenic damage. These actions involve direct destruction of the cell wall or cell membrane, impairment of the efflux pumps on the cell membrane, and interruption of transmembrane electron transfer.^403^

One of the primary ways in which nanotechnology drugs interact with pathogenic cell walls is through a mismatch between the charge carried by the NPs and the surface charge of the cell wall, this imbalance can lead to cell wall damage or rupture, resulting in an antimicrobial effect. Metallic NPs are typically utilized for this purpose.^404^ The AgCuE nanosystem can damage the cell wall, leading to the death of planktonic Candida albicans and achieving therapeutic results for fungal keratitis.^405^ The intracellular pH-responsive pathway serves as a vital antimicrobial tool, often in combination with cell wall disruption techniques.^406–408^ Astodrimer has proven effective in inhibiting the growth of microorganisms involved in the pathogenesis of bacterial vaginosis. Its antimicrobial mechanism focuses on blocking bacterial adhesion, disrupting biofilm formation, and preventing further biofilm development. Several clinical trials have been conducted to evaluate its effectiveness.^409,410^ Similar to disrupting the cell wall mechanism, NPs can also act as antimicrobial agents by modifying the membrane potential and impeding cell membrane formation and maturation.^411^ Furthermore, multi-principal element NPs represent an emerging class of materials with potential applications in medicine and biology.^412^ NPs can also absorb bacteria, leading to increased membrane tension and deformation, ultimately resulting in cell rupture and death.^413^ In addition, a more precise method is to devise NPs that target proteins in the bacterial cell membrane. The CBD-EGFP-BP-AgNP complexes developed by Domyoung Kim and colleagues have the ability to specifically recognize the target cells and exhibit superior bactericidal effects.^414^ Khare et al. designed a set of multi-combination NPs named Emb-Chi-Au NPs, which can be used against multidrug-resistant bacteria by suppressing the efflux pumps on bacterial membranes.^415^

##### Production of ROS and damaging intracellular component

Affecting the production and maturation of bacterial or fungal cellular contents is also a major antimicrobial strategy. Nanomedicine can not only invade pathogens and directly kill bacteria by destroying nucleic acids and proteins inside them. It can also induce the production of ROS in microorganisms, damaging cellular components and thereby indirectly killing pathogens. In addition, it can also induce the production of ROS at the site of infection for antibacterial effects. Bacteria contribute to the dissemination of antibiotic resistance genes in the environment by ingesting and directly converting extracellular DNA (eDNA). Xu et al. treated Escherichia coli with CeO_2_ NPs, which inhibited the expression of DNA uptake and processing-related genes by directly binding eDNA, reducing ROS levels and cell membrane permeability. This antimicrobial strategy can inhibit bacterial growth and reproduction at the source.^416^ Different types of SeNPs designed by Fresneda et al. can exert their antibacterial effects by increasing the ROS content in bacteria. Among them, undefined-SeNPs perform best in inducing DNA damage (around 80% of DNA degraded).^417^ The multifunctional gold-silver carbon QDs nano-hybrid composite constructed by Ang and colleagues can also increase the ROS content in bacteria and play an antibacterial role under both UV irradiation and non-irradiation conditions.^418^

Interestingly, infection of the body by pathogens is accompanied by a series of inflammatory responses, which include the production of ROS. As powerful oxidants, ROS have the ability to completely disrupt the entire biofilm and play an important biological role in the defense against pathogens.^419^ How can the production of endogenous ROS act selectively as an antibacterial agent? Currently, nanoenzymes are commonly used in this antimicrobial approach. Nanoenzymes are artificial enzymes that imitate the activity of natural enzymes and generate antimicrobial effects by accelerating the production of ROS.^420,421^ The efficient antimicrobial activity of nanoenzymes hinges on the unification of enzyme-like activity and bacterial binding capacity within a single drug. The development of ROS nanoenzymes was reported by Gao et al. that are attached to a surface and are capable of targeting and destroying bacteria selectively in mammalian cells. In addition, these enzymes can reduce the risk of drug resistance.^422^ GSH is an important intracellular antioxidant that neutralizes ROS. Topical application of GSH-cyclodextrin NP complex (GSH-CD) showed beneficial therapeutic effects on mycobacterial infections.^423^ After three consecutive days of GSH-CD application, GSH levels increased, MDA levels decreased and the prevalence of *Mycobacterium avium* infection in whole blood cultures of the clinical trial participants was significantly reduced (NCT05926245).

##### Targeting bacterial toxins

During the procedure of bacterial infection, bacterial cells can secret toxins to damage host tissues, which will enable the penetration of bacteria into deep tissue.^424^ NPs can remove bacterial toxins by inhibiting the secretion of toxins from pathogens, binding and removing bacterial toxins.

A toxin-responsive NP was designed by Han et al.^425^ When NPs encounter bacteria within the body, the nanoreactor can capture and break down any toxins secreted by the bacteria. A biomimetic nanosponge that absorbs pore-forming toxins was reported by Hu et al. NPs that are coated with red blood cell (RBC) membranes are utilized as treatment for bacterial sepsis by neutralizing multiple kinds of bacterial toxins and killing bacteria.^426^ For intracellular infection, Zou et al. found that GLA-loaded pH-responsive NPs released CA downregulated the expression of cytotoxic pore-forming toxins, which reduced the risk of macrophage damage and intracellular bacterial dissemination.^401^ A class of reactive metal boride NPs was synthesized by Meng et al. This NP has the capability to capture LPS or PGN. This function not only inhibits excessive inflammation that is caused by dead bacteria in vitro and in vivo but also stimulates wound healing effectively.^427^

#### Nanotechnology in pathogenic bacteria detection

Nanotechnologies for targeted pathogen detection are typically categorized into three groups: antibody or aptamer-based detectors, phage-based sensors, and olfactory system-based assays. Nanotechnology detectors based on aptamers facilitate improved specificity (Fig. 4b).^428^ Yang et al. designed a broad-spectrum bacterial detection system based on D-amino acid-capped AuNPs. This NP has the ability to specifically target bacteria and promptly differentiate between *S. aureus* and MRSA by means of color changes.^429^ A rapid, sputum-free TB detector has been developed and is in clinical research (NCT03271567). This detector uses antibody labeling and energy-focused porous disk-shaped silica NPs (nanodisks) and high-throughput mass spectrometry to enhance sensitivity and specificity. Similar robust sensitivities were obtained in both culture-positive pulmonary tuberculosis (PTB; 91.3%) and extrapulmonary tuberculosis (EPTB; 92.3%) cases. Sensitivity in HIV-positive patients was also superior to that of routinely reported detectors.^430^ Modified NPs are typically detected through the recognition of bacterial ligands with the aid of optical imaging, electrochemical sensing or spectroscopy. An activatable theranostic nanoprobe was reported to the treat of MRSA infections.^431^ This specifically developed nanoprobe facilitates quick NIRF imaging and prolonged tracking of MRSA infection progression. The approach of recognizing antibodies specific to the bacterial surface has advantages in terms of both specificity and sensitivity. AuNPs sensitively and selectively detect and differentiate *Salmonella typhimurium* DT104 (103 CFU g^−1^ or more) from other genera and species on long leaf lettuce infected with a bacterial mixture of *Escherichia coli* and *Salmonella typhimurium* DT104 by changing the color of the NP solution from purple to gray.^432^ RBPs from the genus Inovirus were genetically modified for expression as phage scaffolds on M13, and the resulting chimeric phages were employed for the detection of the desired bacterial species.^433^ A sensor employing phage as a biorecognition element for the rapid and sensitive detection of excess *Staphylococcus aureus*. The procedure involves combining the sample with a *Staphylococcus aureus* phage S13-conjugated equipartitioned exciton scattering probe, followed by detection within 15–20 min, with a detection limit of 8 × 10^4^ colony-forming units per milliliter.^434^ Nanotechnological detectors based on the olfactory system used for bacterial infections have been developed in recent years.^435^ The sensor platform utilizes NPs to reversibly bind and hinder lipase. When bacteria are present, these complexes are destroyed, which then restores enzyme activity and creates odor from odorless pre-scented substrate molecules. This system provides swift (15 min) sensing, and an exceedingly high sensitivity (102 CFU/mL) for bacterial detection—utilizing human olfaction as the output.^435^ Detection and diagnosis of complex and microscopic samples such as metabolites is heavily dependent on material design. An optimized SiO_2_@Ag nanoshell structure is already in clinical trials for the efficient detection of biomarkers and metabolites in patients with post-surgical brain infections.^436^

### Other infectious diseases

In addition to viruses, bacteria and fungi, parasitic infections are an important component of infectious diseases, and their unique life histories make them widely transmissible, with an urgent need to develop new preventive diagnostic strategies to prevent their spread. In addition, transmissible spongiform encephalopathies (TSEs/prion diseases) are a group of infectious diseases that are difficult to treat. This section will discuss the application of nanotechnology to these two major groups of diseases.

#### NPs in parasitic infections

Owing to changes in the living environment of human beings and the rapid development of modern medicine, many parasites are on the verge of being eliminated by human beings. However, there are still parasites such as plasmodium and tapeworms that cause great harm to humans. Currently, some NPs, including liposomes, polymer NPs, SLNs, nanosuspensions, etc., have been investigated for the delivery of antiparasitic drugs. Nanomedicines combat parasitic infections through multiple pathways: inhibiting parasite entry into host cells and reproduction, directly killing parasites in the environment, prolonging drug action, and stimulating immune responses (Fig. 3d).

Najer presented a 3D nanoobjects very efficiently blocked invasion of RBCs by P. falciparum merozoites compared to soluble receptors.^437^ Nanovaccines for falciparum malaria are presently undergoing clinical studies. The R21 vaccine, which is based on the circumsporozoite protein and administered with adjuvant Matrix-M, has proven to be both safe and highly effective. Volunteers inoculated with the R29/Matrix-M vaccine exhibited only mild side-effects, along with a pronounced upsurge in malaria-specific anti-Asn-Ala-Asn-Pro antibodies in vivo.^438^ Malaria vaccines are currently in clinical trials, ChAd63-MVA ME-TRAP was used to prevent malaria infection in 22 healthy adults, which was effective in eliciting humoral immunity and T-cell responses.^439^ Another clinical trial also demonstrated that the vaccine prevented the progression of hepatic malaria.^440^ Biosynthesized CuO NPs were shown to exhibit efficient antiparasitic activity against cattle tick larvae.^441^ Myrrh AgNPs were first used to treat cutaneous leishmaniasis (CL) by Awad et al. Strong antiparasitic activity against Leishmania major was demonstrated in both in vivo and in vitro studies. Pfs230 TBVs are recombinant protein NPs, targeting parasite development.^442^ Currently, there are several Pfs230 clinical trials underway (NCT02942277, NCT03917654, NCT05135273), Pfs230D1-EPA has stronger immunogenicity than Pfs25-EPA(NCT02334462).^443^

The use of nanotechnology in the early stages of the development of counter parasitic diseases, but brings hope that this new field will provide a solution to the early stages of parasitic diseases, compensating for the lack of vaccines for most parasitic diseases and providing new therapeutic options for disease parasites showing increased resistance to current drugs.

#### NPs in TSEs/prion diseases

NPs possess exceptional features, comprising sensitivity, selectivity, and the capability to traverse the BBB when implemented in nano-sized particles. These NPs are extensively employed in imaging studies and treatments of neurological conditions.^444^

Thus, nanomedicine has been utilized for detecting, diagnosing and treating the Nguyen virus.^445^ NP-enabled biosensors have illustrated exceptional sensitivity, with apt detection of prion proteins, promoting speedy identification of prion diseases for improved treatment. Furthermore, gold nanorods have been utilized to detect amyloid fibrils in prion proteins effectively, utilizing chiral methods triggered by plasma NPs.^446^ Xiao, Jin et al. dual nucleic acid aptamer-enhanced magnetic particles and fluorescent QDs were applied for prion diagnostics, and were proven to be detectable in the pre-symptomatic stage.^447^ Sorokina et al. have reported the first instance of pH-dependent cationic pyridinylbenzene dendritic polymers being capable of breaking down amyloid aggregates, including the inclusion bodies of sheep prion protein, under physiological pH conditions.^448^

## Nanotechnology in non-infectious inflammatory diseases

In addition to infections, dysregulated inflammatory responses and abnormal activation of the immune system can also lead to inflammatory diseases. These diseases encompass a group of chronic conditions, including autoimmune diseases like RA and multiple sclerosis (MS), allergic conditions such as asthma and contact dermatitis (CD), as well as other conditions like AS, psoriasis, and IS. Numerous natural and synthetic drugs have been developed to combat these inflammations, such as cyclosporine A for systemic lupus erythematosus (SLE),^449^ etoricoxib for irritant CD,^450^ and Rhein for ulcerative colitis (UC).^451^ However, their limited specificity often leads to systemic toxicity or instability in bodily fluids, restricting their application. Emerging evidence suggests that NPs have extensive prospects for treating these inflammatory conditions.^9,452,453^ Among the various mechanisms of NP-based anti-inflammation, the specific delivery of drugs to the specific site of inflammation is crucial to reduce systemic toxicity. NPs can also offer context-dependent selective release of drugs in response to local stimuli, such as pH, ROS, or enzymes. Some NPs also have direct anti-inflammatory effects of NPs via regulating inflammatory mediators and immune cells. Besides their therapeutic functions, NPs, owing to their unique physicochemical properties, can facilitate in vivo imaging of inflammation, enabling timely monitoring of disease progression.

### NPs in the treatment of inflammatory diseases

NPs can aggregate at the lesion site through targeted mechanisms and enhance drug efficacy through multiple pathways. They primarily achieve inflammation treatment by controlling inflammatory factors and modulating immune cells. The second part of Table 2 summarizes clinical studies of nanotechnology for inflammatory diseases.

#### Targeted payload delivery

By delivering therapeutic cargo to sick tissues or certain organelles, targeted drug delivery methods may improve medical efficiency and lessen side effects.^454,455^ Nanotechnology can precisely deliver drugs to lesions to reduce systemic toxicity through passive targeting, receptor-ligand interactions, biomimetic cell membrane encapsulation, stimulus-responsive payload release, and inhalation delivery.

##### Receptor-ligand interactions

**Fig. 5:**
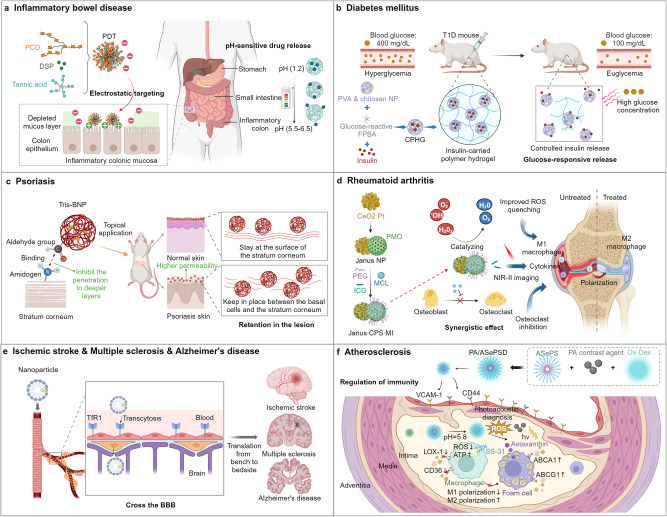
Overview of the mechanisms of NP treatment for inflammatory diseases. **a** An oral PDT that treats IBD by combining tannic acid and poly-β-cyclodextrin carrying DSP through host-guest interplay. PDT is electronegative as a result of its three parts, allowing it to electrostatically draw and target the electropositive, inflammatory colon mucosa.^803^ **b** CPHGs for regulated insulin release in the therapy of diabetes. A glucose-reactive FPBA-derived linker is used to join PVA and chitosan NPs.^509^ **c** Tris-BNPs are able to permeate solely psoriatic skin and not normal skin because of the extreme penetrability of psoriasis. Tris-BNPs gradually fade away as they spread and enter the epidermis, revealing the aldehyde chains of the BNPs, which could stick to amine chains lesional skin, enabling long-term local holding of BNPs in psoriasis skin lesions.^524^ **d** A Janus nanoplatform (Janus-CPS) for the concurrent early detection and combinatorial treatment of RA, which is made up of CeO_2_-Pt nanozyme on one part and PMO on the other. MCL, which has anti-osteoclastogenesis properties, is packed into the PMO’s mesopores to work in concert with nanozymes’ soothing properties to effectively manage RA. The NIR-II fluorescence imaging is employed by ICG-carried Janus-CPS to achieve the desired efficacy in the early detection of RA.^162^ **e** NPs penetrate the BBB through TfR1-mediated transcytosis to access the CNS, thereby treating diseases such as IS, MS, and Alzheimer’s disease. **f** SS-31 polypeptide and astaxanthin are combined in PMeTPP-MBT (a unique PA contrast agent)-loaded, smart reactive theranostic nanoplatform (PA/ASePSD). Targeting atherosclerotic lesions is enabled by PA/ASePSD’s high affinity for VCAM-1 and CD44 on impaired endothelium. Astaxanthin, SS-31 peptide, and PMeTPP-MBT are released in regulated conditions when ROS levels in acid are increased. This enables non-intrusive PA diagnostics and plaque reduction via soothing effects and lipid metabolic control.^804^ PCD poly-β-cyclodextrin, PDT polyphenol nanoparticle, PVA poly(vinyl alcohol), FPBA formylphenylboronic acid, CPHG chitosan NPs/PVA hybrid hydrogel, ICG indocyanine green, MCL micheliolide, ox-Dex oxidized dextran, PA photoacoustic, VCAM-1 vascular adhesion molecule-1, LOX-1 lectin-like oxidized low-density lipoprotein receptor-1, ABCA1/G1 ATP-binding cassette transporter A1/G1

The nanocarriers can first passively accumulate in the targeted areas by modifying physical parameters such as charge (Fig. 5a), size, and surface modification.^456^ To effectively deliver the anti-inflammatory siRNA into the lungs for treating acute lung injury (ALI), DA-grafted hyaluronic acid (HA) was coated on an anti-tumor necrosis factor (anti-TNF)-α virus, which improved mucus entry of siRNA due to the electrical screen function of HA-DA and the bioadhesive properties of the grafting DA.^457^ In addition, negatively charged NPs were used to across the intestine epithelial barrier by causing tight junction ease and improving gut penetration, thereby facilitating oral protein administration. Specifically, the diameter and charge of the NPs affect this permeation-boosting impact, with smaller (≤200 nm) and more anionic particles (like silica) offering greater permeability.^458^ However, the hydrogen bonds and electrostatic forces that underlie ligand-receptor engagement only span a distance of around 0.3 to 0.5 nm,^459^ rendering it difficult to exert a long-distance magnetic draw on a target site. Nevertheless, active targeting has the potential to improve drug delivery to inflammatory sites.^456^ For example, ligand-targeting delivery can raise the preservation of drugs on target cells or tissues by conjugating cell-specific ligands to the surface of nano drug transporters.^460^

The folate receptor β (FRβ)-directed administration for the therapy of inflammation relies on the increased expression of FR in hematopoietic cells of the myeloid lineage upon immunological arousal and the subnanomolar avidity between FR and folic acid (FA).^461,462^ Methotrexate (MTX), a derivative of FA, was carefully chosen as a disease-modifying antirheumatic medication (DMARD) due to its specific affinity to FRs.^463^ Moreover, to enhance its anti-inflammatory efficacy, the modification of 1,2-distearoyl-sn-glycero-3-phosphoethanolamine-N-[folate(polyethylene glycol)-2000] (FPD) was added to MV/MTX@ZIF-8 NPs because activated macrophages significantly overexpress FR.^464^ Research findings indicate that activated macrophages with overexpression of FR exhibit substantially higher absorption capacity for FPD/MV/MTX@ZIF-8 compared to inactive macrophages.^463,464^ HA encourages the adhesion of NPs via receptor targeting or integrin interaction.^465^ Following transcutaneous injection, a HA-NP generated by the self-package of HA-hydrophobic moiety conjugates could gather and focus on pro-inflammatory macrophages in the irritated dermis, reducing epidermal hyperplasia and inflaming reactions in rodent models of imiquimod and IL-23 induced skin inflammation.^466^ Scavenger receptors (SRs) are potential targets for inflammation along with oxidative damage due to their high affinity for glycoxidation end products, protein oxidation-derived compounds, and peroxidative lipid metabolites.^467^ The palmitic acid-modified bovine serum albumin has been created to possess outstanding SR-A targeting capacity, which would send anti-inflammatory drugs CLT to trigger macrophages in inflamed sites.^468^ Correspondingly, an amphiphilic polysaccharide was created by combining 5-cholanic acid to a dextran sulfate (DS) framework, which was preferentially taken up SR class A-regulated endocytosis. These DS NPs enable the targeted administration of MTX to stimulated macrophages, which are in charge of irritation and joint degeneration.^469^

Integrin-based NPs with issue specificity have great potential for efficacy in inflammation since integrins facilitate leukocyte aggregation in areas of irritation.^470^ Antheraea pernyi silk fibroin possesses a lot of arginine-glycine-aspartate (RGD) tripeptides, which could selectively attach to integrin receptors on the surface of colonic epithelial cells and macrophages in the irritated colon.^471–473^ The impaired colon epithelial lining was repaired by the Res-ApNP therapy, which polarized macrophages to the M2 state to minimize inflammation and the production of cytoplasmic ROS.^474^ In addition, following PEG fragmentation by collagenase IV, celastrol-loaded enzyme-responsive NPs (PRNPs) simultaneously target osteoclasts (OCs) and inflammatory macrophages generated from RA patients through contact with the RGD-v3 integrin. These PRNPs, which are made up of RGD transformed NPs (RNPs) coated with cleavable PEG chains, deliver CLTs to specifically cause apoptosis of OCs and macrophages in arthritic sites.^475^ Similarly, the secreted protein acidic and rich in cysteine (SPARC) and its ligand albumin is another receptor-ligand interaction for NP targeting. Based on the fact that SPARC is overproduced in the joint lubricating fluid of RA patients as well as animals with collagen-induced arthritis (CIA), MTX-loaded human serum albumin (MTX@HSA) NPs have been developed as biomimetic drug transport platforms for RA treatment. Combined fluorescence and MRI demonstrated larger collections and longer preservation of MTX@HSA NPs in inflammatory joints after intravenous infusion of chlorin e6-labeled MTX@HSA NPs into CIA rodents.^476^

There is a growing fascination with edible plant-derived exosome-like NPs, which are nanoscale particles created from edible plants (like ginger, grape, carrot, and broccoli) and have been shown to reach cells in the body via specific ligands.^477,478^ In a dextran sodium sulfate (DSS)-induced colitis model, oral grape exosome-mimetic NPs targeted intestinal epithelial cells (IECs) and macrophages, enhancing IEC viability, reducing TNF-α, IL-6, and IL-1 levels, and increasing IL-10 and IL-22 concentrations.^477^ Oral delivery of exosome-like NPs (PELNs) generated from portulaca oleracea L. (purslane, POL) specifically localized inflamed regions in a DSS-induced murine UC model.^479^ NPs generated from culinary plants offer an advantage of tackling restrictions associated with artificial NPs like possible toxicities and restricted industrial scale.^477^

Plenty of discoveries shed light on the utilization of NPs as a versatile tool for conducting thorough receptor/ligand interaction investigations and designing nanosize transport and treatment platforms.^480^ Nevertheless, no particular method will work in each case, and the selection of which ligand to use is entirely dependent on the application.

##### Cellular membrane-coated nanocarriers

Because of the inherent active-targeting capacity and superior biocompatibility, cells and associated extracellular vesicles are lately extensively used to build biomimetic techniques for the therapy of many diseases.^481–483^ Biomimetic camouflages made of cell membrane-coated NPs might lose their source cells’ operational capabilities while keeping just their surface characteristics.^483^ For the purpose of focusing on inflammation, biomimetic NPs could mimic several of the immune network’s normal inflaming reaction pathways.^484^ Immune cell membrane molecules may provide NPs with an array of benefits, such as longer systemic circulation, exceptional antigen recognition skills for improved targeting, stronger cell interplay, progressive drug discharge, and decreased in vivo cytotoxicity.^485^

The application of macrophage membrane-coated NPs offers a promising avenue for targeted therapy against inflammation. Macrophages have a key role in multiple aspects of AS’s degenerative process. Biomimetic NPs (MM/RAPNPs), which used macrophage membrane (MM), were designed by coating the exterior of rapamycin (RAP)-loaded PLGA copolymer NPs with MM, in order to mimic macrophage homing into arterial wall lesions. In vivo, the MM-coated NPs efficiently targeted and accumulated in atherosclerotic plaques.^486^ Similarly, biomimetic NPs (MM@Lips-SHP1i) are produced from MM covered SHP1i-carried liposome NPs. MM@Lips-SHP1i NPs would battle macrophages in the plaque site in vivo to interact with oxidized low-density lipoprotein and LPS, decrease the intake of fresh lipids by macrophages, and decrease the production of foam cells.^487^

Researchers also created biomimetic nanocarriers for targeted inflammation therapy inspired by natural PLTs. Because of its abrupt onset and narrow treatment window, acute IS presents a substantial obstacle for timely detection and intervention. Li et al. created a biomimetic NP based on PLT membrane envelopes stuffed with l-arginine and γ-Fe2O3 magnetic NPs (PAMNs) for thrombus-oriented administration of l-arginine and in situ generation of nitric oxide (NO), which was motivated by PLTs’ function in focusing on binding to the impaired blood vessel over thrombus development. Results show that with the supervision of an ambient magnetic field, the designed 200 nm PAMNs rapidly pinpoint IS-related sites and retain the inherent characteristics of the PLT membrane.^488^

The application of neutrophil membrane-coated NPs demonstrates the potential for targeted drug delivery in inflammation treatments. For precise medication administration during the therapy of RA, Yang et al. construct a biomimetic F127 polymer coiled around an altered neutrophil membrane ApoA-I mimetic peptide. The neutrophil membrane wrapping may offer the NP synovitis-orienting abilities, leading to migration to the articular fluid under the chemotactic actions of IL-8, owing to the abundance of molecular adhesives and chemotaxis receptors on neutrophils.^489^ As potential methods for gene silencing, ASOs have been used to treat human illness. For the specific administration of ASOs against microRNA-155 to the endothelium in atherosclerotic regions, Liu et al. developed a neutrophil membrane-covered zeolitic imidazolate framework-8 nanocarrier system. By engaging with the surface proteins of endothelium intercellular adhesion molecule-1 (ICAM-1) and neutrophil surface protein CD18, the neutrophil membrane can enhance plaque endothelial cell focusing.^490^ Inflammation-targeting biomimetic NPs are thought to have a promising future in developing nanomedicine.^484^

##### Stimuli-responsive drug delivery systems

The ability of stimulus-responsive materials to modulate drug transport in reaction to particular stimuli, including pH, enzyme concentrations, or redox gradients, has sparked a great deal of attention in recent years.^491^ Notably, stimuli-responsive NPs hold enormous potential for delivering adjuvants and antigens to specific immune cells, avoiding antigen deterioration and elimination, and boosting the intake of particular APCs, thus maintaining adaptive immune reactions and enhancing treatment for inflammation.^492^

According to investigations, elevated rates of metabolism in inflamed regions cause a steady rise in acidic metabolites and excessive oxidative strain, which are defined by low pH, ROS, and hypoxia.^493^ Healthy tissue has a pH of about 7.4, whereas inflamed tissue has a pH of about 6.4.^494^ In addition, irritated tissues have substantially higher ROS concentrations than healthy tissues. Scientists prefer pH and redox-responsive NPs because inflamed tissues have a special pH and redox milieu.^495^

When ROS levels in inflammatory sites rise, NPs with ROS-reactive devices unleash medicinal payloads at the appropriate time and location.^496^ Anti-rheumatic medication delivery over an extended period of time frequently results in noticeable side effects and patients’ failure to comply. Ni et al. created Dex-carried ROS-reactive NPs (Dex/Oxi-CD NPs) and FA altered Dex/Oxi-CD NPs (Dex/FA-Oxi-CD NPs) and confirmed their soothing activity in vitro and in vivo to selectively transport Dex to swollen joints.^497^ ALI is an inflammation condition linked to cytokine storm, which stimulates a number of ROS cascades and adversely affects patients. Utilizing an improved emulsion technique, Muhammad et al. created Dex-loaded ROS-responsive polymer NPs (PFTU@DEX NPs), which had an elevated packing content of DEX (11.61%). In ROS surroundings, DEX was liberated from the PFTU@DEX NPs more rapidly, which could effectively detoxify excess ROS in vivo.^498^ ROS-reactive drug administration platforms have remarkable performance in biomedical fields as their payload is exclusively delivered at inflamed areas marked by high ROS levels.^499,500^

pH-responsive NPs usually modify their drug discharge depending on the acidic milieu of inflammatory tissues. A precisely regulated one-step biomimetic mineralization technique was used to encapsulate insulin, GOx, and catalase (CAT) into ZIF-8 NPs, in which a powerful enzyme cascade complex (GOx/CAT group) acted as a tailored glucose-reactive component that can quickly catalyze glucose and produce gluconic acid to decrease the regional pH and efficiently devour the toxic derivative hydrogen peroxide, triggering the breakdown of pH-reactive ZIF-8 NPs for insulin discharge.^501^ In UC, NP-based medication transport platforms build up in the damaged epithelium of inflammatory colon mucosa. To achieve total drug protection in a gastric-like pH and precise transport of NPs to the colon, persistent medication-releasing PLGA NPs were produced and subsequently enveloped in pH-responsive Eudragit FS30D MPs.^502^ Because ischemia tissues exhibit a lower pH milieu than healthy ones, an acidity-responsive theranostic RAP-carried NP platform for ischemic brain tissues was developed. Accompanied by its swift dissolution in acidic conditions, treatment precision is enhanced.^503^ NPs with stimulation reactions can enhance drug concentration in infections, and are particularly attractive in inflammation precise treatment.^495^

Typically, patients undergo fingertip prick plasma glucose monitoring and insulin injections to keep up normoglycemia.^504^ Conventional techniques, on the other hand, not only lead to physical pain and trouble to patients, but also provide an elevated risk of complications because of erroneous insulin administration.^504^ As a result, the creation of a smart insulin administration device that adapts to ambient levels of glucose is critical.^505^ Amongst these, a frequently employed technique for designing glucose-reactive materials takes benefit of PBA and its derivatives’ capacity to interact transitorily with cis-diol groups.^506,507^ The glucose-sensitive insulin-liberating platform, AuNC-PBA-Ins assembly, was built with insulin transplanted on the exterior and enhanced the potency of insulin discharge in reaction to glucose level.^508^ Furthermore, stimuli-sensitive hydrogels (HGs) with regulated drug discharge profiles are an ongoing issue for enhanced medicinal applications. Ali et al. show that the elastic solid-like features of CS NP/poly(vinyl alcohol) hybrid hydrogels (CPHGs) are drastically diminished in low-pH and high-glucose settings. Under physiological settings, an in vitro drug release experiment revealed size-relied glucose-reactive drug delivery from the CPHGs (Fig. 5b).^509^

In terms of the roles of distinct tissues or organs, seeking distinct elements inside their milieu that impact the activity of NPs is also extremely novel. Colon-oriented oral medication delivery devices are attractive for the therapy of UC. In reaction to regional elevated cellulase action, two-layer Budesonide-carried SLNs with nanosized particle diameter and negative zeta activity demonstrated the desired selective drug release in the colon.^510^ Also, the in vitro unleashing studies demonstrated that CSO/Dex/LNPs were esterase-sensitive and would swiftly discharge the payload in synthetic intestinal fluid with esterase.^511^ The investigations emphasize novel drug transport techniques that provide precise regulation over drug discharge in reaction to niche conditions.

Endogenous cues concentrated in the illness microregion, including pH change, redox gradient, and particular enzymes, facilitate autonomous drug administration via activating the sensitive elements in the nano-assembly. Because of their excellent specificity, the delivered medicine in a form of endogenous stimuli-sensitive nanocomplex might accumulate at the intended areas,^512^ resulting in fewer adverse reactions and an increased therapeutic index.^513^

##### Inhalation delivery

Despite persistent attempts to improve chronic asthma therapy, symptomatic medications remain the sole choice for managing this common and painful condition. Inhalation is the most basic and effective means of administering drugs to the lungs.

The lung serves as a “center” for autoimmunity, via which autoreactive T cells travel before reaching illness locations. According to Saito et al., focusing on lung APCs with antigen-carried poly(lactide-co-glycolide) (PLG) particles alters lung CD4^+^ T cells, allowing mice to tolerate experimental autoimmune EAE. Intratracheally administered particles were connected with lung APCs and reduced costimulatory component synthesis on the APCs, inhibiting CD4^+^ T cell growth and decreasing their number in the CNS while boosting it in the lung.^514^ Small interfering RNA (siRNA), which possesses intrinsic and selective mRNA cleaving ability, has been hailed as a potential therapy for lowering the exacerbation rate of asthma by preventing airway epithelial cells (AECs) from expressing and releasing inflammation-promoting cytokines. In order to locate the intercellular ICAM-1 receptors on the apical portion of AECs, Zhang et al. developed new inhaled LNPs. With the help of this transport platform, siRNA will be delivered to AECs more effectively, reducing the production of inflammation-promoting cytokines and the accompanying symptoms.^515^

As an essential strategy for managing illnesses like asthma or pulmonary irritation, inhalable NPs provide a non-intrusive and effective means to administer soothing drugs precisely to the respiratory system.

#### Enhanced therapeutic agents

The present ways of treating inflammation mostly concentrate on pain alleviation and inflammation control. For example, nonsteroidal anti-inflammatory medicines, DMARDs, glucocorticoids, and biological substances are the primary drugs employed to manage RA.^516,517^ In order to attain optimal accumulations at regions of inflammatory and pathogenic states, oral or systemic delivery frequently requires larger dose levels and/or frequency, which exacerbates the unfavorable effects.^518,519^ Therefore, designing nanocarriers that can stably and slowly release drugs, enhance drug retention in the body, and even traverse biological barriers such as the BBB is of paramount importance.

##### Improved drug stability

Many drugs exhibit excellent efficacy against inflammation in vitro; however, their clinical application is limited due to their instability in the human body’s environment. NPs not only serve as carriers for these drugs, delivering them to the site of inflammation, but also protect their activity within the human body.

As a result of the poor pharmacokinetics, glucocorticoids have considerable adverse reactions, which make them tough to utilize in the therapy of RA. Nanomaterials have the potential for dispensing glucocorticoids, but the packaging of extremely crystalline and insufficiently water-soluble medicines leads to inadequate drug encapsulation and limited stability. The construction of 130 nm NPs formed entirely of dextrose palmitate and supported by phospholipids connected to PEG exhibit a negative zeta potential (55 mV), good capture efficacy, and durability for 21 days when stored at 4 °C. In a mouse collagen-triggered arthritis model, the increased therapy efficiency of the NPs was also revealed.^520^ In order to prevent broad exposure and immune suppression, the optimum anti-TNF treatment for IBD ought to transport the antibody precisely to the areas of intestinal irritation. The oral administration of antibodies is extremely difficult due to a number of obstacles, including limited membrane penetration and digestive enzymes in the GI tract.^521^ By assembling hydrogen bonding supramolecular NPs with TA and 1,2-distearoyl-sn-glycero-3-phosphoethanolamine-N-[methoxy(polyethylene glycol)-2000], Wang et al. develop a method for delivering antibody orally. Infliximab could be targeted to the region of intestine inflammation and shielded by NPs in the GI system without decomposing.^522^

NPs, when employed as drug carriers, exert a profound stabilizing effect by safeguarding the drugs’ chemical integrity within the complex milieu of the human body. This protective function ensures the maintenance of drug activity, overcoming the limitations posed by their inherent instability in vivo, thus enhancing their therapeutic potential for clinical applications in inflammation management.

##### Sustained drug release and improved internalization

Nanotechnology ensures precise drug delivery to inflamed regions for optimal therapeutic efficacy by achieving sustained drug release and improving cellular internalization.

Considering the physically enclosed payloads with drug explosive release and/or drug embedding, a novel prodrug technique is required to improve the construction of customizable nanotherapeutics. Zeng et al. developed a dendrimer-based drug delivery system with a benzoboroxole payload that is pH and glucose responsive. The dynamic interaction of benzoboroxole and glucose could efficiently modulate micelle disintegration and insulin secretion pace, hence stabilizing plasma glucose swings.^523^ Mai et al. synthesized tris(hydroxymethyl)aminomethane-modified bioadhesive NPs (Tris-BNPs). Tris molecules gradually disperse away as Tris-BNPs spread and permeate into the epidermis, disclosing the aldehyde groups of BNPs, which may attach to amine groups existing inside affected skin, resulting in lengthy regional preservation of BNPs in psoriatic cutaneous abnormalities (Fig. 5c).^524^ Uveitis frequently needs corticosteroid medication to avoid inflammation-associated ocular consequences. Nevertheless, utilizing traditional embedding technologies, it is challenging to pack extremely water-soluble medicines, including Dex sodium phosphate (DSP), and produce prolonged drug discharge. When carboxyl-terminated PLGA was employed as an electrical “bridge” between the PLGA and DSP, it was possible to encapsulate DSP into biodegradable NPs with a rather substantial drug load (6% w/w). DSP-Zn-NP subconjunctival infusion yielded measurable DSP concentrations in both the anterior and vitreous chambers of the eye for a minimum of 3 weeks.^525^

In addition to achieving sustained release, NPs also facilitate the internalization of drugs by cells. In comparison to the unbound drugs, retinoic acid against MS in 70 nm consistent-sized lipid nanocapsules, coupled with superparamagnetic iron oxide NPs, and altered with transferrin-receptor targeting peptide demonstrated a 3-fold improvement in uptake by endothelium.^526^ To treat the effects of UC, a CS-integrated poly(lactic-co-glycolic acid) NP platform was designed. The bioadhesive nanotechnology increased medication durability in the GI tract by increasing the efficacy of intestinal epithelium absorption.^527^ Polyamidoamine dendrimer is investigated as a nucleic acid transporter and has anti-inflammatory abilities. Histidine and arginine were concatenated to the main amines of polyamidoamine generation 2 (PG2) (yielding PG2HR) to increase DNA transport effectiveness. PG2HR transported pDNA into cells primarily via clathrin-independent endocytosis and partially via macropinocytosis. The gene transport effectiveness of PG2HR proved greater than that of PG2 or PG2 coupled with simply arginine (PG2R), which could be attributed to the pDNA/PG2HR complex’s better cell absorption and endosomal escape.^528^

NPs can exhibit good tissue penetration. Rachamalla et al. produced novel lithocholic acid analogs that would naturally generate LNPs in the aquatic environment, infiltrate the skin, enter the deeper dermal layers, and exhibit soothing actions against psoriasis-like persistent skin irritations.^529^ Similarly, deep eutectic solvents (DES) were shown to improve the skin permeability of stiff NPs. Li et al. described a practical and straightforward transdermal administration approach for topical RA therapy employing mesoporous silica NPs embedded in DES hydrogels. The hydrogel solvent pushed the stiff NPs over the cutaneous barrier in a non-invasive way following administration to the skin, causing prolonged infiltration and collection of MSNs at SC inflammatory areas.^530^ The Fc/FcRn transportation route was designed as an approach for increasing medication delivery across the epithelium.^531^ The Fc on the exterior of the NPs-fedratinib engaged with the FcRn displayed on the epithelium in the lungs, circumventing the lysosome and supporting NPs passing the epithelial barrier via transcytosis and aggregating at the lung parenchyma with an inflamed milieu.^532^

Sustained release and enhanced internalization of NPs for anti-inflammatory drugs represent a promising avenue in inflammation treatment. These NPs offer controlled drug delivery, ensuring a prolonged therapeutic effect, while their enhanced internalization into target cells enhances drug efficacy.

##### Extended drug retention capabilities

NPs with longer drug preservation properties avoid elimination mechanisms while maintaining medical effectiveness. Attempts to prolong NP retention time in vivo sparked numerous tactics in particle interface remodeling to avoid macrophage absorption and systemic elimination.^533^

The enhanced lymph angiogenesis and lymphatic circulation in arthritic locations have hastened the removal of NPs in inflammatory joints. Dex-KLVFF-PSA (DKPNPs) could maintain a steady nanoscale shape in normal conditions, and the PSA shell might equip DKPNPs with extended circulation and effective localization to arthritis areas. In inflammatory joints, acidic pH-induced Dex dispersion or macrophage-induced specialized interaction with PSA could cause DKPNPs to reassemble from NPs to nanofibers, resulting in decreased lymph removal and extended potency.^534^ At present, sinomenine hydrochloride (SIN) formulations, a natural DMARD, are utilized to treat RA; nevertheless, SIN’s potency is severely hampered by its brief half-life, poor bioavailability, and dose-relied side effects. In vivo images revealed that the superior immune-escape characteristics of the biomimetic NPs based on Prussian blue NPs led to significantly higher circulatory half-life and drug concentrations at arthritic regions of arthritis rodents.^535^ Glycyrrhizic acid (GA) has powerful soothing properties and suppresses COX-2 via inhibiting 11-hydroxysteroid dehydrogenase type II.^536^ GA has poor oral bioavailability in humans and rodents, since it was observed that GA was not traceable in human circulation after 100–800 mg of GA was consumed orally. Aminocellulose-grafted-polycaprolactone covered gelatin NPs are used as a transporter to achieve higher treatment efficiency than free medicines in the therapy of RA by co-delivering of GA and budesonide.^537^

The development of an orally delivered medication delivery platform capable of extended preservation in the GI tract is a critical problem for the successful management of GI illnesses like IBD. Zhao et al. describe a bioadhesive fluid coacervate synthesizing via hydrogen bonds. The liquid NPs-based coacervate exhibits essential pH- and salt-independent structural steadiness and creates a physically adhesive covering on an extensive area of the intestines with a raised retention time of over 2 days.^538^ 6-shogaol, a soothing medication applicant, showed remarkable efficacy in a number of in vitro tests. Nevertheless, due to its quick degradation upon oral treatment, it has low bioavailability and imperceptible in vivo pharmacokinetics. Yang et al. developed a natural-lipid (NL) NP drug administration platform to enclose 6-shogaol and transport it to the desired therapeutic target (colon). When contrasted with natural 6-shogaol, the in vitro drug-release experiment demonstrated that NL-embedded 6-shogaol (6-S-NL) has a prolonged drug-release pattern.^539^

Gouty arthritis (GA) is an intractable metabolic illness that needs ongoing therapy with regular drug delivery multiple times each day. IL-1 inhibiting medicines, like IL-1 receptor antagonist (IL-1Ra), offer high treatment promise in clinical research of GA in contrast to non-specific small organic drugs. Yet, IL-1Ra’s utilization is severely constrained because of its brief half-life and restricted bioaccessibility. Zhang et al. use the noncovalent construction of a designed IL-1Ra chimeric protein to show a novel kind of nanomedicine preparation. Surprisingly, the NP complex has a biological availability 7 times greater compared to that of pure IL-1Ra a remarkably lengthy half-life of 27 h, prolonging the dosage period from a few hours to over 3 days. The construction of protein-tethered nanoplatforms presents novel possibilities for studying long-lasting and safer medicines for GA therapy.^540^

NPs excel in drug delivery by prolonging drug retention in the body, leading to enhanced therapy and reduced toxicity. This attribute not only improves treatment outcomes but also lowers the risk of adverse effects.

##### Synergy effect

Inflamed disorders such as AS, RA, and psoriasis are long-term disorders caused by a variety of parameters. When seeking to control a single pathogenic variable, standard therapies frequently fail to provide beneficial clinical outcomes.^541^ NP injection is one of the most appealing techniques for harnessing the synergy of many co-packaged compounds for an identical target (Fig. 5d).^542^

The prevalence of UC is related to many factors.^543^ Macrophages are acknowledged as significant target cells in UC therapy.^544^ Meanwhile, macrophage mitochondria, which are crucial biological components, were regarded as critical facilitators in the therapy of UC.^545^ A novel class of sequence-targeted astaxanthin NPs for the therapy of UC has been constructed and it was exciting to observe how effectively the created sequence-targeted astaxanthin NPs were able to hit macrophages and mitochondria.^546^ During the course of UC medication, pro-resolving elements (such as anti-TNF antibodies), which are vital to colonic epithelium restoration were downregulated. Elevated levels of substances like IL-22 throughout the medicinal suppression of TNF-α were believed to aid in the healing process of intestinal irritation. Gal-siTNF-NP/IL-22-encapsulated hydrogel may be used to orient the inflammatory colon and simultaneously deliver siTNF and IL-22 to enhance the outcomes of either treatment.^547^

Psoriasis is defined by several inflammatory pathways that communicate with each other that are specifically linked to aberrant interaction between immune cells and keratinocytes. In order to implement integrated multiple targeted treatments for psoriasis, an NP-based hydrogel was developed by incorporating MTX into ZnO/Ag. ZnO-loaded mesoporous spheres were employed as antioxidant NPs and drug transporters in this hybrid hydrogel. AgNP-anchored ZnO NPs (ZnO/Ag) in an appropriate quantity were modified with an innate immunological regulation capability outcomes of either treatment outcomes of either treatment.^548^ Furthermore, for a coupled chemo-photodynamic treatment for psoriasis, Wang et al. created CS/hyaluronan nanogels to co-carry MTX and 5-aminoleavulinic acid (ALA), or MTX-ALA NGs. NGs increased cellular intake (*p* < 0.001), protoporphyrin IX transformation (*p* < 0.001), and ROS formation (3.93-fold), which together had a synergistic inhibitory effect on cell growth and induced apoptosis in HaCaT cells exposed to LPS with a 78.6% apoptosis ratio.^549^

In order to operate as an anti-atherosclerotic drug and a nanoscale delivery system with self-transportation capabilities, Wang et al. created a hydrophilic-lipophilic complex of low molecular weight heparin and unsaturated fatty acid (LMWH-uFA). Practically, the hydrophilic portion, LMWH, inhibited premature vascular irritation by preventing monocyte adhesion, and the hydrophobic section, uFA, might control plasma lipid levels. RAP, a soothing medication, was enclosed in the micellar core, improving its dissolution in water, and worked in tandem with LMWH to interrupt the vascular irritation pathway at P-selectin. RAP-carried NPs dramatically decreased the plaque size and demonstrated excellent therapeutic benefits in an AS murine model.^541^

By utilizing their potential to combine several therapeutic properties, NPs demonstrate great capacity in dealing with inflammation. The comprehensive strategy has the possibility to improve the therapy for long-term inflammatory diseases.

##### Crossing the BBB

It is still challenging to convert CNS-focused treatments into better clinical results. The BBB, which is undoubtedly the most strictly guarded barrier in the human body and consistently blocks the majority of therapies, is primarily to blame for this. New approaches are becoming possible by developments in the engineering of nanostructures and their use in biomedicine, which may help us better comprehend and manage neurologic illnesses (Fig. 5e).^550^

The most efficient way to transfer NPs to the brain while navigating the BBB is by receptor-regulated shipping, which works at the receptor-ligand interaction zone.^551,552^ A transferrin-receptor interacting peptide was added to a tiny lipid nanocarrier harboring RA and superparamagnetic iron oxide NPs. The alteration enhanced its capacity to engage with cerebral cells, leading to a five-fold improvement in its efficiency to the BBB and a three-fold improvement in endothelial cell intake.^526^ The transferrin receptor 1 (TfR1) on the membrane of cerebral endothelium could be recognized and bound by heavy chain ferritin (HFn) NPs. As a result, HFn NPs would effectively permeate the BBB via TfR1 without any extra alterations.^553^

Taking a ride with immune cells is an ingenious approach to assist drugs pass the BBB as several immune cells voluntarily travel to the brain when inflammation arises. Blood-borne Th17 cells migrate through the BBB and settle in the MS milieu. This further results in a massive influx of Th17 cells into the brain and an escalation of localized inflammation. Therefore, employing a “Trojan horse” strategy, Th17 cells could be used as powerful cell carriers for cascade-based medicinal drug trans BBB.^500^ In cerebral ischemia, neutrophils travel from the bloodstream and cross the BBB and gather in vast numbers at the region of irritation. The peptide cinnamyl-F-(D)L-F-(D)L-F, which may selectively attach to the formyl peptide receptor (FPR), is employed to modify a nanocomplex interface as the membrane of neutrophils includes FPR. Following intravenous administration, the synthetic NPs hitched a ride with neutrophils to increase aggregation at the inflamed location of brain ischemia, which was accomplished by successfully adhering to the membrane of neutrophils in peripheral circulation via FPR.^554^

Furthermore, some NPs, due to their extremely small size, can more readily traverse the BBB. Selenium quantum dots (SeQDs) are incredibly tiny and have a rapid BBB penetration rate. According to the research results, SeQDs exhibit broad-spectrum antioxidant properties, high radical quenching capability, and protection of cells from oxidative load. According to in vivo studies, which show that SeQDs can constantly aggregate in the brain following speedy crossing of the BBB, SeQDs can swiftly relieve AD, dramatically enhance the memory damage of AD rodents, and enhance their cognitive and memory capacity.^555^

Nanostructures make it easier to create drug transporters that may safely cross the BBB in situations involving inflammation within the CNS, opening up new treatment options for illnesses that were previously difficult to manage.

#### Inflammatory modulation effect of NPs

The mechanisms by which nanotechnology alleviates inflammation primarily involve two aspects: the clearance of inflammatory markers such as nucleic acids, ROS, and cytokines; and the regulation of the immune response through modulation of immune cells.

##### Modulation of inflammatory factors

In the realm of inflammation, nanotechnology offers a multifaceted approach to modulate inflammatory factors, including nucleic acids, ROS, and cytokines, effectively controlling the inflammatory response at various levels.

The excess of free DNA molecules in lesions is considered to be a pathogenic factor in many diseases, the mechanism of which may be that cell-free DNA further triggers chronic inflammation. Cutaneous cationic polymeric NPs were proven to be an efficient treatment approach for addressing psoriasis via capturing cell-free DNA (cfDNA). Yet, electropositive cfDNA scavengers pose a serious threat to organs once they cross the skin barrier and enter the bloodstream. A variety of poly(2-(dimethylamino)ethyl methacrylate) (PDMA) grafted hairy silica particles (cSPs) with variable PDMA length and dimension are employed to eliminate cfDNA in the dermis for improved transition to clinical deployment.^556^ When cfDNA from injured or destroyed cells interacts with LL37, it becomes an immune reaction enhancer that aggravates psoriasis. According to Liang et al., cationic NPs effectively battle for DNA from the DNA-LL37 immunocomplex and prevent DNA-LL37-triggered cell stimulation.^557^

Hypoxia and oxidative strain are two critical metabolic elements in the emergence of numerous inflammations. Various research has demonstrated that oxidative load is a vital factor in AS development.^558^ Gao et al. showed that gadolinium doping of CeO_2_ (Gd/CeO_2_) nanozymes produced outstanding results for anti-AS as efficient ROS eliminators. It was discovered that the chemical modification of Gd increased the exterior fraction of Ce^3+^ in the nanozymes, improving their general capacity to scavenge ROS.^559^ Yuan et al. described versatile NP-based therapy for IS generated from a bioactive oligosaccharide substance (TPCD) created by covalently combining a radical-eliminating substance (Tempol) and a hydrogen-peroxide-clearing component of cyclodextrin-modified phenylboronic acid pinacol ester.^10^ More significantly, a redox process transforming ROS into O2 might simultaneously reduce oxidative burden and hypoxia, regulating the inflamed milieu.^560^

Elevated inflammation-promoting cytokines play a key role in the development of numerous inflammations. In RA, there is often an overproduction of inflammatory cytokines including IL-1, IL-6, and TNF-α, which would be elevated by the mitogen-activated protein kinase (MAPK)/extracellular signal-regulated kinase (ERK), NF-κB, and activator protein-1 (AP-1) signaling cascades. It was found that CQDs obtained from herbal medicines greatly reduced swelling signs and impeded the production of associated inflammation cytokines (IL-1, IL-6, and TNF-α), demonstrating exceptional anti-inflammation efficacy.^561^ Liu et al. suggested HIF-1 siRNA-carried calcium phosphate NPs enclosed in high-density lipoprotein-modified with apolipoprotein E3 (HIF-CaP-rHDL) as a treatment for RA in order to suppress the levels of HIF-1, NF-κB, and MAPK. By blocking these pro-inflammatory signaling pathways and attenuating cytokines including TNF-α, IL-1β, and IL-6, HIF-CaP-rHDL showed promise as a targeted remedy for RA.^562^ Persistent neurological inflammation caused by microglia is assumed to start in the early stages of AD and is essential to the etiology of the disease. Oxytocin (OT)-carried, angiopep-2-modified CS nanogels (AOC NGs) were developed for the prevention of AD through the suppression of intrinsic inflammatory reaction. The NPs efficiently suppress microglial engagement and lower pro-inflammatory cytokine (TNF-α and IL-1β) amounts by suppressing the ERK/p38 MAPK and COX-2/iNOS NF-κB signaling cascades.^563^ Nano-curcumin reduces inflammation and prevents oxidative stress in the treatment of diabetic depressed patients. Among the clinical trial, a significant fall was found in the mean score of anxiety in the nano-curcumin group (from 22.4 [4.03] to 20.6 [3.4]).^564^

##### Immune response modulation

In addition to regulating inflammatory factors, NPs can control inflammation by directly modulating the immune response. Specifically, NPs can regulate the polarization of macrophages (Fig. 5f), promote immune cell tolerance to self-antigens, target immune checkpoints, promote the conversion of Th17 cells to regulatory T cells (Tregs) cells, regulate pro-inflammatory monocytes, neutrophils, and DCs, as well as antagonize TLR in macrophages, thereby achieving a favorable therapeutic effect.

An initial event that triggers tissue deterioration and malfunction is inflammation brought on by autoimmune disorders and persistent damage. Pro-inflammatory monocytes move across the body and infiltrate irritated tissue. Monocytes play a vital part in the progression of MS, particularly because most magnified pro-inflammatory monocytes penetrate the BBB, causing neuron damage and recruiting more immune cells to penetrate the CNS. Monocytes exclusively and effectively absorbed curcumin-carried high-density lipoprotein-emulating peptide-phospholipid scaffolds (Cur-HPPS) via the SR class B type I receptor. By preventing pro-inflammatory monocytes from crossing the BBB in EAE mice, limiting microglia multiplication, and preventing the penetration of other effector immune cells, this treatment decreased EAE morbidity from 100% to 30%.^565^

Macrophages, which show exceptional variety and polarization, are crucial immune cells in innate immunity. In pathological situations, additional macrophages, besides the local ones, migrate to the sick regions and polarize into different phenotypes (mostly M1 and M2) in response to diverse variables in the microenvironment, thus performing varied activities and functions. Generally, macrophage polarization might be separated into two forms: classically engaged M1 and optionally induced M2.^566^ M1 macrophages primarily present antigens and eliminate harmful microbes and are inflammation-promoting, whereas M2 macrophages have roles that include suppressing inflammation, supporting tissue reconstruction, angiogenesis, and immune control.^567,568^ Research involving the adoptive transplant of M2 macrophages has shown that the tactic might successfully exacerbate allergic airway inflammation in rodents.^569,570^ Pei et al. investigated the exosomal membranes from M2 macrophages covered with biomimetic NPs (EM-PLGA@Dnmt3aossmart silencer). It was impressive that the EM-PLGA@Dnmt3aossmart silencer efficiently suppressed M2 macrophage polarization in AA, and the biomimetic NPs effectively collected in the lungs, resulting in gene silencing, along with a decrease in the level of inflammation cell invasion of the airway.^144^ From beginning to end, AS entails persistent macrophage activation. Triggering outstanding anti-AS reactions in ApoE-/- mice after intraperitoneal administration, the treatment of kaempferol-macrophage-biomimetic KPF shipping complex (KPF@MM-NPs) provided a vital drop in developing macrophage inflammation along with a decrease of major inflammatory cytokines and re-polarization M1 to M2 phenotype, which was linked to the inhibition of the ROS/NF-κB signaling networks.^571^

The etiology of ALI is greatly assisted by the stimulation of the TLR in macrophages. The proton pump inhibitors (PPIs) and the peptide-covered AuNPs-based TLR antagonists exhibit comparable modes of action, according to transcriptome sequencing and Connectivity Map analyses. The endosomal TLR cascade and inflammation reactions in macrophages and hematological mononuclear populations are proven to be inhibited by PPIs (including omeprazole), and these PPIs also demonstrate soothing effect in a murine model of ALI triggered by LPS. Omeprazole is subsequently converted into nanoscale using liposomes to improve its capacity for attacking macrophages and medicinal effectiveness in vivo.^572^ Moreover, P12, a novel soothing nanodrug composed of hexapeptides and AuNPs, could reduce the inflammation reactions regulated by TLR in macrophages.^573^

Antibodies produced in response to a specific autoantigen are the main driver of pathologic inflammation in several autoimmune disorders, indicating that therapies to promote tolerance to the autoantigen may be beneficial. Recombinant NPs have been created that cause both T cells and B cells to become tolerant. The RAP-carried PLGA core of the NPs, which stimulates the growth of Tregs, is encased in a lipid membrane. The lipid surface exhibits the antigen protein and a ligand of the B cell inhibitory co-receptor CD22, which work synergistically to prevent B cells from getting activated upon identifying the antigen.^8^ The optimal treatment for type 1 diabetes (T1D) is the recovery of immunological tolerance to cellular antigens since T1D is induced by the death of pancreatic beta cells by autoantigen-selective immunity CD4^+^ and CD8^+^ T lymphocytes. The homologous diabetogenic peptides could be exterior linked with or encapsulated in carboxylated 500 nm decomposable PLG NPs, which can quickly and effectively reestablish tolerance in NOD.^574^ In order to manage an MS model, PLG NPs coupled with pathogenic antigens were used to establish antigen-particular tolerance. T cells obtained from mice vaccinated against myelin proteolipid protein (PLP139-151) were cultivated together with APCs that were given PLP139-151-combined NPs, resulting in decreased T cell proliferation, elevated T cell death, and enhanced soothing reaction.^575^

Immune checkpoint proteins regulate the evolution and management of autoimmune disorders via regulating immune reactions. Recombinant murine PD-L1 (rmPD-L1) was utilized to alleviate multi-organ inflammation by regulating T cell response. The PD-1-displaying CD4^+^ and CD8^+^ T cells in the splenocytes were successfully directed by immunosuppressive composite NP therapy.^576^ Changes in the proportion of effector to regulatory CD4^+^ T cells are a hallmark of the possibly deadly autoimmune illness SLE. Immune checkpoints PD-1 and TIGIT have been shown to be upregulated in pathologic CD4^+^ T cells as the illness progressed, while their ligands PD-L1 and CD155 were downregulated. Guo et al. developed Dex-carried IFN-treated MHC class I defective tumor membrane-covered NPs using biomimetic nanomaterials in order to properly exploit the immunosuppressive ability of malignant cells for the therapy of SLE.^577^

The pathophysiology of the majority of prevalent autoimmune disorders, including psoriasis, RA, IBD, and MS, has been linked to Th17 cells.^578^ Furthermore, following medicinal immunodepletion, Th cell restoration rates best predict long-term effectiveness.^579^ The onset and progression of MS are intimately tied to the Th17/Treg imbalance, and the transformation of Th17 cells into Treg cells might assist in the reduction of irritation, offering a therapy for MS. When ROS are produced, a transformation inducer termed (aminooxy)-acetic acid is regionally liberated and then absorbed by Th17 cells. It is shown that Th17 cells can phenotypically transdifferentiate in situ into anti-inflammatory Treg cells utilizing the Trojan horse recombinant approach.^500^

A current focus of autoimmune therapeutics is DCs, which have a significant impact on the initiation of the adaptive immune reaction. Targeted metabolic modifier therapy that modifies DC signaling to polarize T cells suggests potential benefits, which might be attained by designing substances with more durable release.^580^ Low Zn^2+^ concentrations in hypoxic DCs may induce immunogenic DCs (igDCs), which in turn sets off an aggressive T-cell response that accelerates the immunological development of RA. To convert igDCs into tolerogenic DCs (tDCs) and prevent further T cell stimulation, Qiao et al. developed ZnO_2_/Catalase@liposome-Mannose nanoparticles (ZnCM NPs), a DC-specific immune-modulating method. ZnCM NPs demonstrated pH-sensitive directed intracellular transport of Zn^2+^ and O_2_ to igDCs. Upon blocking OTUB1 deubiquitination, ZnCM NPs stimulated CCL5 breakdown through NF-κB signaling, which caused the igDC-tDC transition and subsequently inhibited CD4^+^ T-cell homeostasis.^581^

Nanotechnology orchestrates a comprehensive strategy for immune reaction regulation, including immune cell transformation and immune response management, which together promote an anti-inflammatory environment and accurate management of inflammation.

### Advanced diagnostic and imaging techniques

The remarkable biocompatibility, targeting potential, and regulated release potential of NPs have positive effects on inflammations. As a result, diagnosing these disorders using non-invasive imaging technology to track disease development and monitor immunomodulatory drugs may be conceivable.^582^

Fluorescence imaging, an innovative diagnostic tool, has piqued the interest of researchers owing to its outstanding selectivity and responsiveness.^583,584^ The second near-infrared spectrum (second near-infrared window(NIR-II), 1000–1700 nm) nanotheranostics may be accurately targeted to the location of inflammation while also performing diagnostic and therapeutic modes in a single nanocomplex. A proper UC diagnosis is essential for creating an effective therapeutic strategy. Following oral treatment, BM@EP (a pH/ROS dual-sensitive nanoplatform) sends the dyad (BOD-XT-DHM) into the intestines and discharges the dyad particles upon being induced by the alkalinity in colon, subsequently after being induced by overproduced H_2_O_2_ in the swollen colon, the boronated bond within the dyad is fractured and the chromophore escape for second NIR-II fluorescence and optoacoustic visualization for UC examination and recuperation assessment.^585^ Theranostic platforms that incorporate FI in the NIR-II and photothermal therapy (PTT) under secure laser fluence have enormous promise in preclinical study and clinical execution. Employing semiconducting polymer NPs (L1057 NPs), Yang et al. describe a theranostic platform for NIR-II FI and PTT under 980 nm laser irradiation, with low (25 mW/cm^2^) and high (720 mW/cm^2^) laser fluence, correspondingly. The great luminosity, coupled with outstanding durability and biocompatibility, enables real-time viewing of the entire body and cerebral vasculature high-clarity as well as identification of brain IS.^586^ The efficacy and precision of data collection are constrained by the low photoluminescence quantum yield (PLQY) of fluorescence probes in the NIR-II area, particularly in multimodal molecular visualization in vivo. Lanthanide-infused NPs with high PLQY and adjustable PL lifespan via multi-ion loading and core-shell construction are offered as a solution to this issue. The high PLQY NPs and custom-built fluorescence lifespan visualization platform may provide quick fluorescence lifespan mapping with a high signal-to-noise ratio, paving the way for multimodal molecular visualization of AS.^587^

MNPs are utilized in MRI to enhance the image contrast of selected regions. To improve proton relaxation and visualization, MNPs could be targeted into the tissue location.^588^ MNPs are nanocrystalline particulates that may be additionally modified with biocompatible coverings and ligands, and are regarded as a future MRI element. Before thrombosis is activated and larger plaques are ruptured, causing coronary artery crises and abrupt heart failure, AS is asymptomatic for decades. In order to potentially employ MRI to image clot formation on arterial plaques, Poon et al. developed mixed metal oxide-peptide amphiphile micelles (HMO-Ms), which incorporate an organic peptide amphiphile with fibrin-Interacting properties with an inorganic, magnetic iron oxide or manganese oxide center. MRI tests performed in vitro using hybrid NPs modified with CREKA showed that they were capable of targeting with a two-fold amplification of MR signals.^589^ At the level of cells and tissue levels, Gd/CeO_2_ nanozymes against AS effectively eliminate the damaging ROS. These NPs may also be used as T1-weighted MRI contrast agents, which can produce enough contrast to identify the plaque’s position throughout live imaging.^559^

CT is an X-ray-based clinical imaging technology that is frequently employed for non-intrusive GI tract (GIT) imaging. In the clinic, CT contrast media containing iodine and barium are employed for GIT imaging; nevertheless, IBD imaging is difficult because iodinated and barium-based CT agents are not particular for areas of irritation. The usage of dextran as a covering substance on cerium oxide NPs was expected to promote aggregation in IBD inflamed sites in a manner similar to other inflammatory disorders. Cerium oxide nanoparticles (Dex-CeNP) bound with dextran provided a potent CT contrast and collected in the colitis region. Dex-CeNP may potentially be utilized as a viable CT contrast material for imaging the GIT in patients with IBD while preventing oxidative harm.^590^

Metal NPs are broadly used in nanoprobes for the identification of numerous analytes, such as metal ions, nucleic acids, proteins, small biomolecules, and ROS, due to their distinct physicochemical characteristics. The extremely sensitive identification of fibroblast-like synoviocyte cells was made possible by a PEC biosensor constructed on ZnO nanorods/CH3NH3PbI3/nitrogen-modified CQDs nanocomplex.^591^ The concurrent non-intrusive actual time measurement of glucose and insulin in saliva was made possible by the creation of an electrochemical aptasensor on a screen-printed carbon electrode (SPCE). The sensing interface reacted uniformly to insulin in a spectrum of 0.05–15 nM with a sensitivity threshold of 0.85 nM and uniformly to glucose in the spectrum of 0.1–50 mM with the electrochemical signal measurement on SPCE.^592^ Clinical research has shown that the use of CD24-Au nanocomposite as a biomarker can detect stem cells in salivary gland tumors with good diagnostic and prognostic results (NCT04907422).^593^ In addition, advanced in vitro detection devices can reduce the burden on patients. A sensing device based on nanomaterials (organically functionalised CNTs and AuNPs) has been able to clearly distinguish AD from healthy states, PD from healthy states, and AD from PD states, by detecting exhaled gases, with a classification accuracy of 85, 78, and 15% (NCT01291550).^594^

Nanotechnology leverages the distinctive physical and chemical properties of NPs to revolutionize disease detection in inflammation, providing highly sensitive and accurate diagnostic tools.

## The limitations/challenges of nanotechnology application

**Fig. 6:**
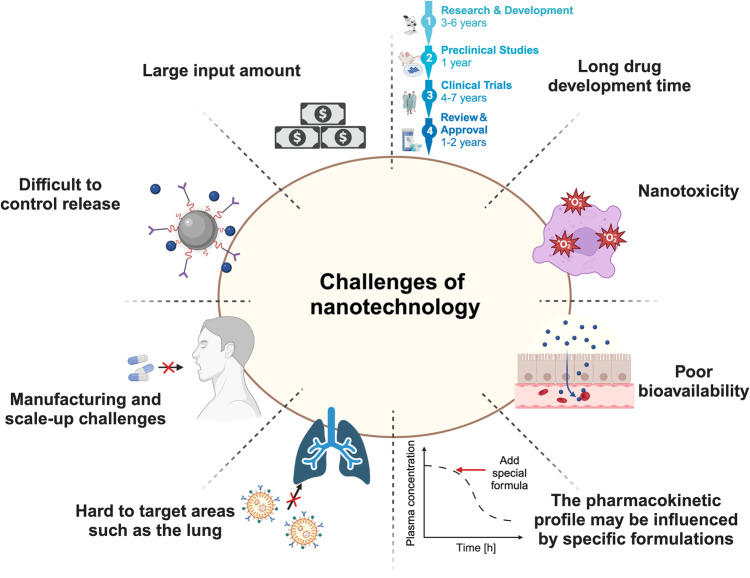
Challenges of nanotechnology. There are still various types of challenges in the development of nano-formulations. One is the challenge of drug delivery across different biological barriers. Systems such as the lung and gastrointestinal tract have their specific biological barriers that can prevent the passage of some NPs drug delivery systems, making it difficult to achieve specific aggregation. Second is the challenge of NPs prescription, characterization and preparation process. Prescribing specific nanomedicines, such as ivermectin with poor water solubility, carries challenges in development and preparation, and characterizing their physicochemical properties may also be difficult to ensure re-producibility due to differences in laboratory equipment and reagents. Third, there are cost and regulatory challenges. The time and money spent on the development of nanomedicines to market is incalculable, and regulatory issues are also a major challenge. Nanomedicines need to be gradually compared with reference drugs for efficacy, safety, and quality. NPs: nanoparticles

In recent years, numerous studies have focused on the development and utilization of nano-formulations as therapeutic approaches for infectious and inflammatory diseases. While nanomedicine offers unparalleled advantages over conventional medications, it also presents numerous limitations and challenges that must be addressed (Fig. 6).

### Toxicity and safety

The extensive applications of NPs have provided an innovative driving force for advancements in biomedicine. However, the associated issues of toxicity and safety cannot be overlooked. Research suggests that organs with rapid perfusion, such as the kidneys, liver, lungs, and spleen, are most susceptible to NP accumulation and subsequent damage.^187,595^ The primary mechanisms underlying this damage include the induction of oxidative stress, inflammation, genotoxicity, and cytotoxicity.^596,597^

There have been extensive reports on the adverse in vivo effects of metal NPs. Metal NPs may possess toxicity, leading to mitochondrial and DNA damage.^598–600^ They can also induce excessive ROS production, which is more likely to result in cellular dysfunction.^29^ AuNPs can directly penetrate kidney cells, producing toxic substances through cyanidation and oxidation.^601–604^ AgNPs, when inhaled, not only cause pulmonary toxicity but also exhibit dermatological toxicity upon local application. If internalized into sperm, AgNPs can lead to fertilization difficulty and impaired embryo development.^605,606^ Furthermore, carbon-based NPs, polymer NPs, and others have been widely reported to exhibit toxicity. CNTs have been demonstrated to induce inflammation, pleural damage, and even lead to mesothelioma originating from the pleura.^607^ Studies have found that MWCNT can induce lung inflammation following pulmonary exposure,^608^ and exacerbate myocardial ischemia-reperfusion injury.^609^ It was later discovered that this was due to MWCNT-provoked serum bioactivity leading to endothelial inflammation and barrier disruption.^610^ Reports suggest that PEG-functionalized IFN-α can induce allergic reactions and rashes in patients undergoing treatment for chronic hepatitis C.^611^ Oligomers of PLA NPs mediate the inactivation of matrix metalloproteinase 12, resulting in intestinal damage and acute inflammation.^612^

It is essential to emphasize that the long-term in vivo toxicity of NPs is of great importance because they can interact with the organs, tissues, cells, and macromolecules of individuals suffering from chronic inflammatory diseases. As NPs exhibit different dispersion pathways from their microscale counterparts due to differences in their physicochemical properties, they may accumulate in the liver or even traverse the BBB to reach the brain.^613,614^ It has been demonstrated that many nanocarriers can negatively affect cell membrane integrity and shape, damage lysosomes, lead to misfolded proteins, promote protein oxidative damage in the Golgi apparatus, and even enter the cell nucleus, causing alterations in DNA.^615,616^ Inadvertent inhalation of NPs can result in pulmonary toxicity, where NPs migrate from lung alveolar cells through internalization to various organs, including the heart, liver, and spleen.^616–618^

So, what optimization measures can we take to avoid the potential toxicity of NPs? Several studies have found that particle size is a crucial factor affecting nanotoxicity.^619,620^ Therefore, by controlling the particle size and morphology of NPs, their toxicity can be reduced, thereby regulating their biodistribution, biodegradability, and cellular uptake capabilities. In addition, high surface charge of NPs may lead to equivalent nanotoxicity.^621–624^ Cationic AuNPs are more toxic than neutral ones, as they can disrupt cell morphology and induce more pronounced mitochondrial stress.^625^ Therefore, nanotoxicity can also be reduced by altering the NP’s surface charge. Furthermore, in the field of nanotoxicology, surface area is widely regarded as a crucial determinant of nanotoxicity. However, the hemolytic response triggered by quartz particles is related to the generation of H_2_O_2_ on the particle surface, suggesting that the pulmonary toxicity of quartz NPs is not only related to surface area but also to surface activity. In fact, surface modification and functionalization can alter surface properties and interaction modes, thereby reducing toxicity to organisms. Chakravarty et al. reported that CNTs can be functionalized to enhance biodegradability and reduce toxicity.^33^ Yu et al. reported that GSH-coated fluorescent AuNPs can improve kidney clearance efficiency and rate.^626^ Ge et al. demonstrated that PEGylation and lanthanide (Gd^3+^ and Yb^3+^) modification reduces the in vivo and in vitro toxicity of AuNPs, enabling their application in in vivo imaging and PTT in tumor-bearing mice.^627^

Therefore, gaining an in-depth understanding of and shedding light on the linkage between nanotoxicity and its physical and chemical characteristics (e.g., material structure, surface properties, shape, hydrophobicity, and aggregation state, etc.) may pave the way for optimized design of nanomaterials with enhanced biosafety in the future.

### Delivery and location

Nanoparticulate materials need to be able to be delivered precisely to the target location and achieve targeted action. However, there are various barriers to NPs in living organisms, such as blood circulation, clearance by the immune system, and cell membrane blockage, which may limit their delivery efficiency and targeting. Existing studies prove that the successful delivery of drug-carrying NPs to target organs and tissues is related to the surface conditions of the target organ and the route of drug delivery.^76,628–631^ However, systems such as the lungs and gastrointestinal (GI) tract have their unique biological barriers that hinder the passage of certain NP drug delivery systems, making it difficult to achieve specific accumulation. For instance, although oral delivery is the most widely used and highly accepted form of drug delivery, but acidic conditions and a large number of degradative enzymes in the GI tract system can restrict the passage of some drugs, rendering them inactive before being absorbed, which results in drugs with low stability and low bioavailability are not easily absorbed in the GI tract and have difficulty reaching therapeutic levels in the blood.^632^

While brain-oriented improvements or biomimetic methods may enhance the specific impact of NPs in the brain, the BBB remains a significant penetration barrier. Many NPs have not yet been able to reach inflammatory diseases in the CNS, including IS and Alzheimer’s disease (AD).^633^ Therefore, non-intrusive or slightly intrusive techniques ought to be researched more thoroughly. For instance, intranasal delivery is a patient-friendly mode of treatment and might be a workable strategy if cerebral drug levels through this method are increased.^550^ However, the intranasal amount for delivery is modest, and the motion of the cilia and mucus significantly inhibits drug entry into the brain.^634^ In addition, it is possible to transport drugs to the brain without passing through the BBB by using the intrathecal and retro-orbital pathways. The two ways, nevertheless, are each constrained by high injection procedures and exceedingly painful patients. Furthermore, certain NPs founded on stem cells or gene treatment are employed for extremely intrusive cerebral delivery to preserve the survival of stem cells and enhance the efficacy of RNA or DNA transfection. In light of the evidence, the best medicinal benefits may be obtained by selecting the proper distribution pathways, in accordance with the various action mechanisms of NPs and associated targeting strategies.^633^ In addition, pharmaceutical agents need to be researched to enhance the pharmacokinetic characteristics within the actual cerebral compartment. A more efficient approach is necessary since the sluggish rate of dispersion within the parenchyma seems to limit the distribution of CNS medications. An efficient method of aiding medication transport to and throughout the brain might be offered by outside directed magnetic targeting or possibly remotely controlled nanotechnology.^550^

Uncertainty surrounds NPs’ in vivo procedure. Similar to conventional small-molecule medications, more research needs to be done on how NPs work in vivo. But pharmacokinetic information of drugs is infrequently disclosed for NPs while it is required for approval of medications, which limits the development of NPs as clinical drugs. We can determine its absorption, dispersion, metabolism, and elimination thanks to that information. Furthermore, as NP-based medicines focus on cells in vivo, certain studies on cell pharmacokinetics may be utilized as guidelines.^635^ The study by Paunovska et al. proposed a feasible approach to link the structure of nanomaterials with in vivo delivery, using high-throughput in vivo studies to investigate the delivery behavior of NPs targeting cells within the body,^636^ which provides a good lesson for us to study the behavior of localization and detection of nanomedicines in vivo.

Inefficient distribution of the active ingredient of the drug at the targeted location also limits the delivery of nanodrugs. For instance, due to the highly heterogeneous distribution of the vascular system in solid tumors, their high permeability and lack of proper lymphatic drainage increase the barriers to drug delivery, resulting in large tumor areas that may be poorly perfused. Local therapeutic delivery systems can address this issue, yet fail to target metastatic or disseminated tumors.^637^ Besides, there are many studies that can demonstrate the ability of NPs to increase drug localization and drug distribution at the target site,^638,639^ but the localization of NPs in various cell types is unknown and still needs further study.^638,640,641^

Active targeting that relies on ligand-receptor interaction is seldom employed to control the immune response by directing immune cells. A class of immune cells, such as immunogenic and tolerogenic DCs, may have different characteristics based on various phenotypes, increasing the challenge of targeted targeting based on ligand-receptor interactions.^642^ The possibility for precise distribution has so far been demonstrated by mannose-binding receptors, Toll-like receptors, and FA receptors.^643^ There is existing literature demonstrating that mannosylated ferritin NPs can precisely target B-cell follicles.^644^ In order to find additional relevant receptors, greater focus ought to be paid to cascade and checkpoint investigations.^635^ In addition, given the intricate pathogenic processes of inflammatory diseases, NPs typically only target one or two of these processes and are unable to offer a total defense. Contrarily, combining various medicinal compounds into a single nanocarrier makes the NPs more complicated and hinders the clinical application of these NPs.^633^

### Stability and long-term effects

The stability of nanomaterials during preparation, storage and application is a critical concern. Various factors can impact the stability of NPs including preparation methods, environmental conditions such as temperature, humidity and light, as well as in vivo aspects like pH and enzyme activity.^645–647^ Alterations to the stability of the nanomaterials can lead to potential reductions in efficacy, heightened toxicity and other adverse outcomes. They may aggregate or degrade in the environment, thus affecting their performance and long-term effects. Although some nano-agents have shown good antiviral potential in vitro assays, in vivo assays suffer from poor stability, which leads to poor bioavailability. Ivermectin (IVM), for example, has potent antiviral activity against the Zika virus, but the development of its nanodrug is hampered not only by the poor water solubility properties, also because the pharmacokinetic profile of IVM may be influenced by specific formulations, thus altering plasma kinetics.^648,649^ Similarly, liposomes encapsulating lipophilic drugs are often manufactured in processes involving organic solvents, which can lead to drug leakage.^650^ In response, the drug formulation needs to be changed or the production process needs to be improved. Some NPs, although not exhibiting nanotoxicity in the short term, may also exhibit toxicity upon long-term exposure. It was found that after prolonged exposure (20 days) to iron oxide nanoclusters (IONC) coated with PEG, higher amounts of PEG-coated IONC were observed in the follicular centers of the white pulp of the mouse spleen, which not only led to cellular abnormalities in the splenic red pulp, but also led to a decrease in collagen levels.^651^ Previous studies have speculated about changes in splenocyte nucleic acid material due to the production of ROS using PEG-coated IONC.^652^

How can we address the reduced efficacy and increased toxicity resulting from deteriorating stability? Altering the NPs’ design or developing a stabilizer may ameliorate these concerns.^653,654^ Materials available for use as NP stabilizers consist of surfactants, silica, biomolecules, polymers, and metal shells.^655–658^ These materials assist in maintaining the structural integrity of NPs, preventing their aggregation or dissolution, and thus providing stabilization. These issues need to be examined by us in the future.

### Clinical research and application

While there is a considerable number of preclinical studies demonstrating the superior benefits of nanomaterials in the treatment of infectious and inflammatory diseases, not enough nanomedicines are currently in clinical research or on the market to meet the demand. The challenges that may exist in the short term are: our current understanding of the pathogenesis of infectious and inflammatory diseases is not sufficiently deep or does not allow us to cope with rapidly changing conditions; the differentiation between animal models and the realities of the human body poses a challenge;^659–662^ and the technology for clinical design and translation needs to be improved.^663–665^

There have been numerous instances of widespread viral outbreaks in recent times, but research on antiviral nano-agents remains largely focused on treating influenza, hepatitis, herpes, coronaviruses, and HIV infections.^666,667^ Most of the antiviral nanomedicines that have reached the clinical research stage are designed primarily as vaccines, without sufficient capability to respond quickly to new viral outbreaks such as recent emergence of monkeypox.^668^ In addition, Excessive mutation rates during microorganisms genome evolution could give rise to drug-resistant strains, greatly diminishing the therapeutic efficacy of existing drugs.^669,670^ While nanocarriers may lead to unsuitable combinations of drugs and harmful side effects, ultimately compromising patient adherence.^671^ The biocompatible nanomaterials or technologies can reduce patient compliance problems, such as LNPs,^672^ and microneedle technology.^672^

We need to be aware that the efficacy of nanomedicines in preclinical research does not ensure their usefulness in humans, despite the previously exhibited enormous potential.^673^ The significant discrepancies in pathobiology between human and animal models could be the cause of this mismatch. For example, unlike the pathophysiology in humans, animal models of brain ischemia were often generated using the suture-occluded technique or the photochemical approach.^633^ Furthermore, while arterial plaque deposits in patients form over years or even decades and are thus more complicated and varied, atherosclerotic plaques in animal models typically form in weeks.^674^ Furthermore, animal models are frequently genetically altered in ways that aren’t always applicable to people.^675^ One of the initial NPs in the creation of RA nanodrugs that reached the phase of clinical trials was the liposomes. The results of a latest randomized clinical trial show that therapy of RA outbursts with intravenous infusion of PEG-coated liposomal prednisolone is superior to intramuscular methylprednisolone.^676^ But after administering prednisolone in PEG liposomal form intravenously, liposomes present a specific risk of inducing hypersensitive events. Persistent allergic reactions may make treatment futile, especially in inflammatory disorders. Moreover, therapeutic dosage control is hard as a result of the difficulty in accurately quantifying local drug levels of NPs administered via intravenous injection.^677^

In spite of the fact that the choice of preclinical models can be diverse, there are several challenges to designing a viable patient-delivered technology such as safety, stability, scalability, and cost of production.^663,678^ The option of nanomaterials is an important influencing factor for clinical translation. Complex approval processes for new materials may prolong the time to clinical translation, as the safety and efficacy of NPs must undergo rigorous review.^679,680^ Criteria for review include NP characterization and toxicity, where characterization would include size, shape, surface charge, stability, etc. In addition, unlike the singularity of preclinical research, clinical translation requires the cooperation of multiple departments, such as R&D institutions, experimental centers, regulatory agencies, and financial support. It is only when these sectors work together that promising nanomedicines can reach the market and be applied to patients.^678,681,682^ Therefore, more standardized policies and regulations are needed to ensure the clinical translation of nanomedicines.

### Cost and production

Nanotechnology-based drugs, often associated with high costs, present challenges in clinical adoption.^683^ Key factors impacting drug pricing include manufacturing, scalability, and storage. To reduce costs, exploring methods to enhance production yields and alternative, cost-effective industrial processes may be necessary. At present, the two most popular methods for increasing the scalability of nanodrugs are microfluidic systems and 3D printing technologies.^684^ The production of nanodrugs has been revolutionized by microfluidic systems. By regulating minute fluid droplets or volumes with extreme accuracy, they make it possible to produce nanodrugs that are precise and controllable. In addition, by combining various production steps into a single system via microfluidic technology, fewer preparation procedures and shorter manufacturing times will be required.^685^ Also, incremental fabrication and 3D printing innovation are currently highlighted as strategies to reduce the price of nanodrugs while enhancing clinical application. Easy steps are used in this manufacturing procedure to enable economical, easily scalable output.^677,686^ It’s encouraging to note that there are now some nanotechnologies available on the market for virus detection. The team at Shanghai Jiao Tong University, led by Gu et al., has developed nano-magnetic beads as the core carriers for extracting virus nucleic acids. Under the influence of a magnetic field, a large number of these magnetic NPs can precisely capture the targeted entities, transport them to specified locations, and facilitate rapid testing. However, there is currently no literature reporting a method for achieving large-scale production of this technology. Cost-cutting is the key to accomplishing industrial-scale manufacture of NPs, but it is also crucial to create constant, greatly scalable, and reproducible technology for preparing batches of nanodrug with managed size, consistent layout, colloidal durability, etc., while concurrently assessing expenses and practicality.^687^

Intriguingly, the complexity of synthesizing NPs industrially differs. For the treatment of developed thrombotic thrombocytopenic purpura, cablivi was developed. Also, it was the sole nano-drug (Nanobody^®^) that had been proven to cure autoimmune illness.^688^ Although its function with ordinary NP was very distinct, it is simply an immunoglobulin segment generated by the cell. Nanobodies^®^, in contrast to traditional monoclonal antibodies, are substantially tiny (15 kDa). It has heightened solvency, enhanced durability, better and more targeted distribution due to its tiny size.^689^ The effective “manufacturing” of Nanobodies^®^ occurs in a variety of prokaryotic and eukaryotic cells.^690^ The authorized nano-drug was generally straightforward, lacking a target ligand, combination, or alteration. On the other hand, modern NPs study frequently employs exceedingly complex alterations to emphasize progression and uniqueness, which makes industrial manufacturing quite challenging. Nevertheless, as modified NPs are more effective than pure NPs, changes are still required for clinical efficiency. Novel additives for convenient alterations, new efficient ligands (such as tiny molecular aptamers), along with straightforward modifying procedures are also highly required.^635^

### Regulations and ethics

The application of NP materials also faces regulatory and ethical issues. Due to their unique nature and potential risks, regulatory frameworks need to be established to ensure their safety and compliance. Considering future clinical translation efforts, drugs containing NPs must be manufactured according to specifications.^626^ The efficacy, safety, and quality of nanomedicines need to be progressively compared with reference drugs to achieve equivalent therapeutic effects, but some nanomedicine structures are not fully characterized and in vivo activity depends on the manufacturing process.^691^ Approval criteria related to generic nanomedicines are also being considered, and bioequivalence of generic and innovative nanomedicines cannot be assumed by similar results observed in general pharmacokinetic and toxicity studies or by simple comparisons of drug product components, but rather by using disease models to respond to the pharmacology of nanomedicines and by continuously testing the safety of nanomedicines on clinical trials over time.

## Conclusion and perspective

With the advancement of biomedical science, the underlying mechanisms of many infectious and inflammatory diseases have been elucidated, enabling their prevention, diagnosis, and early treatment. The rapid development of nanotechnology has provided a versatile “Swiss Army knife” tool for combating infectious and inflammatory diseases. Nanomaterials can serve as adjuvants and delivery carriers for vaccines, enhancing immune responses and antigen immunogenicity, stimulating potent cellular and humoral immunity to prevent pathogens infection. Moreover, nanotechnology can directly exert anti-pathogenic and anti-inflammatory effects, acting as drug delivery systems to target medications to infection and symptom sites, achieving sustained and precise drug delivery. In addition, they can also be used as biosensors, imaging enhancers for the detection of pathogens and the diagnosis of inflammatory diseases. While challenges related to the application of nanomaterials, such as toxicity, biocompatibility, long-term safety, etc. require further research and resolution, they have already demonstrated great potential in various fields, providing powerful support for future research.

NPs-based vaccines, particularly LNP-mRNA vaccines, have made significant advancements in the field of infectious disease prevention and control. In comparison to traditional vaccines, LNP-mRNA vaccines offer three major advantages. Firstly, their production speed is exceptionally rapid. Once the genetic sequence of a pathogen is obtained, the vaccine development process can be completed swiftly. Moreover, for pathogens with rapid mutational capabilities, upgraded vaccines can be developed promptly to address new variants. Secondly, another remarkable advantage of mRNA vaccines is their safety. From a production perspective, the absence of the need for large-scale cultivation of live viruses eliminates the risk of live virus leakage during vaccine production. Furthermore, mRNA segments do not enter the cell nucleus, thus eliminating the potential risk of stable integration into the host cell’s genome. In addition, mRNA vaccines typically do not require additional adjuvants, reducing the risk of adverse reactions that may be caused by adjuvants. Thirdly, another major advantage of mRNA vaccines is their high efficacy. They can simultaneously stimulate both cellular and humoral immunity, providing stronger protection against pathogen invasion. Furthermore, mRNA vaccines exhibit excellent scalability, allowing for the combination of different antigens for a multi-purpose vaccine. For instance, different sequences of SARS-CoV-2 variants can be integrated to prevent a broader range of variants. Different pathogen target sequences can also be designed together to provide simultaneous protection against multiple infections. LNP-mRNA vaccines are primarily administered via intramuscular injection. However, inhaled and intranasal delivery are considered to elicit IgA antibodies faster to block the spread of respiratory infectious diseases. This is also a potential direction for the future development of mRNA vaccines. Notably, LNP-mRNA vaccines have also demonstrated potential in cancer immunotherapy,^692,693^ prevention or treatment of allergies and autoimmune diseases,^694–697^ and even gene replacement therapy for rare genetic diseases.^698^ It should be highlighted that other nanovaccine platforms have shown significant potential in preventing infectious diseases like SARS-CoV-2. For instance, self-assembling NP vaccines, including the conditionally approved NVX-CoV2373 and the emergency use authorized (EUA) Coviccine (WestVac Biopharma) for SARS-CoV-2. The highly promising I53-50 self-assembling NP delivery system, has shown promising results in the research of vaccines for SARS-CoV-2, RSV, HIV, Lassa virus, and ECF.^219,221,222,224,699,700^ Currently, diverse nanovaccine technologies are under active development. The key to future advancement lies in expanding the range of vaccine development, strengthening international cooperation and coordination, and especially assisting developing countries in establishing comprehensive healthcare systems and training more professional technical personnel.

Nanotechnology has made some progress in the treatment of infectious and inflammatory diseases, such as the three-dimensional nanostructures against malaria,^437^ and the plant-derived exosome-like NPs for UC treatment.^479^ However, there is still a need to enhance the target specificity of nanomaterials for precision medicine. In recent years, nanomaterials have advanced rapidly in cancer therapy. Broadly speaking, cancer can also be considered a chronic inflammatory disease, so the targeting strategies of NPs in cancer treatment are worth considering for infectious and inflammatory diseases. The complexity of the tumor microenvironment poses higher demands on delivery systems, and also provides opportunities for designing and validating various advanced nanocarriers. For instance, Song et al. proposed a novel drug delivery approach that could target bacteria existing in tumor sites, specifically addressing the unique bacteria-rich tumor microenvironment.^701^ Some obligate or facultative anaerobic bacteria can selectively accumulate in tumor tissues, and also serve as tumor-targeting carriers.^702–705^ Luo et al. found that surface-modified lanthanide NPs can be readily adjusted to achieve tumor targeting through ligand modifications.^706^ In addition, with the establishment of NP transport models based on micrometastasis pathophysiology, personalized treatment based on the unique biology of patient micro-metastases may become a reality.^707^ Therefore, the advancements of nanotechnology in cancer treatment can provide inspiration and insights for targeted therapy of infectious and inflammatory diseases, paving the way for the design of more targeted and personalized treatment strategies.

A variety of nanomaterials have been developed for the detection and diagnosis of infectious and inflammatory diseases, including metal NPs, carbon-based nanomaterials, polymer nanostructures, magnetic nanomaterials, QDs, nanowires, or nanomembrane structures.^708–711^ Based on different detection principles, they can be mainly divided into in vitro chemical reaction detection, sensor technology, and imaging technology. However, the stability, toxicity, and specificity of nanoparticles themselves are key limiting factors in their detection applications. These limitations can be improved through combination strategies or surface modification.^712–714^ For example, the combination of AuNPs with fluorescence detection can expand the detection range and improve specificity.^715^ A dual-mode detection method (colorimetric-fluorescence) combining PCR has been designed to achieve rapid and sensitive detection of Salmonella.^716^ CRISPR-based detection systems, such as SHERLOCK, DETECTR, and STOPCovid, are becoming important emerging tools for viral detection due to their speed, specificity, and ease of use.^717^ It should be noted that the SHERLOCK test kit and DETECTR test kit have received EUA from the U.S. FDA and can be used for the detection of the SARS-CoV-2 virus. The latest generation of SHERLOCK technology enables faster (30–60 min) and more sensitive (240 nM) assays with minimal reaction system (1ul) and lower mismatch rates than traditional PCR technologies. Furthermore, the cost per test can be as low as $0.60.^718,719^ The ADESSO (Accurate Detection of Evolving SARS-CoV-2 through SHERLOCK Optimization) exhibits greater sensitivity (96%) and speed (15 min) compared to SHERLOCK while detecting SARS-CoV-2 variants.^720^ Furthermore, the Real-Time-Multiplexed SHERLOCK assay (real-time SLK) developed by Pena et al. enables simultaneous detection of multiple samples with sensitivity down to 5 copies/μL and a time to result of under 30 min.^721^ The CRISPR-based detection is not limited to viruses, but has also been expanded to the detection of other microorganisms, including bacteria, fungi and parasites.^722–724^ This highlights its potential as an important next-generation detection technology.^725,726^

Most of the current detections are still in the disposable testing phase, lacking real-time monitoring capabilities, which limits their application in recurrent infections and inflammatory diseases. Some research has been devoted to the development of real-time monitoring functions of nanosensors.^727,728^ For instance, the implantation of nanosensors in the body can minimally invasively detect the level of adriamycin in the body in real time, which can be used to assess the clinical use of the drug.^729^ Wearable nanobiosensors such as microneedle skin patches are more readily available for non-invasive detection of important markers in the human body.^730,731^ Nanomaterials have also demonstrated excellent properties as biomarkers in imaging techniques, such as MNPs that can act as contrast agents for MRI, enhancing the image signal and improving the picture quality.^732,733^ The current focus should be on reducing its toxicity or improving the renal clearance of the contrast agent, which is beneficial for its long-term development and clinical introduction.^734^ Besides, Nano-metal-organic frameworks (nano-MOFs) labeled with radionuclides have shown great potential in the anticancer field.^735^ Perhaps this technique could also be adapted to detect infectious or inflammatory diseases to increase the sensitivity and specificity of the test. Non-invasive imaging technology represents the path toward reducing harm in the future. For instance, nanotechnology has been integrated with “smart capsule” technology for the purpose of monitoring intestinal health.^736^ These recent advancements and future directions will offer greater convenience for the detection and diagnosis of infectious and inflammatory diseases.

Currently, overcoming the toxicity of NPs and enhancing their safety remains a key challenge. There are several optimization measures that can be implemented, such as adjusting dosage and exposure times sensibly to ensure benefits outweigh risks. In-depth research into solutions for the biocompatibility, toxicity, and stability of nanomaterials is essential to ensure their safe application. In addition, improving biocompatibility and reducing potential risks to the human body can be achieved through appropriate surface modifications and designs. The development of more intelligent and precise NP drug delivery systems, as well as the creation of optical, magnetic, and environmentally responsive nanomaterials, can enhance drug delivery efficiency and targeting.^737,738^

In recent years, artificial intelligence (AI) has shown immense potential in predicting and assessing the toxicity of nanomaterials. A recent systematic study demonstrated that machine learning algorithms can predict the cytotoxicity of nanomaterials based on their physicochemical properties, thus guiding the optimization of nanomaterial designs.^739,740^ For example, researchers developed the NanoTox model, which uses features like particle size and zeta potential to predict the toxicity of metal oxide NPs.^741^ Another study showcased how deep learning models can enhance the interpretation of immune responses and lung burdens caused by NPs, providing support for nanosafety assessments.^742^ Furthermore, researchers employed machine learning-assisted single-vessel analysis techniques to evaluate the permeability of NPs in tumor vasculature, offering guidance for optimizing the targeted delivery of nanodrugs.^743^ In summary, AI provides crucial tools for understanding the interactions between nanomaterials and biological systems and lays the foundation for establishing efficient and cost-effective nanotoxicity assessment systems. Future research is expected to utilize cutting-edge technologies like multiscale modeling and deep learning, leveraging existing data to train predictive models for real-time forecasting of complex biological effects, thereby supporting the translation of nanomedicine applications.

In summary, nanotechnology is playing an increasingly crucial role in the prevention, diagnosis, and treatment of infectious and inflammatory diseases. Relevant research is rapidly advancing and is supported by advanced technologies such as genomics, computational science, and AI. Harnessing the advantages of nanotechnology to address safety and other challenges will contribute to an overall improvement in the treatment of infectious and inflammatory diseases. Interdisciplinary collaboration is the key to achieving this goal. With further advancements in technology and research, it is believed that nanomedicine will shine brightly in addressing infectious and inflammatory diseases.
